# Quantum-inspired encoding enhances stochastic sampling of soft matter systems

**DOI:** 10.1126/sciadv.adi0204

**Published:** 2023-10-25

**Authors:** Francesco Slongo, Philipp Hauke, Pietro Faccioli, Cristian Micheletti

**Affiliations:** ^1^ Scuola Internazionale Superiore di Studi Avanzati (SISSA), Via Bonomea 265, I-34136 Trieste, Italy.; ^2^ Pitaevskii BEC Center, Department of Physics, University of Trento, Via Sommarive 14, I-38123 Povo, Trento, Italy.; ^3^ INFN-TIFPA, Via Sommarive 14, I-38123 Povo, Trento, Italy.; ^4^ Department of Physics and BiQuTe Center, University of Milano-Bicocca, Piazza della Scienza 3, I-20126 Milan, Italy.; ^5^ Department of Physics, University of Trento, Via Sommarive 14, I-38123 Povo, Trento, Italy.

## Abstract

Quantum advantage in solving physical problems is still hard to assess due to hardware limitations. However, algorithms designed for quantum computers may engender transformative frameworks for modeling and simulating paradigmatically hard systems. Here, we show that the quadratic unconstrained binary optimization encoding enables tackling classical many-body systems that are challenging for conventional Monte Carlo. Specifically, in self-assembled melts of rigid lattice ring polymers, the combination of high density, chain stiffness, and topological constraints results in divergent autocorrelation times for real-space Monte Carlo. Our quantum-inspired encoding overcomes this problem and enables sampling melts of lattice rings with fixed curvature and compactness, unveiling counterintuitive topological effects. Tackling the same problems with the D-Wave quantum annealer leads to substantial performance improvements and advantageous scaling of sampling computational cost with the size of the self-assembled ring melts.

## Teaser

Polymer systems hard for real-space Monte Carlo are treated by mapping to a spin model solved on classical and quantum computers.

## INTRODUCTION

Over the last few years, quantum computing has made enormous strides forward. Still, the central focus of the field continues to be the demonstration of quantum advantage in the form of a quantifiable speed-up for any specific practical application (*1*–*5*). An at least equally important question, and one with a potentially farther-reaching scope, is: Can research in quantum computing produce scientific advantages by opening up routes that would otherwise not have been on the horizon? Although classical supercomputers may not yet be outperformed by the current quantum computers, reformulating physical problems in a way that quantum hardware can handle may lead to innovative approaches with groundbreaking implications (*6*–*9*).

Past computing innovations have already led to algorithms that, while initially intended for specialized hardware, have subsequently sparked revolutionary modeling and simulation paradigms. A notable example is the development of lattice-Boltzmann schemes (*10*, *11*), invented to exploit supercomputing architectures with local topology. The use of lattice discretization in fluid dynamics made it possible to tackle previously unaddressed physical systems, such as binary fluids (*12*): What was initially introduced as an algorithmic innovation became a paradigm in physical modeling.

In the context of quantum hardware, a standard algorithmic framework is the quadratic unconstrained binary optimization (QUBO), through which a given problem is reformulated in terms of a set of Ising spins with at most pairwise interactions (*13*). The coupling constants of the linear and quadratic terms in the generalized Ising Hamiltonian are defined so that the low-energy states encode the properties of the problem under consideration. Although these low-energy states can also be identified with classical optimization algorithms (*14*), the QUBO framework provides a natural encoding for quantum annealing machines (*15*–*19*). It is a critical question whether physical problems exist for which adopting the QUBO formalism can play a transformative role, even when resorting to conventional classical computers.

In the first part of this work, we answer this question in the affirmative. We show that the QUBO encoding makes it possible to efficiently sample classes of many-body systems paradigmatically hard for conventional Monte Carlo (MC) or molecular dynamics (MD) simulations, such as the self-assembly (*20*) of melts of rigid ring polymers. Furthermore, unlike conventional sampling in real space, the QUBO encoding allows for seamlessly adding or removing physical constraints to the sampling, such as fixing the stiffness, density, and compactness of the rings in the melt to any desired value. We demonstrate that, without changing the complexity class of the problem, it is possible to direct sampling toward any region of parameter space, including those sparsely populated at equilibrium, e.g., ring curvatures at 10 or more standard deviations (SDs) from the equilibrium average. Thus, a more favorable scaling performance is achieved for ring melts of several hundred monomers compared to advanced MC sampling methods in real space. Harnessing this targeted QUBO sampling, we discover a counterintuitive effect of chain stiffness on the linking probability of ring polymers in self-assembled melts.

In the second part of this work, we show that the above advantages of the QUBO-based sampling with classical solvers can be enhanced by using dedicated quantum annealing machines (*19*, *21*–*23*). Using the D-Wave quantum solver for assembling maximally packed ring melts results in substantial computational time savings compared to classical solvers based on simulated annealing or MC and MD approaches. This performance improvement grows with system size *N*, resulting in a favorable ∼*N*^3^ scaling of the computational cost for sampling polymer melts in the considered regimes.

## RESULTS

### QUBO sampling of self-assembled ring polymers

Our QUBO-encoded system is inspired by supramolecular self-assembly where limited-valence particles attach to one another, forming extended polymer-like structures (*24*, *25*) in varying number and size, as dictated by thermodynamic conditions, e.g., particle density. This differs from conventional real-space MC or MD simulations of polymer systems, where chain length and backbone connectivity are permanently fixed.

Lattice models are used to mitigate the above sampling challenges for the following reasons. Piecewise linear chain contours can be naturally mapped to much longer polymers where the degrees of freedom at the finer scales have been integrated out (*26*–*28*). In addition, the discrete embedding space lends naturally to efficient treatment of excluded volume interactions. This property, in turn, facilitates the design and implementation of efficient nonphysical MC moves to relax crowded systems (*29*–*35*), making it possible to generate viable equilibrated initial conditions for continuum approaches (*36*, *37*), or establish scaling relationships otherwise beyond reach of off-lattice models (*38*).

**Fig. 1.**
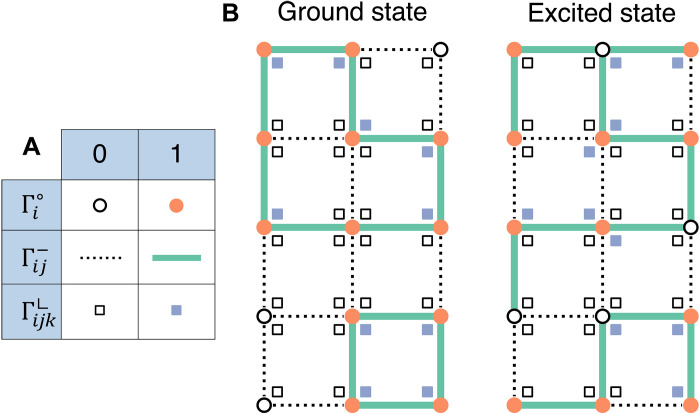
QUBO encoding of ring assembly. (**A**) Schematic representation of active/inactive binary variables corresponding to lattice sites (${\text{Γ}}_{i}^{\circ }$), edges (${\text{Γ}}_{ij}^{-}$), and corners (${\text{Γ}}_{ijk}^{⌞}$). (**B**) Examples of correct (left) and incorrect (right) solutions on a 5 × 3 lattice of the ring-assembly QUBO problem encoded by ℋ*_N_* for *N* = 12, see also section S2.

For the QUBO-based formulation of lattice models of ring polymers, we follow (*39*) and introduce binary variables, or equivalently Ising spins, to denote the occupied or empty state of sites and edges. As sketched in Fig. 1, single-site variable ${\text{Γ}}_{i}^{\circ }$ is 0 (inactive) or 1 (active) if site *i* is empty or occupied by a monomer, respectively. Likewise, the presence of a bond at the edge joining neighboring sites *i* and *j* > *i* is encoded by ${\text{Γ}}_{ij}^{-}$. Finally, ancilla binary variables are introduced for triplets of sites at lattice corners, ${\text{Γ}}_{ijk}^{⌞}$, *j* being the nearest neighbor of sites *i* and *k* > *i*.

With this proviso (*39*), each and all configurations of self-assembled ring polymers of *N* monomers are in one-to-one correspondence with the degenerate ground states of the following QUBO Hamiltonian

$$\begin{array}{c} H_{N}=A_{m}\;{\left({\text{Σ}}_{i}{\text{Γ}}_{i}^{\circ }-N\right)}^{2}+A_{b}\;{\left({\text{Σ}}_{\left\langle ij\right\rangle }{\text{Γ}}_{ij}^{-}-N\right)}^{2}\\ +A_{\mathrm{mb}}\;{\text{Σ}}_{\left\langle ij\right\rangle }{\text{Γ}}_{ij}^{-}\;(2-{\text{Γ}}_{i}^{\circ }-{\text{Γ}}_{j}^{\circ })+A_{c}\;{\text{Σ}}_{\left\langle ijk\right\rangle }{\text{Σ}}_{\left\langle ljm\right\rangle }^{\prime }{\text{Γ}}_{ijk}^{⌞}{\text{Γ}}_{ljm}^{⌞}\\ +A_{\mathrm{bc}}\;{\text{Σ}}_{\left\langle ijk\right\rangle }\;[3{\text{Γ}}_{ijk}^{⌞}+{\text{Γ}}_{ij}^{-}{\text{Γ}}_{jk}^{-}-2{\text{Γ}}_{ijk}^{⌞}\;({\text{Γ}}_{ij}^{-}+{\text{Γ}}_{jk}^{-})] \end{array}$$ (1)

where summations run over distinct pairs of neighboring sites (⟨*ij*⟩) and corner triplets (⟨*ljm*⟩), the prime indicates ⟨*ljm*⟩ ≠ ⟨*ijk*⟩, and the *A*’s are strictly positive coefficients so that each of the five quadratic terms is nonnegative. With respect to the formulation of Micheletti *et al.* (*39*), the basic QUBO Hamiltonian of Eq. 1 dispenses with binary variables associated to collinear triplets, thus requiring ∼20% fewer binary variables on cubic lattices at arbitrary filling.

By construction, viable assembled configurations correspond to binary variables that set to zero each term, thus yielding the ground-state energy, *ℋ_N_* = 0. Ground-state solutions correspond to lattice configurations with *N* occupied sites (first term), *N* occupied edges (second), each bonding two monomers (third), and no more than two incident bonds per monomer (fourth and fifth terms). A ground-state solution can be directly rendered as a polymer configuration by “marking” the lattice edges associated to nonzero ${\text{Γ}}_{ij}^{-}$ variables. The distinct chains in the configurations can be traced based on the site–site adjacency matrix encoded by the active ${\text{Γ}}_{ij}^{-}$ variables.

Knowing a priori the ground-state energy allows for identifying viable solutions of the energy minimization step, typically carried out with simulated annealing protocols. The annealing performance can be enhanced by suitable choices of the nonnegative coefficients of the quadratic energy terms (see Supplementary Text).

As we show here, different types of physical constraints can be seamlessly added to the QUBO Hamiltonian of Eq. 1. This perspective permits performant sampling even in highly constrained and dense regimes. Specifically, we consider (separate or concurrent) constraints on the total curvature and melt’s compactness. These can be enforced by fixing the total number of corner turns *n*_corners_ and contacts *n*_contacts_ by adding suitable positive quadratic forms to ℋ*_N_*

$$H=H_{N}+H_{\mathrm{contacts}}+H_{\mathrm{curvature}}$$ (2)

where

$$H_{\mathrm{curvature}}=A_{\mathrm{curv}}{\left({\text{Σ}}_{\left\langle ijk\right\rangle }{\text{Γ}}_{ijk}^{⌞}-n_{\mathrm{corners}}\right)}^{2}$$ (3)

$$\begin{array}{c} H_{\mathrm{contacts}}=A_{\mathrm{cont}}^{\left(0\right)}({\text{Σ}}_{\left\langle ij\right\rangle }{\text{Γ}}_{ij}^{B}-n_{\mathrm{contacts}}{)}^{2}\\ +A_{\mathrm{cont}}^{\left(1\right)}{\text{Σ}}_{\left\langle ij\right\rangle }\;[4{\text{Γ}}_{ij}^{A}+2{\text{Γ}}_{i}^{\circ }{\text{Γ}}_{j}^{\circ }-3({\text{Γ}}_{i}^{\circ }+{\text{Γ}}_{j}^{\circ }){\text{Γ}}_{ij}^{A}]\\ +A_{\mathrm{cont}}^{\left(2\right)}{\text{Σ}}_{\left\langle ij\right\rangle }\;(2{\text{Γ}}_{ij}^{B}+{\text{Γ}}_{ij}^{A}-{\text{Γ}}_{ij}^{-}{\text{Γ}}_{ij}^{A}+2{\text{Γ}}_{ij}^{-}{\text{Γ}}_{ij}^{B}-3{\text{Γ}}_{ij}^{A}{\text{Γ}}_{ij}^{B}) \end{array}$$ (4)

${\text{Γ}}_{ij}^{A}$ and ${\text{Γ}}_{ij}^{B}$ are ancilla binary variables (*40*) that are required because ℋ_contacts_ cannot be written in quadratic form with the sole variables *ℋ_N_*.

**Fig. 2.**
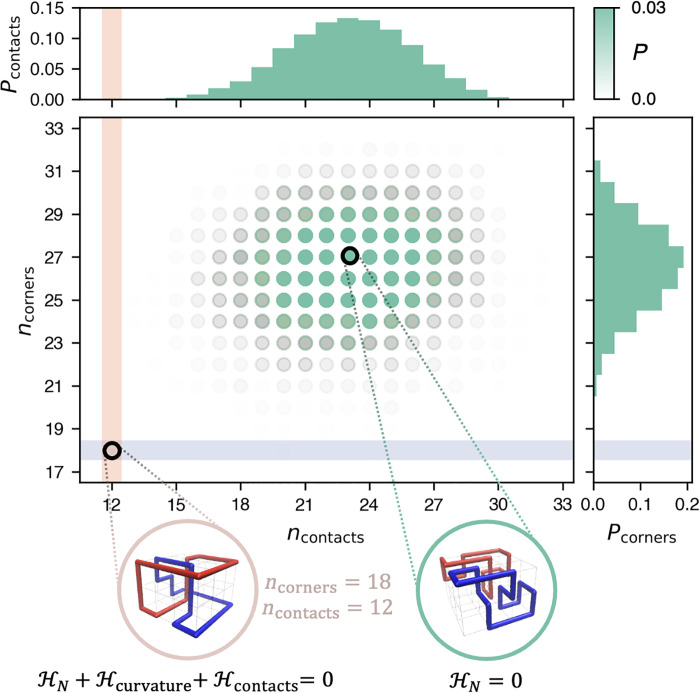
QUBO-based sampling of rare states. Probability density and marginal distributions of the number of corner turns and contacts of self-assembled rings on a 4 × 4 × 3 lattice at 2/3 filling fraction, obtained from 10^4^ samples minimizing *ℋ_N_*, as exemplified by the circled configuration on the right, where distinct rings are differently colored. The additional Hamiltonian terms of Eq. 2 enable the direct sampling for atypical, and hence very rare, combinations of curvature and contacts, e.g., at the intersection of the shaded bands, as exemplified by the circled configuration on the left.

As an example, Fig. 2 presents data for 10^4^ microstates sampled by repeatedly minimizing *ℋ_N_* for a small lattice at 2/3 filling fraction. The data show the joint equilibrium distribution of total curvature and contacts of assembled rings. Microstates with extremal values of curvature (*n*_corners_ = 18) and contacts (*n*_contacts_ = 12) are rarely populated at equilibrium using the Hamiltonian in Eq. 1. Yet, they can be directly sampled by minimizing the extended Hamiltonian of Eq. 2.

### Application to entangled rings melts

We now show that this QUBO formulation enables us to characterize the entanglement of topologically unrestricted ring polymers at maximum packing density. This problem is crucial in various research areas, including the design of mechanically bonded metamaterials (*41*), aging of active topological glasses (*42*), anomalous viscoelasticity of ring melts (*43*), and restructuring of chromosomes by topoisomerases (*44*, *45*). Despite its relevance, we still lack a general understanding of basic aspects of the problem, such as how the incidence of ring–ring entanglement (linking) varies with system size or ring curvature. At least in part, this is due to the fact that the computational cost of sampling with conventional MC or MD methods grows rapidly with system density. Differently, in our QUBO formulation, the space-filling constraint not only does not increase, but even reduces the complexity of the problem compared to smaller densities. This is because the binary variables associated with lattice sites and contacts are all “active” at complete filling and therefore can be dropped from the Hamiltonian.

**Fig. 3.**
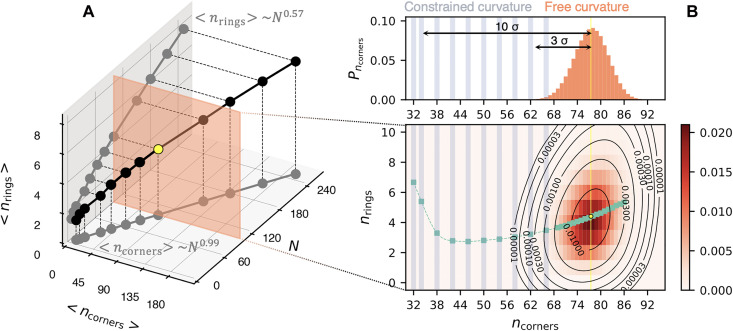
Ring melts with fixed and free curvature. (**A**) Average number of rings and corner turns (curvature) of space-filling rings assembled in cuboids of size *N*. Data points are averages over 10^4^ states or more. (**B**) Probability distribution, with smoothed contour lines, computed from >3 × 10^5^ states minimizing ℋ*_N_* on a 5 × 5 × 4 cuboid (*N* = 100). The marginal curvature distribution (top graph) has SD σ_corners_ = 4.3. We addressed rare states, from 3 to >10σs from the average (yellow), by minimizing ℋ*_N_* + ℋ_curvature_, typically collecting 10^4^ states at given *n*_corners_ (blue bands). Green data points and spline show the average number of rings computed using states without (with) fixed curvature close to (far from) the modal value.

As a first step, we discuss the equilibrium properties of assembled melts filling cuboids of increasing size *N*. The results, obtained by minimizing *ℋ_N_*, are shown in Fig. 3A. The average total ring curvature is found to scale approximately as ⟨*n*_corners_⟩ ∝ *N*^0.57^, while the number of rings grows as ⟨*n*_rings_⟩ ∝ *N*^0.99^, indicative of extensivity.

Figure 3B details the probability distribution of the two observables, computed with an extensive sampling of microstates for a given lattice size, *N* = 100. The bivariate distribution is unimodal, peaked around the ensemble averages, ⟨*n*_corners_⟩ = 77.5 and ⟨*n*_rings_⟩ = 4.4.

The corresponding marginal probability distribution covers only a limited fraction of the admissible range of *n*_corners_: geometric arguments indicate that, for *N* = 100, *n*_corners_ can range from a maximum of 100 down to 32, respectively, at approximately 5.2 and 10.5 SDs from the modal value.

### Sampling ring melts with and without curvature constraints

**Fig. 4.**
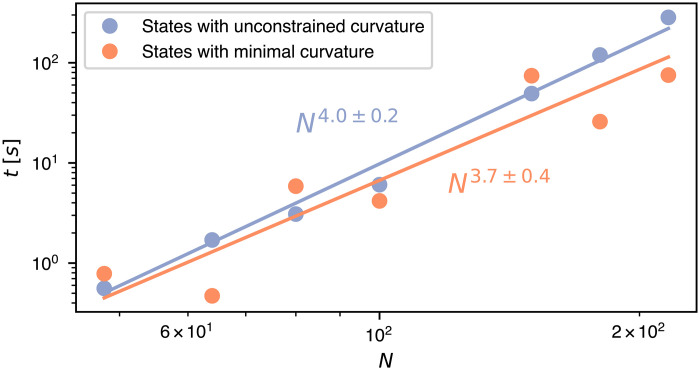
QUBO-based sampling with and without curvature constraints. Characteristic run time required by the D-Wave classical solver to generate ring melts filling cuboids of size *N* with and without the quadratic constraint of minimal curvature. The times refer to the D-Wave classical solver run on standard Intel-based workstations with optimized simulated annealing schedule and coefficients of the potential (see section S8). Estimated relative statistical errors are at most 15%. The indicated scalings are from power-law best fits to the data (solid lines).

The possibility of varying the average number of corners paves the way to investigate the effect of the chain’s stiffness in dense polymer melts. Our QUBO approach allows for directly targeting states at any curvature value, including the extremal ones, which are exponentially suppressed by entropic effects in the unconstrained ensemble. The targeted sampling can be accomplished by simply supplementing ℋ*_N_* with ℋ_curvature_, a straightforward operation that does not require increasing the number of binary variables, a key determinant of computational complexity. The plot of Fig. 4 presents the size-dependent run times for generating space-filling melts with the constraint of minimal curvature and without it. Depending on how the added quadratic terms sculpt the *N*-dependent QUBO energy landscape, the computational cost of sampling states with added constraints can be larger or smaller than the unconstrained case while remaining comparable to it.

For instance, the minimal curvature states for *N* = 100, which have a Gaussian estimated probability of <10^−19^, can be generated with a typical run time of 4.2 s on standard workstations, while 6.1 s is required for the unconstrained case. Because of geometric effects, the order can be reversed for other sizes, such as *N* = 150. Overall, the performance over the entire considered range 48 ≤ *N* ≤ 216 is remarkably similar and, in both cases, compatible with an *N*^4^ scaling. These results strongly suggest that the computational complexity class of the QUBO-based sampling is unchanged by the addition of even stringent physical constraints.

### Linking probability in space-filling ring melts

The facile extension of QUBO-based sampling to space-filling and arbitrary curvature constraints naturally lends itself to tackle previously unexplored problems. To illustrate this potential, we investigate the incidence of interchain entanglement, i.e., topological links, in melts with varying effective rigidity of the self-assembled rings. This question is a generalization of the problem of intrachain topological entanglement, or knotting, in space-filling curves, which has bearing in biological contexts such as DNA packaging in viral capsids (*46*, *47*) and in soft matter ones related to polymers and self-assembling meta-materials in confinement (*48*–*52*). Extending considerations to the interlocking of self-assembled rings offers a timely reference also for challenging biological systems, such as the linked DNA ring assemblies of kinetoplasts (*53*) and strand crossings in DNA bundles operated by topoisomerase enzymes (*44*, *45*, *54*).

**Fig. 5.**
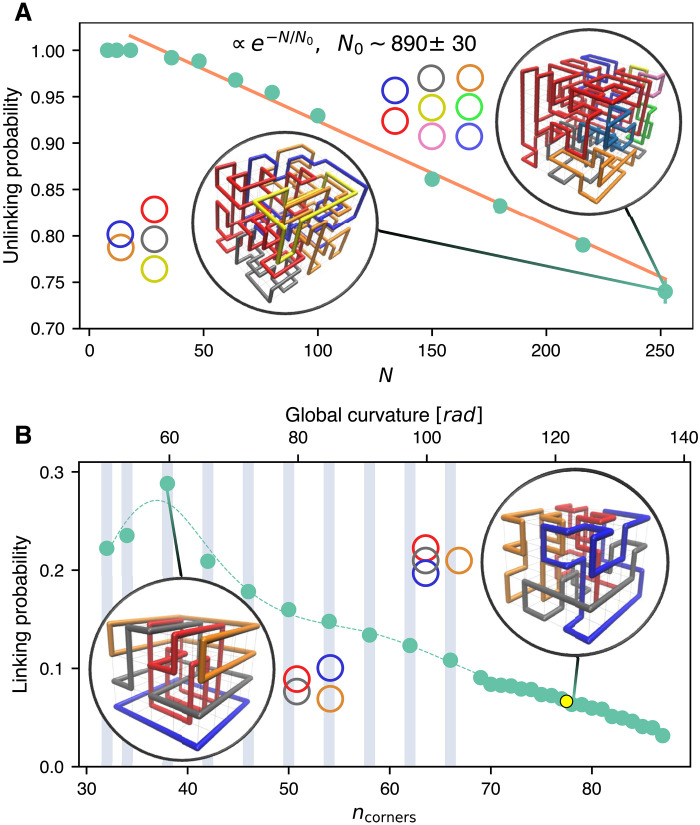
Linking properties of space-filling ring melts. (**A**) Probability that all ring pairs in space-filling ring melts of size *N* are unconcatenaned, i.e., with zero Gaussian linking number. Single rings, i.e., Hamiltonian cycles, are excluded. (**B**) Complementary linking probability versus curvature for a 5 × 5 × 4 lattice, *N* = 100, with blue bands denoting sampling at fixed curvature (the line is a spline to the data points). The yellow point marks the equilibrium ensemble average. Circled are typical configurations at the indicated values of *N* and *n*_corners_, where distinct rings are differently colored. The rings’ linked state is schematically represented on the side. Counterintuitively, increasing the effective ring stiffness can substantially enhance linking.

Figure 5A presents the probability that no homological link is present in a sampled microstate of rings assembled at complete filling. The unlinking probability decays exponentially with system size, with a characteristic length scale of *N*_0_ ∼ 890; see Supplementary Text where the decay of the knotting probability is also presented.

Figure 5B illustrates how the abundance of links varies with ring curvature. With respect to the plain, fully flexible ensemble, increasing ring stiffness, i.e., lowering ⟨*n*_corners_⟩, can yield a fivefold increase of *p*_link_. Thus, the data reveal the unexpected and counterintuitive effect that increasing the effective ring stiffness can substantially enhance, rather than suppress, linking. The same conclusion holds for alternative definitions of interchain entanglement (see section S7). Incidentally, the results generalize previous studies of rigidity-enhanced intrachain entanglement (knotting) in free and noncompact polymers, interpreted as due to the emergence of large loops prone to threadings (*55*–*57*).

The fact that interchain entanglement can be boosted by increasing ring stiffness sheds light on a problem that had not been tackled before, neither off- nor on-lattice, despite having implications in various contexts, from rationalizing the effect of mere spatial confinement or molecular crowding on the decatenation of newly-replicated DNA (*44*, *45*), to inspiring optimized designs of supramolecular catenanes (*58*) based on the rigidity of repeating units.

### Constrained sampling: QUBO encoding versus real-space MC

Sampling systems with multiple layers of physical constraints, such as ring melts at a given density and curvature or bending rigidity, is a challenge for MC approaches formulated in real space. The acceptance rate of monomer and bond displacements degrades rapidly with the addition of each constraint of chain connectivity, self-avoidance, high packing density, and bending rigidity, following the progressive reduction of accessible conformational space. In lattice contexts, the shrinking of the conformational space is reflected in the drop of the connectivity constant going from, e.g., random walks to self-avoiding (*59*, *60*) and then Hamiltonian walks (*61*, *62*). By contrast, QUBO-based approaches are ideally suited to tackle multiply constrained systems, not only because additional quadratic constraints can be straightforwardly added (*13*), but especially for the fact noted above that sampling run time does not diverge, and can even improve upon adding such terms.

**Fig. 6.**
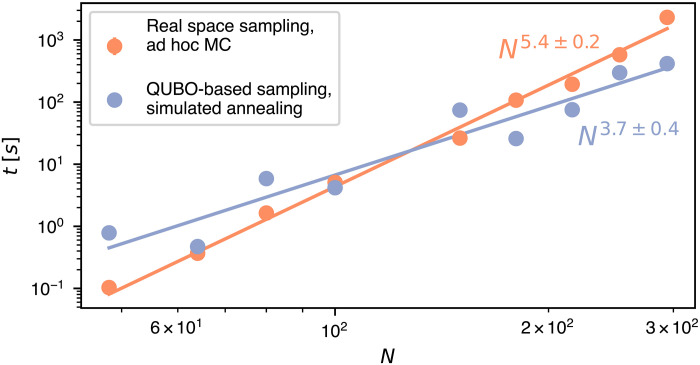
QUBO-based sampling versus ad hoc real-space MC. Characteristic run time per independent sample required by QUBO-based and real-space MC schemes to generate minimal-curvature states filling cuboids of size *N*. The QUBO-based run time is based on the D-Wave neal classical solver with optimized annealing schedule and Hamiltonian coefficients. The real-space MC run time is based on a replica exchange scheme with optimized temperatures and exchange rates and using plaquette-flip moves ad hoc tailored for cubic lattices at complete filling. The run times were measured on a standard Intel-based workstation, and details of the optimized algorithms are provided in section S8. Estimated relative statistical errors are at most 15%. The indicated scalings are from power-law best fits to the data (solid lines).

The advantages of working in the abstract space of QUBO binary variables rather than in real space are illustrated in Fig. 6 for the challenging case of ring melts of minimal curvature in fully filled cuboids of size *N*. The QUBO-based data are the same as previously discussed in Fig. 4, which were obtained with a general simulated annealing protocol. To avail of the best-performing algorithms for real-space sampling, we used a replica exchange method (*63*, *64*) based on long-range plaquette-flip MC moves (see section S9). The plaquette-flips are long-range moves (*33*, *65*, *66*) specifically designed for cubic lattices at complete filling, where they allow for changing the number, length, and conformation of the rings without incurring in rejections. These unique advantages cannot be reaped on general lattices or on cubic ones at partial filling, where plaquette-flip moves are not ergodic. These features make particularly stringent the comparison with the QUBO-based sampling plus simulated annealing, which is a more generally applicable combination for sampling.

In the examined regime of Fig. 6, the performance of the specialized real-space sampling with ad hoc moves scales approximately as ∼*N*^5^, while the general QUBO-based one has the more favorable scaling of ∼*N*^4^, as noted above. Thanks to the scaling advantage, the latter scheme outperforms the former already for lattices with *N* ∼ 200.

### Quantum versus classical solvers

**Fig. 7.**
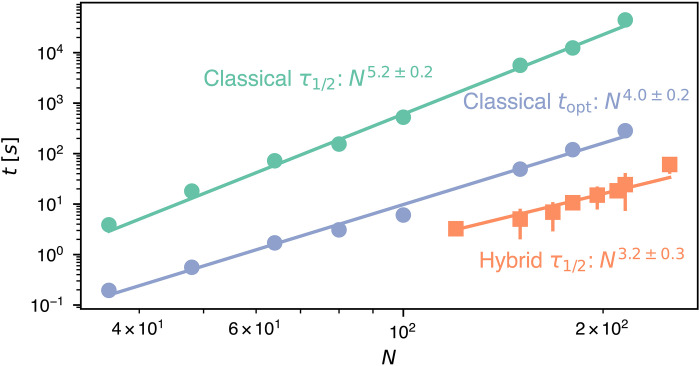
Classical simulated annealing versus hybrid quantum QUBO solver. Characteristic run times required by D-Wave’s purely classical and hybrid (classical-quantum) solvers to minimize ℋ*_N_* for filled cuboids of size *N*. The τ_1/2_ data for both types of solvers correspond to the run times yielding a 50% hit rate with default parameters. The *t*_opt_ data are the same as in Fig. 4 and correspond to the characteristic run times per independent sample required by the classical solver with an optimized annealing schedule. The indicated scalings are from power-law best fits to the data (solid lines).

The QUBO-based formulation of polymer sampling is purposely primed for use with quantum annealing machines. Using the D-Wave hybrid (classical-quantum) QUBO solver, we observe major improvements over classical solvers based on simulated annealing, again in the D-Wave implementation. Figure 7 compares the characteristic run times for generating space-filling melts with the hybrid solver and with the classical one. To best assess the potential of the latter, we also considered an optimized annealing schedule besides the default one, while the hybrid solver was exclusively used with default settings.

As the data in Fig. 7 show, for the considered range of *N*, hybrid solvers improve the sampling rate by up to three orders of magnitude compared to the classical solver with default settings, and by up to one for the optimized schedule. In both cases, the difference increases with the considered system size: While the optimized classical performance grows as ∼*N*^4^, the one of the hybrid solver scales as ∼*N*^3^. This notable improvement sets the premises for treating complex polymer systems well beyond the sizes addressable by conventional real-space sampling.

In this regard, we recall that, in general MC and MD approaches, the sole evaluation of excluded-volume interactions has a cost of order *N*^2^. Performative algorithms include MC sampling of “crossing rings” at half-filling fraction (*67*, *68*), where the physical relaxation times of individual rings grow at least quadratically with their contour length, approaching the relaxation dynamics of Rouse chains. This quadratic estimate can be ported to our system, where assembled rings’ length scales as *N*^0.39^ (see section S6). Compounding it with the aforementioned *N*^2^ expenditure yields an estimated lower bound of ≃*N*^2.78^ for the cost of decorrelating single rings in the melt. The observed *N*^3^ scaling of hybrid solvers, which instead refers to the entire melt, compares well with the above bound.

Further comparisons can be made with Hamiltonian paths, for which several generation methods exist, from exhaustive enumeration to stochastic sampling with and without heuristic speed-ups (*32*, *69*–*73*). The unbiased (ergodic) method of Mansfield (*33*) has achieved a remarkable ∼*N*^3^ cost for Hamiltonian cycles on hypercubic lattices by harnessing features unique to such embeddings at complete filling, analogously to the plaquette-flip moves discussed before. The reported scaling is thus a best-case scenario, given that the system-specific improvements are not transferable to different filling fractions or different regular or irregular lattices. Instead, the QUBO-based sampling is seamlessly applicable to all such diverse contexts. Considering this and the simplicity of adding physical constraints to the QUBO model, the observed ∼*N*^3^ performance of the hybrid solver is remarkable.

We conclude by noting that the improved scaling exponent of the performance of the hybrid solver compared to classical annealing should not be construed as a demonstration of quantum advantage. In particular, it cannot be ruled out that the quantum-based scaling could be matched or surpassed by performative classical algorithms, including those that might be foreseeably developed for particular versions of our QUBO-based models. Such solvers, besides improving the rate of returning QUBO solutions, should satisfy the stringent requirement of uniform coverage of the ground-state manifold. As we verified for systems with few exactly enumerable states, this requirement is not necessarily met when heuristic speed-ups are used in otherwise general optimization strategies (see section S5). Instead, we verified for the same systems that uniform sampling is achieved with classical minimizers based on simulating-annealing as well as quantum ones.

## DISCUSSION

In summary, we have introduced a QUBO-based sampling of many-body systems to demonstrate that, even when resorting to purely classical machines, quantum-inspired encoding can lower the computational complexity of soft matter problems hard to tackle with conventional, real-space sampling methods.

As a paradigmatic case, we have considered the problem of sampling self-assembled ring polymers at various lattice filling fractions, with and without constraints for the number of contacts and total ring curvature. The QUBO model has enabled us to study melts of rings at maximum packing and high effective bending rigidity, reporting for the first time a stiffness-induced boost for interchain entanglement. Despite the ramifications in different fundamental and applicative contexts, this challenging problem has hitherto remained unexplored with real-space MC and MD sampling methods. Finally, we have shown that, even with the currently available architectures, quantum hardware can substantially enhance the sampling rate for challenging polymer systems, which, in the accessible regimes, is consistent with a more favorable resource scaling with system size than classical simulated annealing and real-space MC or MD approaches.

In our family of QUBO-encoded polymer models, multiple constraints, such as those on minimal curvature and complete filling, can be added seamlessly and without altering the computational complexity of the problem. These advantageous properties hinge on general features of the QUBO encoding and classical annealers. Thus, we anticipate that they ought to unlock the study of a broader repertoire of constrained physical systems. We hope the results can also inform and stimulate the development and application of dedicated solvers based on the QUBO formulation, such as digital annealers (*6*, *74*, *75*), especially with an eye on assessing whether the enhancement of polymer sampling with hybrid annealers can be matched or even improved upon. Either outcome will be exciting and consequential: Finding a class of physically relevant models where a practical quantum advantage emerges would mark a milestone in the development of scientific applications of quantum computing; On the other hand, in the opposite scenario, the benchmarks based on quantum-inspired encodings will have substantially pushed forward the boundaries of classical algorithms and modeling, providing a well-defined challenge for quantum computing application.

## MATERIALS AND METHODS

### QUBO-based sampling with classical and quantum solvers

The QUBO Hamiltonians for self-assembled rings at various filling fractions of the cubic lattice, with or without constrained total curvature, were minimized with classical, quantum, and hybrid classical-quantum solvers. For the classical solver, we used the neal minimizer developed by D-Wave, which is based on simulated annealing. Annealing runs for *N* ≤ 100 were performed on the D-Wave Leap platform, while for longer systems, we used a standard Intel-based workstation. In the latter case, the workstation run time was converted to the Leap-equivalent one based on the scaling of run time with the number of sweeps. For the hybrid and fully quantum solvers, we respectively used the LeapHybridSampler and DWaveSampler minimizers developed by D-Wave, which were run exclusively on the Leap platform. The performance of the QUBO-based sampling of polymer systems was quantified with two characteristic measures of the time to solution, namely, τ_1/2_ and *t*_opt_. The former is the solver run time required to yield a correct solution (i.e., a ground state) in 50% of the trials. In general, this characteristic time does not coincide with the run time yielding the largest number of correct solutions per unit time, *t*_opt_, which was thus additionally considered. For purposes of maximum productivity, both the coefficients of the potentials, which shape the energy landscape, and the annealing schedules were optimized (section S8).

### Real-space sampling with the replica exchange method

A replica exchange method was implemented for the real-space sampling of ring melts in cuboids at complete filling and with the minimum curvature constraint. The conformations were evolved with plaquette-flips, a set of moves designed for cubic lattices at complete filling, where they are particularly efficient (section S9). The moves preserve the number of bonds while allowing the chain connectivity, the number of rings, and number of corners to fluctuate. The moves thus allow for exploring the same conformational space sampled with the QUBO models with unrestricted curvature, with the proviso of initializing the replica exchange system to a state with the correct number of bonds. The energy function was set equal to total curvature, i.e., the number of corners. The number of replicas and their temperatures were optimized for an efficient coverage of a broad range of energy (curvature) values. Exchanges were proposed at time intervals at least 100 times smaller than the autocorrelation time, $\bar{\tau }$, of the lowest-temperature replica, which served as a collector for the lowest-energy solutions. The autocorrelation time, $\bar{\tau }$, was used to compute the typical run time required per uncorrelated solution on a standard Intel-based workstation.
