# Supplementary material for: Quantum-inspired encoding enhances stochastic sampling of soft matter systems
Source: Sci Adv. 2023 Oct 25;9(43):eadi0204. doi: 10.1126/sciadv.adi0204 (PMC10599611; doi:10.1126/sciadv.adi0204)
Supplement: Supplementary file 1 — Supplementary Text Figs. S1 to S14 Tables S1 to S4 Legend for data file S1 [file sciadv.adi0204_sm.pdf]

Science Advances
AAAS

Supplementary Materials for

Quantum-inspired encoding enhances stochastic sampling of soft matter systems

Francesco Slongo *et al.*

Corresponding author: Cristian Micheletti, cristian.micheletti@sissa.it; Pietro Faccioli, pietro.faccioli@unimib.it

Sci. Adv. **9**, eadi0204 (2023)
DOI: 10.1126/sciadv.adi0204

The PDF file includes:

Supplementary Text
Figs. S1 to S14
Tables S1 to S4
Legend for data file S1

Other Supplementary Material for this manuscript includes the following:

Data file S1

1. GENERALITIES OF CLASSICAL AND QUANTUM QUBO SOLVERS

Solving quadratic unconstrained binary optimization (QUBO) problems involves finding the set of binary variables, $\sigma_{i=1\dots n} \in \{0,1\}$, minimizing an Ising-like Hamiltonian with at most quadratic interactions:

$$\mathcal{H} = \sum_{ij} J_{ij}\sigma_i\sigma_j + \sum_i h_i\sigma_i \tag{S1}$$

where the interaction matrix, J, and external-field vector, h, are assigned.

The go-to method for identifying the ground state(s) of $\mathcal{H}$ using classical computing is simulated annealing. In this framework the binary variables, $\sigma_{1\dots n}$, are initially randomised and then evolved with repetitive Metropolis-like updates while the effective temperature of the system is progressively lowered according to a pre-assigned schedule. In this approach, which is inherently stochastic, the probability of identifying the ground-state in any given annealing run increases as the cooling rate is lowered.

Annealing protocols, albeit of a different kind, are employed for solving the QUBO problem with dedicated quantum hardware, too. In this case, the physical system described by the binary variables is initially prepared in the known ground state of a soluble Hamiltonian $\mathcal{H}_0$, e.g. of non-interacting spins. The system is later evolved while, simultaneously, the time-dependent Hamiltonian is continuously varied from $\mathcal{H}_0$ to the target one $\mathcal{H}$. Asymptotically slow Hamiltonian transformations are guaranteed to yield ground state solution(s) of $\mathcal{H}$, while these will be generally found with only a finite probability in finite-time transformations.

In our study, we relied on algorithmic tools and platforms developed by D-Wave to solve the QUBO-problem using both classical and hybrid (classical-quantum) annealers. Specifically, for the classical annealing runs we resorted to the `neal` code while for the hybrid ones we resorted to the `leaphybridsolver`.

A detailed comparison of the performance of these classical and hybrid annealers on our QUBO-based models is provided in the last Section of the main text.

2. QUBO HAMILTONIAN

In the following we outline the derivation of our QUBO Hamiltonians starting from $\mathcal{H}_N$, the Hamiltonian for self-assembled rings based on the model of ref. *(39)*, and then extending considerations to constraints on curvature and number of contacts.

A. Basic Hamiltonian

$\mathcal{H}_N$ involves various types of binary variables which are mapped to distinct lattice sites (Γ°_i), lattice bonds (Γ^{-}_{ij}, $i\rangle j$), and lattice corners ($\Gamma^{\llcorner}_{ijk}$, where site j is nearest neighbor of sites i and $k\rangle i$). The active/inactive states of these variables correspond to values 1 and 0, respectively. The formulation applies to any regular or irregular lattice; for simplicity of visualization, in the following, we will refer to square lattices.

The basic Hamiltonian is:

$$\mathcal{H}_N = V_{\rm m} + V_{\rm b} + V_{\rm mb} + V_{\rm c} + V_{\rm bc}\ , \tag{S2}$$

where the various potential terms are

$$\begin{aligned} V_{\rm m} &= A_{\rm m}\left(\textstyle\sum_i \Gamma^{\circ}_i - N\right)^2\ , \\ V_{\rm b} &= A_{\rm b}\left(\textstyle\sum_{\langle ij\rangle}\Gamma^{-}_{ij} - N\right)^2\ , \\ V_{\rm mb} &= A_{\rm mb}\textstyle\sum_{\langle ij\rangle}\Gamma^{-}_{ij}(2-\Gamma^{\circ}_i-\Gamma^{\circ}_j) \\ V_{\rm c} &= A_{\rm c}\textstyle\sum_{\langle ijk\rangle}\sum'_{\langle ljm\rangle}\Gamma^{\llcorner}_{ijk}\Gamma^{\llcorner}_{ljm} \\ V_{\rm bc} &= A_{\rm bc}\textstyle\sum_{\langle ijk\rangle}\left[3\Gamma^{\llcorner}_{ijk} + \Gamma^{-}_{ij}\Gamma^{-}_{jk} - 2\Gamma^{\llcorner}_{ijk}(\Gamma^{-}_{ij}+\Gamma^{-}_{jk})\right] \end{aligned}$$

where subscripts m, b, and c stand respectively for monomer, bond and corner, $\langle ij\rangle$ denotes summation over distinct pairs of neighboring sites ($j > i$), and $\langle ijk\rangle$ over distinct site triplets corresponding to lattice corners, with j being the vertex and $k > i$. The prime indicates summation over distinct corner triplets ijk and ljm, which share the vertex. The A coefficients are positive.

By construction, the degenerate ground states of the Hamiltonian are in one-to-one correspondence with the viable configurations of self-assembled rings of N monomers in total.

To verify this, consider that viable configurations of the self-assembled rings must satisfy the following requisites:

1. N lattice sites are occupied. This implies that the number of active site variables (value equal to 1) must be equal to N: $\sum_i \Gamma^{\circ}_i = N$,

2. N lattice edges are occupied. This implies that the number of active edge variables must be equal to N: $\sum_{\langle ij\rangle}\Gamma^{-}_{ij} = N$,

3. each occupied lattice edge must connect two occupied sites. Thus, $\Gamma^{-}_{ij} = 1$ must be associated with $\Gamma^{\circ}_i = \Gamma^{\circ}_j = 1$,

4. no branching is allowed. Equivalently, each occupied lattice site should not have three or more occupied incident edges. This implies that there should not be any pair of active corner variables that share the same vertex.

5. For the above to apply, the active/inactive state of corner variables must be fully determined by the state of the edge variables. Specifically, a corner variable is active if and only if the two involved edge variables are both active.

Notice that the above combination of constraints makes it unnecessary to further require that each occupied site must have exactly two active incident edges, as this property is automatically satisfied.

Requirements 1, 2, and 3 are enforced by the quadratic forms $V_{\rm m}$, $V_{\rm b}$ and $V_{\rm mb}$, respectively. In fact, $V_{\rm m}$ is minimum when the number of active sites is equal to N, $V_{\rm b}$ is minimum when the number of active edges is equal to N, and $V_{\rm mb}$ is minimum when each active edge is associated to active sites. All three potentials are equal to zero at their minimum.

Instead, $V_{\rm c}$ enforces the exclusion of branching points, requirement 4, using energy penalties for any pair of active corner triplets with shared vertices. Conversely, configurations without branching do not incur in penalties and thus minimize $V_{\rm c}$. The minimum value of $V_{\rm c}$ is zero.

Requirement 5, namely the consistency of corner and edge binary variables, is enforced by $V_{\rm bc}$, whose minimization amounts to requiring that $\Gamma^{\llcorner}_{ijk} = \Gamma^{-}_{ij}\Gamma^{-}_{jk}$ for all triplets. In fact, the minimum of $V_{\rm bc}$, again equal to zero value, is attained in correspondence of viable combinations of the variables, while non-viable ones receive an energy penalty for all others. This property is made explicit in the truth table in Table. S1 The repertoire of possible viable and non-viable corner and edge combinations is sketched in Fig. S1.

	Case	Γ^{-}_{ij}	Γ^{-}_{jk}	$\Gamma^{\llcorner}_{ijk}$	$V_{\rm bc}/A_{\rm bc}$		$U_{\rm bc}/A_{\rm bc}$
Viable	A	0	0	0	0	Ground state	0
	B	1	0	0	0	Ground state	0
	C	0	1	0	0	Ground state	0
	D	1	1	1	0	Ground state	0
Non-viable	E	0	0	1	1	Excited state	1
	F	1	0	1	1/3	Excited state	1
	G	0	1	1	1/3	Excited state	1
	H	1	1	0	1/3	Excited state	1

TABLE S1. Truth table for the quadratic potential term $V_{\rm bc}$, referring to the eight distinct combinations of the involved binary variables. The combinations are labelled as in the sketches of Fig. S1. For reference, the last column reports the corresponding values of the non-quadratic potential $U_{\rm bc}$, which has the same ground states of $V_{\rm bc}$.

Incidentally, we note that the seemingly cumbersome expression for $V_{\rm bc}$ follows from the necessity to adopt a quadratic form, a fundamental requisite of QUBO formulations. If this requisite was omitted, the edge-corner consistency could be enforced more transparently by the compact term: $U_{\rm bc} = A_{\rm bc}\sum_{\langle ij\rangle}(\Gamma^{\llcorner}_{ijk} - \Gamma^{-}_{ij}\Gamma^{-}_{jk})^2$, whose truth table is also shown in Table S1.

Compared to ref. *(39)*, the basic Hamiltonian of Eq. S2 dispenses with the use of collinear site triplets, resulting in a $\sim 20\%$ saving of binary variables. The simpler Hamiltonian, besides being more transparent, is encoded by smaller h arrays and J matrices, which speeds up the search of solutions.

B. QUBO potentials for curvature and contacts constraints

Curvature constraint. On hypercubic lattices, edges can meet at 0 and $\pi/2$ angles only, the latter case corresponding to corner turns. Discounting the $\pi/2$ factor for simplicity, the total curvature of a microstate of self-assembled rings is simply given by number of active corner variables, $\sum_{\langle ij\rangle}\Gamma^{\llcorner}_{ijk}$. Accordingly, the QUBO constraint on total curvature, i.e. the total number of corners $n_{\rm corners}$, is straightforwardly enforced by the quadratic form:

$$\mathcal{H}_{\rm curvature} = A_{\rm curv}(\sum_{\langle ijk\rangle}\Gamma^{\llcorner}_{ijk} - n_{\rm corners})^2 \qquad (S3)$$

The approach can be extended to general types of regular lattices, where different sets of corner variables must be introduced for each of the possible angles formed by two incidents edges. In such general cases, the linear correspondence of number of active corner variables and total curvature is lost, and the curvature constraint must be enforced using a book-keeping of the number of active corners for each of the possible angles.

Contacts constraint. Our QUBO-based formulation of the self-assembling problem allows for counting the number of contact interactions of monomers in the rings, an observable that is commonly used to monitor or constrain the degree of compactness of polymer systems.

Establishing the number of contacts in a microstate amounts to counting the number of occupied sites that are nearest neighbours on the lattice but not bonded. In the QUBO model, this counting can be implemented with two ancilla*(40)* sets of binary edge variables, $\Gamma^{\rm A}_{ij}$ and $\Gamma^{\rm B}_{ij}$, and two auxiliary quadratic potentials V_0 and V_1,

$$\begin{aligned} V^{(1)}_{\rm cont} &= A^{(1)}_{cont}\sum\nolimits_{\langle ij\rangle}\left[4\Gamma^{\rm A}_{ij} + 2\Gamma^{\circ}_{i}\Gamma^{\circ}_{j} - 3\left(\Gamma^{\circ}_{i} + \Gamma^{\circ}_{j}\right)\Gamma^{\rm A}_{ij}\right] \\ V^{(2)}_{\rm cont} &= A^{(2)}_{cont}\sum\nolimits_{\langle ij\rangle}\left(2\Gamma^{\rm B}_{ij} + \Gamma^{\rm A}_{ij} - \Gamma^{-}_{ij}\Gamma^{\rm A}_{ij} + \ldots\right. \\ &\qquad \left.\ldots\, 2\Gamma^{-}_{ij}\Gamma^{\rm B}_{ij} - 3\Gamma^{\rm A}_{ij}\Gamma^{\rm B}_{ij}\right)\ . \end{aligned} \qquad (S4)$$

By construction, the potentials are minimized, with value equal zero, when $\Gamma^{\rm A}_{ij} = \Gamma^{\circ}_{i}\Gamma^{\circ}_{j}$ and $\Gamma^{\rm B}_{ij} = \Gamma^{\rm A}_{ij}\left(1 - \Gamma^{-}_{ij}\right)$. These two conditions imply that $\Gamma^{\rm B}_{ij} = 1$ if and only if $\Gamma^{\circ}_{i} = \Gamma^{\circ}_{j} = 1$ and $\Gamma^{-}_{ij} = 1$, that is when non-bonded neighbouring sites i and j are occupied.

The number of contacts is thus given by $\sum_{\langle ij\rangle}\Gamma^{\rm B}_{ij}$. Accordingly, the quadratic constraint on the number of contacts is enforced by minimizing

$$\mathcal{H}_{\rm contacts} = V^{(0)}_{\rm cont} + V^{(1)}_{\rm cont} + V^{(2)}_{\rm cont} \qquad (S5)$$

with

$$V^{(0)}_{\rm cont} = A^{(0)}_{cont}\left(\sum\nolimits_{\langle ij\rangle}\Gamma^{\rm B}_{ij} - n_{\rm contacts}\right)^2 \qquad (S6)$$

When the constraint is satisfied, $\mathcal{H}_{\rm contacts}$, takes on its minimum value, which is zero. Notice that the above formulation holds for general lattices.

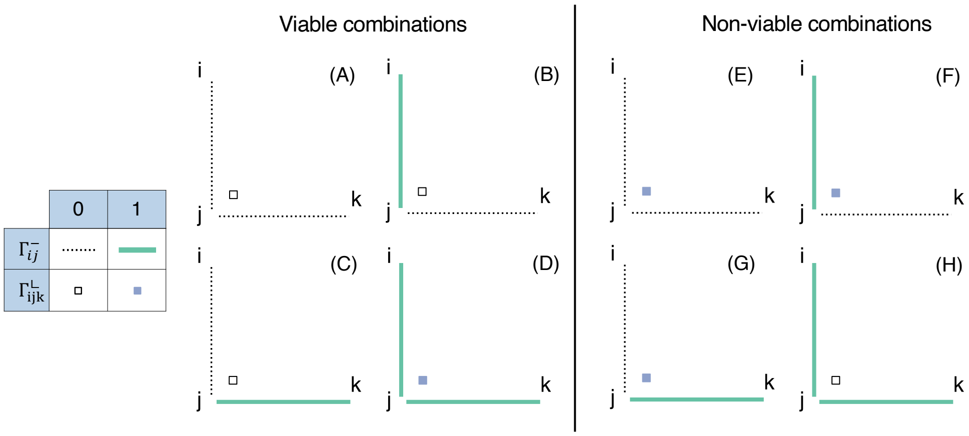

FIG. S1. Schematic representation of the possible combinations of edge and corner binary variables defined by the corner triplet ijk. Active and inactive states of the variables are represented as summarized in the graphical table. The eight possible combinations are divided into consistent (viable) and inconsistent (non-viable), which in turn are respectively mapped to ground-states and excited states of the quadratic potential V_{bc}.

3. NUMBER OF QUBITS

Data in Table S2 indicate the scaling of the number of qubits required by the different types of variables (site, bond, and corner) for cubic lattices of different size.

Lattice side	Γ_i°	Γ_{ij}^{-}, Γ_{ij}^{A}, Γ_{ij}^{B}	$\Gamma_{ijk}^{\llcorner}$
2	8	12	24
3	27	54	144
4	64	144	432
5	125	300	960
6	216	540	1800
⋮	⋮	⋮	⋮
n	n^3	$3n^2(n-1)$	$12n(n-1)^2$

TABLE S2. Number of qubits required for encoding different types of binary variables on cubic lattices of increasing side.

4. OPTIMIZATION OF THE QUBO HAMILTONIAN

A. Potential coefficients

We employed an iterative grid search method to identify the optimal values for the potential coefficients, A_{m}, A_{b}, A_{mb}, A_{c} and A_{bc} in Eq. 1, so to achieve a relatively smooth energy landscape. As a measure of optimality, we computed the success rate of finding ground states using the classical annealing protocol for space-filling melts on the $5\times5\times4$ lattice, using the minimum run time required by `neal`. The optimization involved varying each of the parameters in the set $\{\frac{1}{8}, \frac{1}{4}, \frac{1}{2}, 1, 2, 4, 8\}$. The scanning for the optimal parameters yield unitary values for all potential coefficients, except for $A_{\mathrm{curv}} = 1/2$.

B. Qubits optimization at space-filling

The general QUBO model of Eq. 2 in the main text can be simplified in specialized contexts. One such instance, is the case of space-filling melts, where the Γ_i° variables, associated with occupied/empty states of sites, are necessarily all equal to 1. In addition, all space-filling configurations on regular, e.g. hypercubic lattices, have the same (maximal) number of contacts, making the $\mathcal{H}_{\mathrm{contacts}}$ term superfluous. Accordingly the ancilla variables Γ_{ij}^{A} and Γ_{ij}^{B} can be omitted too.

Lattice side	General	Space-filling
2	68	36
3	360	198
4	1024	576
5	2210	1260
6	4068	2340
⋮	⋮	⋮
n	$n\left(25n^2-39n+12\right)$	$3n(n-1)(5n-4)$

TABLE S3. Total number of qubits required for encoding the QUBO model on cubic lattices of increasing sides. The second column refers to the number of qubits required for the general Hamiltonian $\mathcal{H}_N + \mathcal{H}_{\mathrm{curvature}} + \mathcal{H}_{\mathrm{contacts}}$. The third column refers to the optimized number of qubits for the space-filling case.

This simplification results in a drastic reduction of the number of binary variables, or qubits, as indicated in Table S3, which in turn reduces the size of the coupling matrix and field vector. Because the coupling matrix size is a key determinant of the computational cost of solving the QUBO problem, we have that, contrary to conventional sampling, which is more and more difficult with increasing density, the QUBO-based sampling is nominally best performing at space-filling.

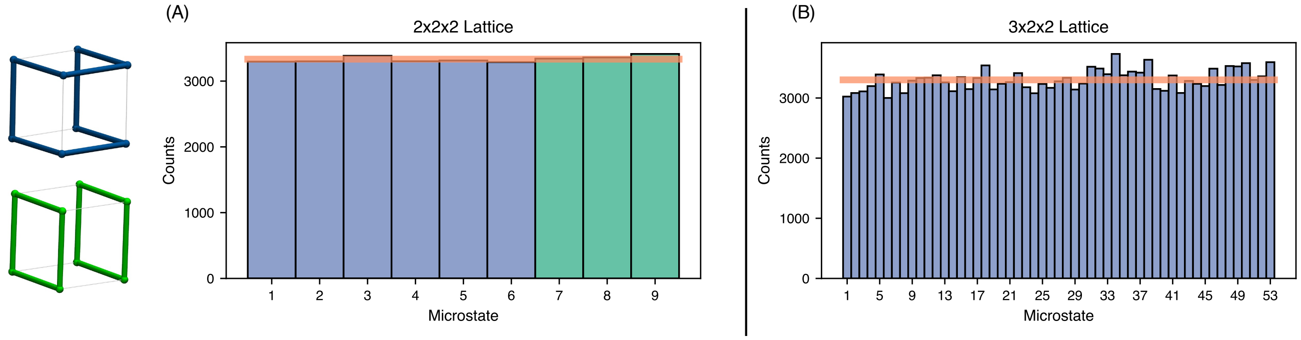

FIG. S2. Uniformity test for sampling on $2\times2\times2$ and $3\times2\times2$ lattices at complete filling using classical annealing. (A) Histogram of the occurrence of the 9 distinct microstates of a fully-filled $2 \times 2 \times 2$ lattice over $30,000$ QUBO-based samples obtained with classical annealing. The orange band corresponds to values within one standard deviation from the level expected for uniform sampling. The 9 distinct states correspond to symmetry-related (rotations) transformations of the two parent configurations on the right. The colors of the histogram bars match those of the parent configurations. (B) Histogram of the occurrence of the 53 distinct microstates of a fully-filled $3 \times 2 \times 2$ lattice over $175,000$ QUBO-based samples obtained with classical annealing.

5. UNIFORM AND ERGODIC SAMPLING

A. Classical QUBO solvers based on simulated annealing

Inspired by previous studies*(33)*, we assessed the ergodicity and uniformity of the QUBO-based sampling by considering lattices sufficiently small that all ground states of the space-filling case could be enumerated *a priori*. In such cases, the ergodicity test can be performed by: (i) collecting a number of QUBO solutions that is several-fold larger than the number of distinct ground states and (ii) verifying that they are sampled uniformly.

We carried out the analysis for $2 \times 2 \times 2$ and $3 \times 2 \times 2$ lattices, where the number of distinct space-filling configurations (no curvature restrictions) is 9 and 53, respectively, including those related by symmetry. An example is given in Fig. S2, where the two shown configurations suffice to generate, by rotations, the entire set of 9 distinct space-filling configurations on the $2 \times 2 \times 2$ lattice. Figure S2 shows that extensive resampling of these systems leads to a uniform coverage of the possible states.

We repeated the analysis for space-filling configurations on the $3 \times 2 \times 2$ lattice, but constraining the curvature by including the $\mathcal{H}_{\text{curvature}}$ potential. For a stringent test, we imposed selective curvature constraints, $n_{\text{corners}} = 8$, which reduced the number of viable configurations from 53 (free case) down to only 4. Despite the increased difficulty of dealing with much fewer global minima of the energy landscape, extensive resampling of these systems revealed uniform coverage of the 4 distinct ground states, even upon significant variations of the $\mathcal{H}_{\text{curvature}}$ coefficient, see Fig. S3.

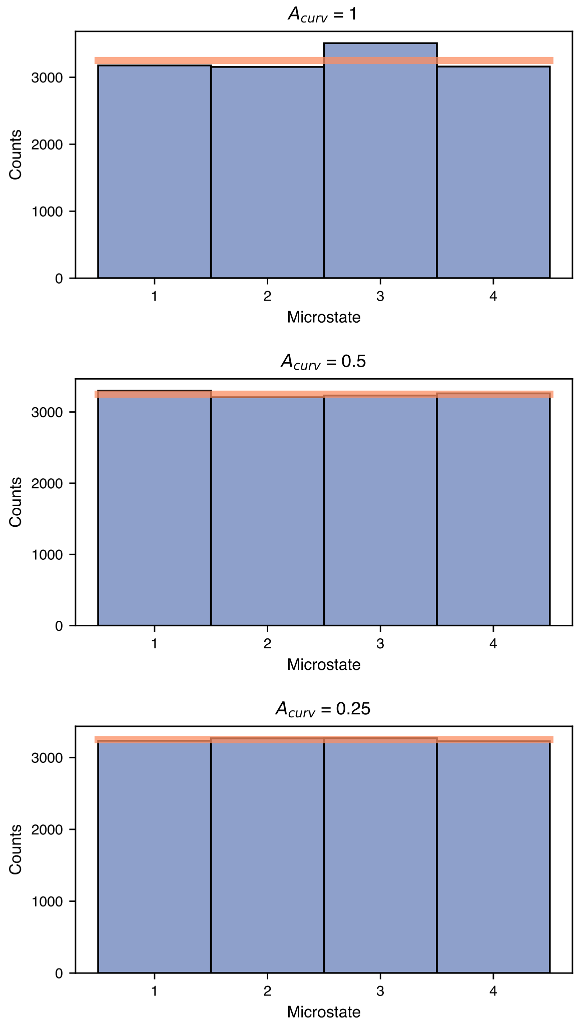

FIG. S3. Uniformity test for sampling on $3 \times 2 \times 2$ lattices at complete filling and curvature constraint using classical annealing. Histograms of the occurrence of the 4 distinct microstates of a fully-filled $3 \times 2 \times 2$ lattice with 8 corner turns over $13,000$ QUBO-based samples obtained with classical annealing for the indicated values of the A_{curv} coefficient. The orange band corresponds to values within one standard deviation from the level expected for uniform sampling.

B. Other classical QUBO solvers

We repeated the uniform coverage test for other general and powerful classical QUBO solvers not based on simulated annealing, namely Gurobi (Gurobi Optimization, LLC. Gurobi Optimizer Reference Manual, 2023) and CPLEX (IBM ILOG Cplex. V12. 1: User's manual for cplex. International Business Machines Corporation, 46(53):157,2009). The results for the $2 \times 2 \times 2$ lattice are shown in Fig. S4. Both methods cover the entire set of microstates on the ground-state manifold. However, the coverage is not uniform, arguably due to heuristics used to speed up search of low-energy solutions.

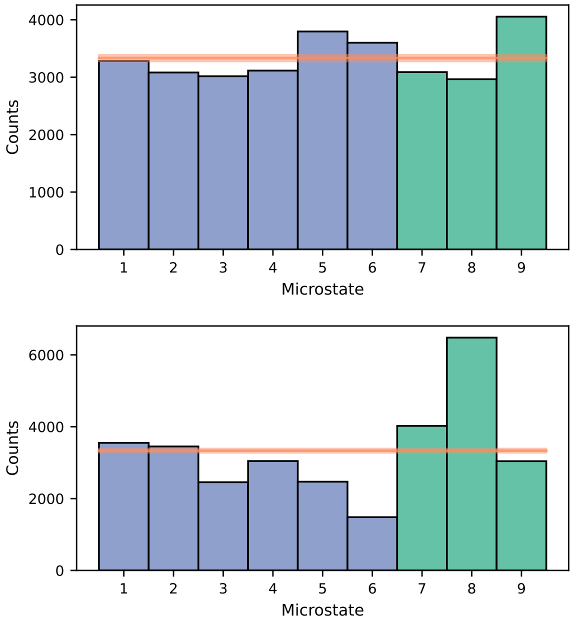

FIG. S4. Uniformity test for sampling on $2 \times 2 \times 2$ lattices at complete filling using GUROBI and CPLEX. Histogram displaying the distribution of the 9 distinct microstates of a fully-filled $2 \times 2 \times 2$ lattice, based on 30,000 samples obtained through the GUROBI solver (top) and the CPLEX solver (bottom). The orange band corresponds to values within one standard deviation from the level expected for uniform sampling. Observed deviations from both classical solvers are appreciably larger than one standard deviation.

C. Quantum QUBO solvers

Finally, we repeated the uniform sampling test for the D-wave quantum annealer. We resorted to the fully-quantum D-wave solver, as it allowed for overriding the conservative run-time lower bound hardcoded in the D-wave hybrid annealer, which is 3s for the $2 \times 2 \times 2$ systems. With the fully-quantum solver, the same problem can be solved in $\sim$ 10ms, which enabled us to gather statistics on thousands of runs, as needed to evaluate the sampling uniformity of the ground state manifold. The results are shown in the new Fig. S5 and are consistent with the uniform sampling of the $2 \times 2 \times 2$ ground state manifold.

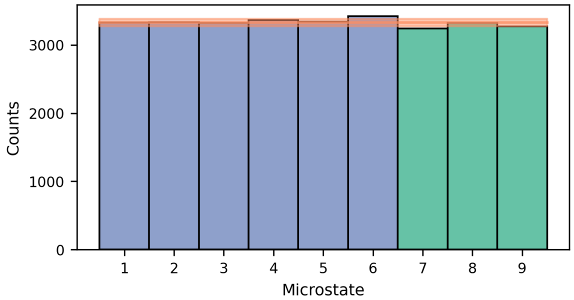

FIG. S5. Uniformity test for sampling on $2 \times 2 \times 2$ lattices at complete filling fully-quantum QUBO solvers. Histogram displaying the distribution of the 9 distinct microstates of a fully-filled $2 \times 2 \times 2$ lattice, based on 30,000 samples obtained through quantum annealing on a D-Wave machine. The orange band corresponds to values within one standard deviation from the level expected for uniform sampling.

6. AVERAGE RING LENGTH

The data collected for the main text Fig. 3 are here used to compute the average length of assembled rings as a function of N (corresponding lattice sizes are listed in Table S4), as shown in Fig. S6.

Lattice	Number of sites N
$2 \times 2 \times 2$	8
$3 \times 2 \times 2$	12
$3 \times 3 \times 2$	18
$4 \times 3 \times 3$	36
$4 \times 4 \times 3$	48
$4 \times 4 \times 4$	64
$5 \times 4 \times 4$	80
$5 \times 5 \times 4$	100
$6 \times 5 \times 5$	150
$6 \times 6 \times 5$	180
$6 \times 6 \times 6$	216
$7 \times 6 \times 6$	252
$7 \times 7 \times 6$	294

TABLE S4. List of the lattices and their corresponding size (volume) studied in this work.

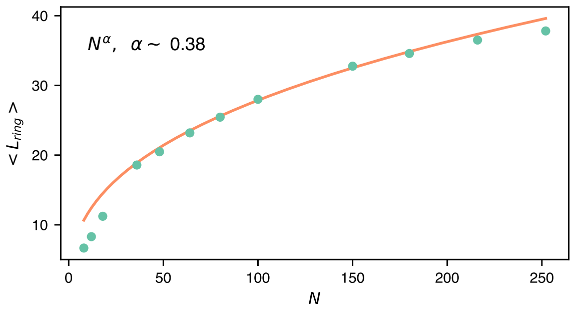

FIG. S6. Average number of monomers per ring for space-filling melts on cuboids of increasing volume, N.

7. ENTANGLEMENT PROPERTIES OF SELF-ASSEMBLED RING MELTS

A. Inter-chain entanglement, linking

In the main text, we characterized the inter-chain entanglement using the unlinking probability, i.e., the probability that all rings pairs in a microstate have Gaussian linking number equal to zero. Microstates with a single self-assembled ring were not considered. Here we complement the analysis by restricting considerations to microstates that consist of exactly two rings. For such states the the linking probability is naturally defined as the fraction of two-ring configurations that have Gaussian linking number different from zero, and its complement to 1 yields the unlinking probability.

The unlinking probability of two-ring states in shown in Fig. S7. For increasing system size, N, the unlinking probability decays approximately exponentially, as indicated in the graph and figure caption.

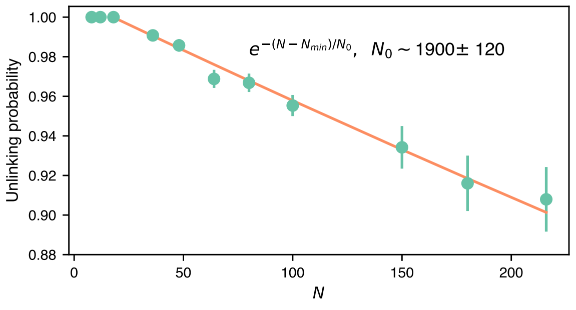

FIG. S7. Unlinking probability of two-ring microstates for fully-filled cuboids of size N. The two-ring microstates were extracted *a posteriori* from the equilibrium ensemble of space-filling ring melts, where the number of rings is not fixed. The linked state was established using the Gaussian linking number. The solid line is the best-fit to the $N \geq 36$ data with the function $\exp[(N - N_{\min})/N_0]$. The best-fit parameters are $N_{\min} \sim 18$ and $N_0 \sim 1900$.

Fig. S8 presents the same entanglement measure as a function of the total curvature of space-filling melts on the $5 \times 5 \times 4$ lattice, $N = 100$.

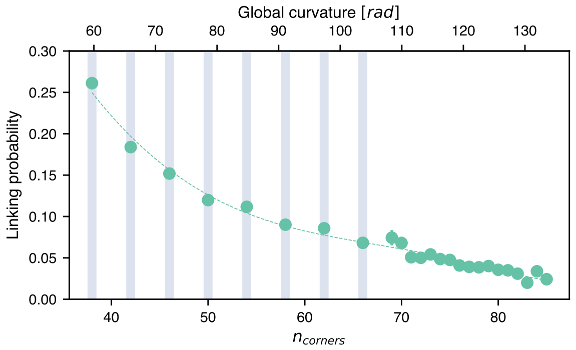

FIG. S8. Linking probability of two-ring microstates as a function of curvature for fully-filled $5 \times 5 \times 4$ cuboids, $N = 100$. The two-ring microstates were extracted *a posteriori* from the equilibrium ensemble of space-filling ring melts sampled with (blue bands) and without curvature constraints. The linked state was established using the Gaussian linking number. Data are interpolated by a spline curve.

B. Intra-chain entanglement, knotting

The same system-size- and curvature-dependent characterization can be carried out for intra-chain entanglement, i.e., knotting. To this end, we used the Alexander polynomials to determine the knotted or unknotted state of the rings in the melt. For simplicity, here we exclusively focus on the subset of space-filling microstates consisting of a single ring, i.e. Hamiltonian cycles.

We accordingly first determined the knotting probability, p_k, of such states as a function of system size, N. The results are presented in Figure S9, and show that knots in Hamiltonian cycles start to form appreciably for cuboids of size $N \sim 50$, and become more likely with increasing system size.

We next considered the incidence of knots in Hamiltonian cycles of fixed length and varying curvature, akin to varying the effective bending rigidity of the rings. The results are shown in Fig. S10. The data indicates a boost of the knotting probability with decreasing curvature, an effect that, apart from finite-size effects, is similar to that observed in off-lattice rings, where the knotting probability is maximised at a finite bending rigidity, as noted in the main text.

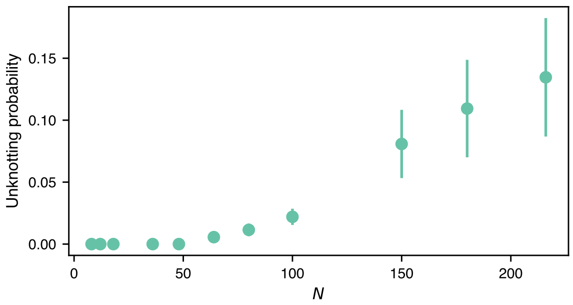

FIG. S9. Knotting probability of one-ring microstates for fully-filled cuboids of size N. The one-ring microstates were extracted *a posteriori* from the equilibrium ensemble of space-filling ring melts, where the number of rings is not fixed. The knotted state was established using the Gaussian linking number.

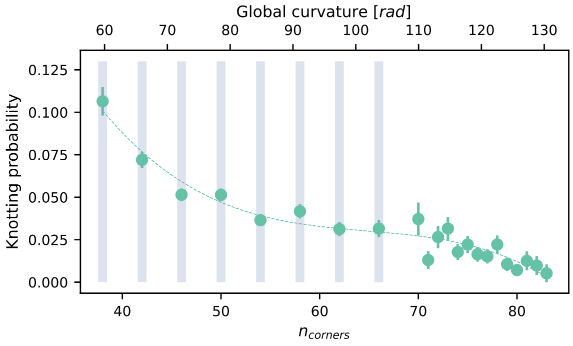

FIG. S10. Knotting probability of one-ring microstates as a function of curvature for fully-filled $5\times5\times4$ cuboids, $N = 100$. The single-ring microstates were extracted from the equilibrium ensemble of space-filling ring melts sampled with (blue bands) and without curvature constraints. The knotted state was established using the Gaussian linking number. Data are interpolated by a spline curve.

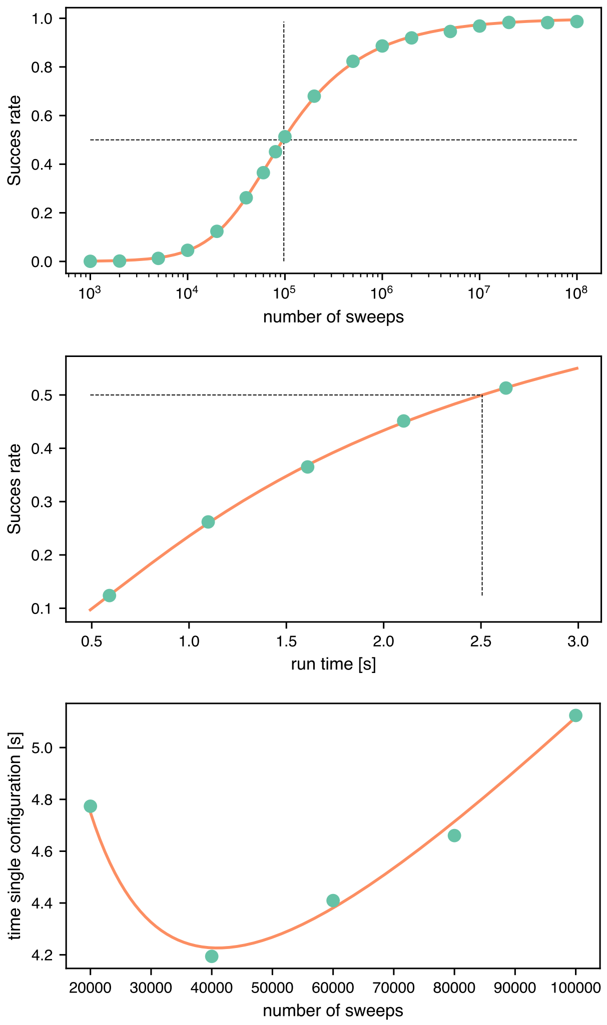

FIG. S11. Characteristic times required by classical annealing to find ground states of the QUBO Hamiltonian for ring melts on $5 \times 5 \times 4$ lattices at full filling and with curvature constraints. (top) Success rate vs number of annealing sweeps of D-Wave classical annealer for solving the QUBO model for space-filling ring melts on $5 \times 5 \times 4$ cuboids at minimal curvature. Data points are shown in green, while the orange curve is an interpolation with an asymmetrical sigmoid $s = 1 - \frac{1}{\left(1+\left(\frac{n_{sweeps}}{a}\right)^b\right)^c}$, where a, b and c are fitting parameters. The black drop line indicates the number of sweeps corresponding to 50% success rate; the associated confidence interval is estimated by the range corresponding to success rates in the 45–55% range. (center) Success rate as a function of run time. Eq. S7 yields the average run time per sample (bottom). The minimum corresponds to the optimal number of sweeps needed to maximimise the generation of samples.

8. PERFORMANCE OF CLASSICAL AND HYBRID ANNEALERS

For an equal footing comparison of the performance of classical and hybrid D-Wave QUBO solvers, we considered the $\tau_{1/2}$ parameter, which is the annealing time yielding a 50 % success rate per run of finding a solution to a given QUBO problem. For the comparison, we considered space-filling ring melts on cuboids with number of sites N ranging from 8 to 252, using default settings for the classical and hybrid solvers.

The solvers' success rate, the probability of finding a correct solution per trial, was computed for increasing number of the annealing sweeps. For each considered number of sweeps we used at least 7 runs for the hybrid solvers. Because of the wider availability of classical resources on conventional high-performance computing

clusters, the number of minimum runs was increased to 100 for the classic D-Wave solver. The sought $\tau_{1/2}$ value was obtained by first identifying the number of sweeps yielding a 50% success rate, and then measuring the associated CPU time. The 50% success rate was determined from the best fit of the success rate curves using the characteristic functional forms of classical and hybrid annealers described in the captions of Fig. S11, Fig. S11 and S12, which present typical curves. The reported times are for runs on the D-Wave IDE platform. At the largest system sizes, the relevant run times exceeded the D-Wave IDE time limits. To circumvent this practical limit, we used the classical `neal` solver on Intel-based workstations and converted the number of annealing cycles to the sought D-Wave-IDE equivalent one. This conversion harnessed the local linear dependence of `neal` annealing cycles and annealing times, with the proportionality coefficients established for each QUBO problem using D-Wave IDE runs of duration not exceeding the time limit. For the statistical uncertainty of $\tau_{1/2}$, we conservatively took the time interval corresponding to success rates in the 0.45-0.55 range in the classical case, and in the 0.2-0.8 range in the quantum one.

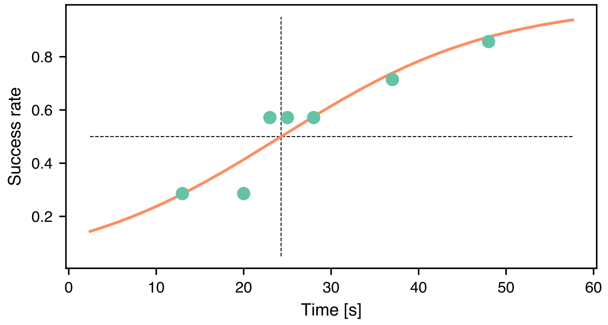

FIG. S12. Success rate vs run time of D-Wave hybrid annealer for solving the QUBO model for space-filling ring melts on $6 \times 6 \times 6$ cuboids. Data points are shown in green, while the orange curve is an interpolation with the functional form $s = \left(1 + \tanh\left(\tau - \tau_{1/2}\right)/\delta\right)$, where τ is the run time, and $\tau_{1/2}$ and δ are fitting parameters. The 50% success rate defines $\tau_{1/2}$ (black dashed line), whose confidence interval is estimated by the range corresponding to success rates in the conservative 20–80% range.

A. Optimal sampling

The optimal production rate of solutions with classical annealers can be achieved for a number of sweeps different from the one corresponding to $\tau_{1/2}$. The average time required to return one QUBO solution, i.e. a valid polymer conformation, t_{conf}, for a given number of sweeps, n_{sweeps} is given by:

$$t_{\mathrm{conf}} = \frac{t\left(n_{\mathrm{sweeps}}\right)}{s\left(n_{\mathrm{sweeps}}\right)} , \qquad \text{(S7)}$$

where $t\left(n_{\mathrm{sweeps}}\right)$ is the average time per required for the sweeps and $s\left(n_{\mathrm{sweeps}}\right)$ is the success rate, i.e. the aforementioned probability of finding a correct solution per trial. The optimal productivity is achieved for the number of sweeps that minimized t_{conf}. A typical curve for t_{conf} versus n_{sweeps} is shown in Fig. S11, where the optimal production is achieved for $n_{\mathrm{sweeps}} \sim 40,000$. This optimal production rate was obtained with a custom simulated annealing schedule, namely a linear schedule between inverse temperatures $\beta = 1$ and $\beta = 10$. This custom schedule was applied for all considered cases and system sizes, N. For optimal productivity in the minimal-curvature case, the potential coefficients A_{b} and A_{curv} were modified with respect to their default values and set equal to $A_{\mathrm{b}} = 2$ and $A_{\mathrm{curv}} = 1$.

9. SAMPLING WITH REPLICA EXCHANGE METHOD

For comparative purposes, we implemented a replica exchange method to sample ring-melt conformations in real space. The sampling was performed for cuboids at complete filling and with the minimum curvature constraint. The condition of complete filling allows for evolving the system with a particularly efficient set of moves, which we term plaquette-flip moves. These moves, which are inspired by refs. *(33, 65, 66)* are sketched in Fig. S13, where the square lattice was used for clarity. The moves are applicable to plaquettes with two *parallel* bonds (shown in yellow in the shown example) and consist of switching the occupied/empty state of the two sets of parallel bonds. The moves preserve the number of bonds in the system while allowing the chain connectivity, the number of rings, and number of corners to fluctuate. The moves thus allow for exploring the same conformational space sampled with our QUBO models with unrestricted curvature, with the proviso of initializing the replica exchange system to a state with the correct number of bonds.
We note that the plaquette moves are ergodic on cubic lattices at complete filling, though not so at smaller filling fractions, as shown in Figure S14. In addition, the moves are generally not applicable to arbitrary regular or irregular lattices.

We used the plaquette-flip moves in a replica-exchange scheme. At each time step, from 1 to 10 plaquettes are picked at random from the lattice, and those that are involved two parallel occupied edges (bonds) and two parallel empty edges are flipped. The obtained configuration is accepted or rejected based on the Metropolis criterion, the energy of a configuration being equal to the total curvature, i.e. the number of corners. The number of replicas and their temperatures were optimized to ensure a broad coverage of curvature values. As few as eight replicas sufficed to the purpose at all values of N, including a replica at almost zero temperature, which served as collector for the lowest-energy solutions. To ensure suffi-

ciently frequent proposed exchanges, the swap time was set (based on preconditioning runs) to a value at least 100 times smaller than the autocorrelation time of the lowest temperature replica, $\bar{\tau}$.

The autocorrelation time, $\bar{\tau}$, was computed based on the decay of the time-lagged average overlap, $\langle q(t, t+\tau)\rangle$, where the average runs over simulation time, t, and $q(t, t+\tau)$ is the overlap of the system configurations in the replica of interest at time t and $t+\tau$, that is the fraction of bonds that occupy the same lattice edges. The raw $\langle q(t, t+\tau)\rangle$ curve was used to define the regularized autocorrelation function $C(\tau) = (\langle q(t, t+\tau)\rangle - q_\infty)/(1-q_\infty)$, where q_∞ is the limiting value of the overlap at large lag times. The autocorrelation time, $\bar{\tau}$, was finally computed by numerically integrating $C(\tau)$ up to the smallest lag time for which it falls below 0.01.

The characteristic CPU time required per uncorrelated solution was calculated as:

$$t = \frac{t_{\rm tot}}{n_{\rm sol}/\bar{\tau}} \ , \qquad \text{(S8)}$$

where $t_{\rm tot}$ is the total CPU time, $n_{\rm sol}$ is the number of solutions (minimum curvature states) collected in the lowest-temperature replica, and $\bar{\tau}$ is the autocorrelation time of the lowest-temperature replica.

The source code of the replica exchange method and the setup files for the various considered systems are available as supporting material and also at: `github.com/CristianMicheletti/REM_ring_melts/` .

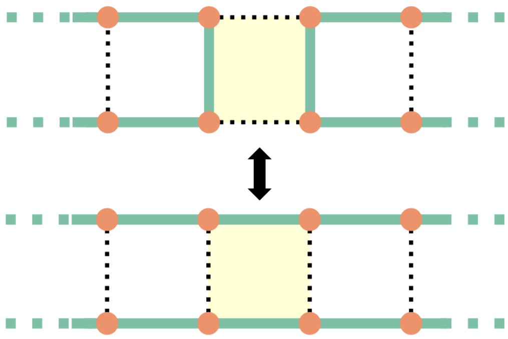

FIG. S13. Plaquette-flip moves used in the replica-exchange simulation. The move is applicable to plaquettes with two parallel occupied edges (bonds) and two parallel unoccupied ones, as for the highlighted plaquette (yellow). The move consists of swapping the uccupied and unoccupied edges of the plaquette.

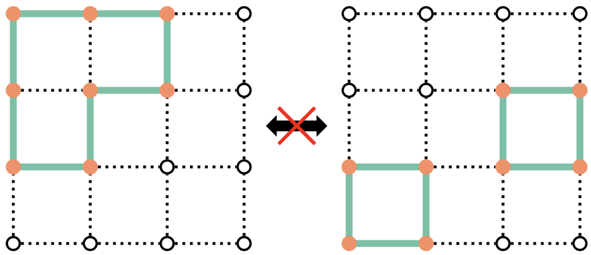

FIG. S14. Example of two configurations that, despite having same length, cannot be evolved one into the other using plaquette-flip moves.

DATA FILE S1

Data ZIP file containing data files related to figures in main text and supplementary material, source codes, set up files and analysis scripts.
