# Supplementary material for: Quantum-inspired encoding enhances stochastic sampling of soft matter systems
Source: Sci Adv. 2023 Oct 25;9(43):eadi0204. doi: 10.1126/sciadv.adi0204 (PMC10599611; doi:10.1126/sciadv.adi0204)
Supplement: Supplementary file 2 — Data file S1 [file sciadv.adi0204_data_file_s1.zip › Data_related_to_Main_text_figures/Figure_7/README.rtf]

Folder with the data relevant for Figure 7 of the main text.The folder “Python_driver_for_neal_solver_and_sample_input” contains the files needed for obtaining the samples through the “Default_Annealer.py”  and “Default_Annealer.py” script.The “Coefficients.txt” contains the proportionality coefficients between the annealing steps and the minimisation times. The files “Classical_t12_plot_points.dat”, “Classical_topt_plot_points.dat” and “Hybrids_plot_points” contain the numerical values of the data points in the plot. 
