# Supplementary material for: Quantum-inspired encoding enhances stochastic sampling of soft matter systems
Source: Sci Adv. 2023 Oct 25;9(43):eadi0204. doi: 10.1126/sciadv.adi0204 (PMC10599611; doi:10.1126/sciadv.adi0204)
Supplement: Supplementary file 2 — Data file S1 [file sciadv.adi0204_data_file_s1.zip › Data_related_to_Main_text_figures/Figure_4/README.rtf]

Folder with the data relevant for Figure 4 of the main text.The folder “Python_driver_for_neal_solver_and_sample_input” contains the files needed for obtaining the samples through the “Annealer.py” script. The data were obtained with the dwave-neal library, version 0.5.9, build pypi_0, channel pypi.  The .dat files contain the numerical values of the data used for the plot.
