# Supplementary material for: Quantum-inspired encoding enhances stochastic sampling of soft matter systems
Source: Sci Adv. 2023 Oct 25;9(43):eadi0204. doi: 10.1126/sciadv.adi0204 (PMC10599611; doi:10.1126/sciadv.adi0204)
Supplement: Supplementary file 2 — Data file S1 [file sciadv.adi0204_data_file_s1.zip › Visualization_Code/README.rtf]

To pass from the spin string to the polymer configuration, we provide here the visualization script called “Lattice_plot.py” with some example configurations. In order to run the script, write in a terminal:“python Lattice_Plot.py XXX YYY”, where XXX is the name of the file with the states and YYY is the name of the file called INPUT_PARAMS.DAT of the corresponding states.To visualise the 2 examples, paste in the terminal:python Lattice_Plot.py Examples/2x2x2/state.txt Examples/2x2x2/INPUT_PARAMS.DATpython Lattice_Plot.py Examples/5x5x4/state.txt Examples/5x5x4/INPUT_PARAMS.DAT
