# Supplementary figures and images for: Quantum-inspired encoding enhances stochastic sampling of soft matter systems
Source: Sci Adv. 2023 Oct 25;9(43):eadi0204. doi: 10.1126/sciadv.adi0204 (PMC10599611; doi:10.1126/sciadv.adi0204)

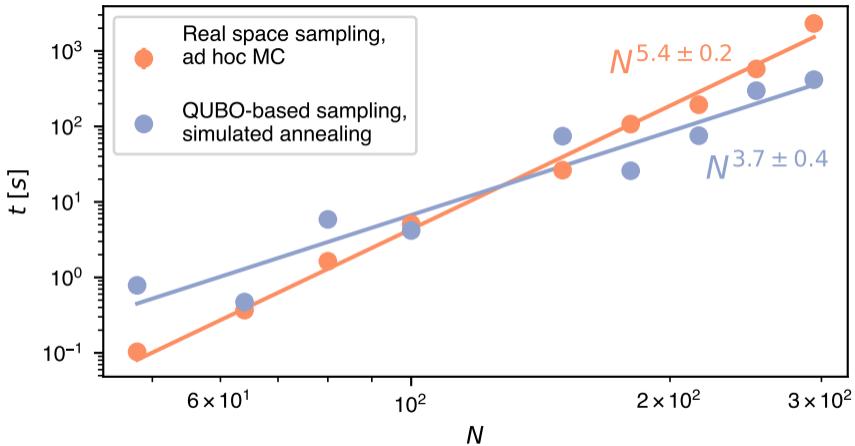

Supplement: Supplementary file 2 — Data file S1 [file sciadv.adi0204_data_file_s1.zip › Data_related_to_Main_text_figures/Figure_6/Figure_6.pdf]

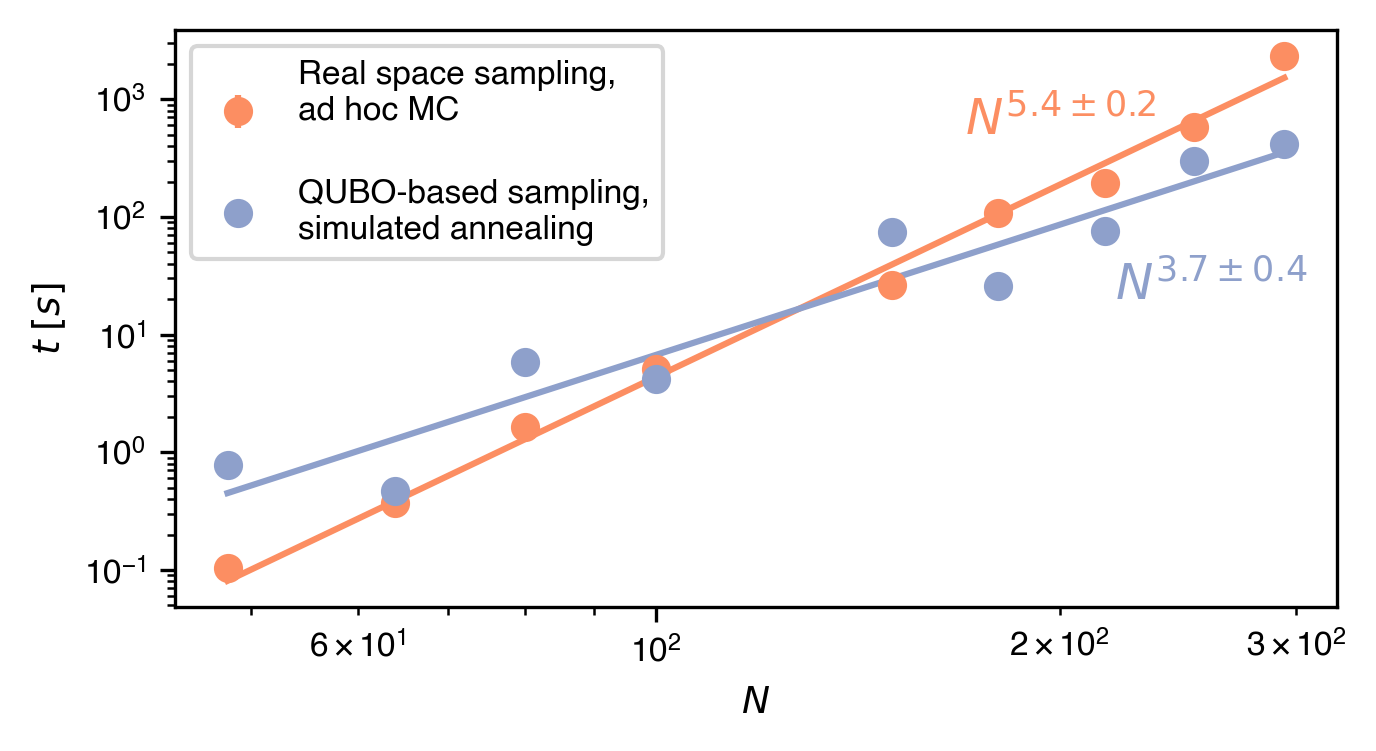

Supplement: Supplementary file 2 — Data file S1 [file sciadv.adi0204_data_file_s1.zip › Data_related_to_Main_text_figures/Figure_6/Figure_6.png]

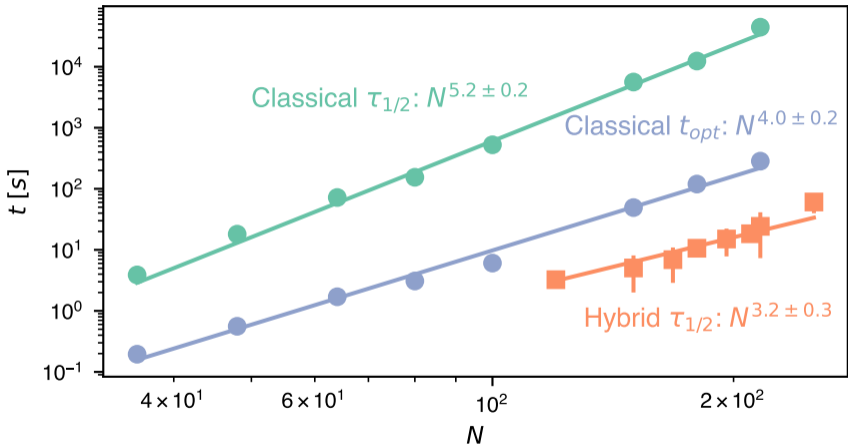

Supplement: Supplementary file 2 — Data file S1 [file sciadv.adi0204_data_file_s1.zip › Data_related_to_Main_text_figures/Figure_7/Figure_7.pdf]

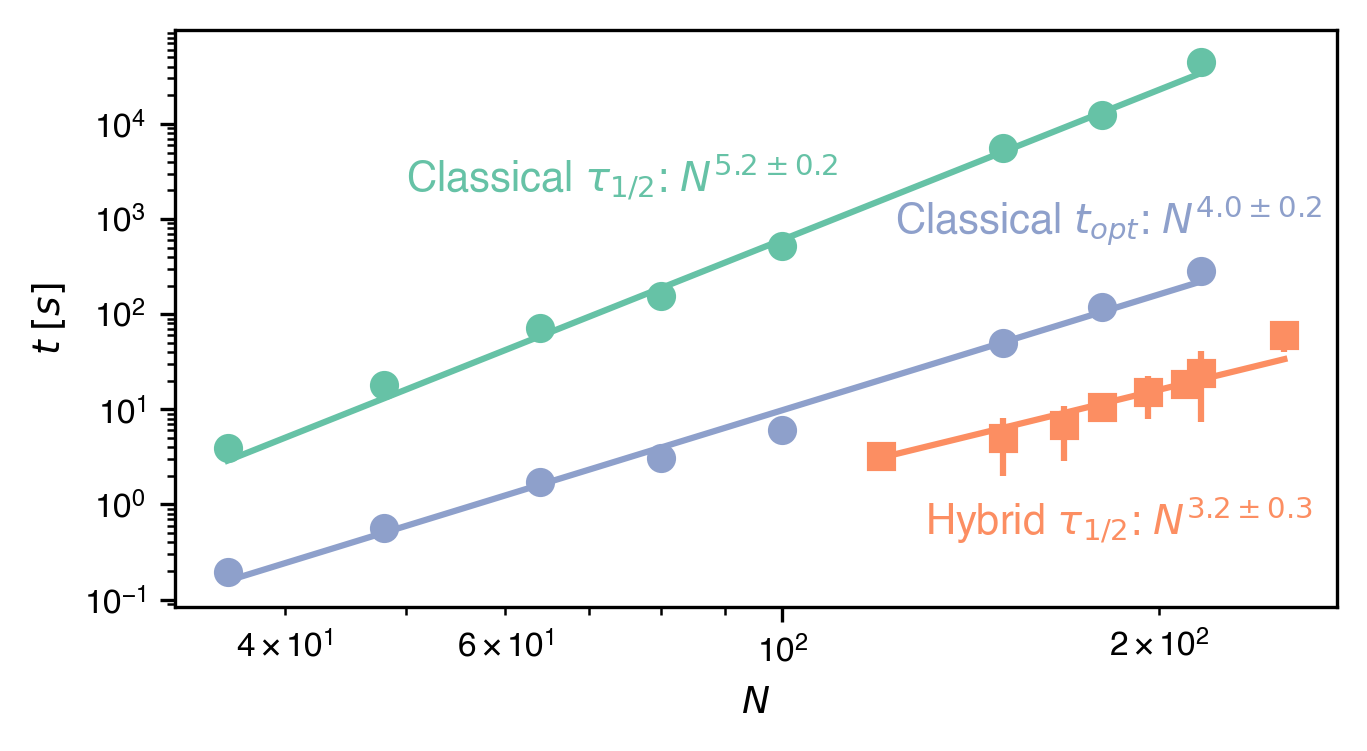

Supplement: Supplementary file 2 — Data file S1 [file sciadv.adi0204_data_file_s1.zip › Data_related_to_Main_text_figures/Figure_7/Figure_7.png]

**(A)**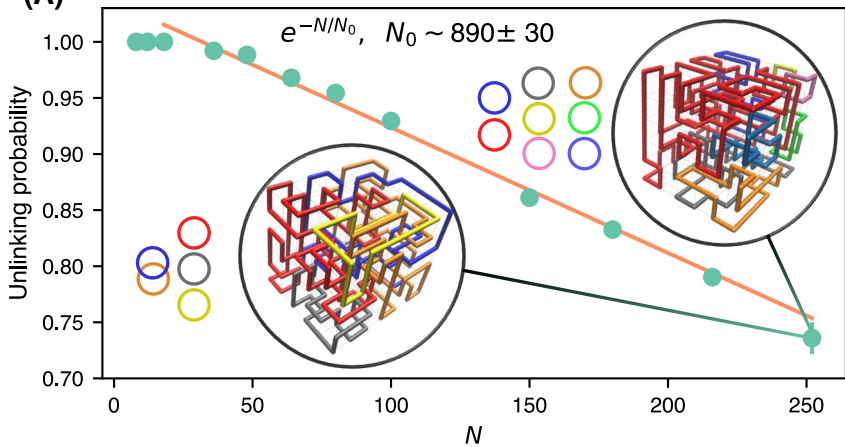**(B)**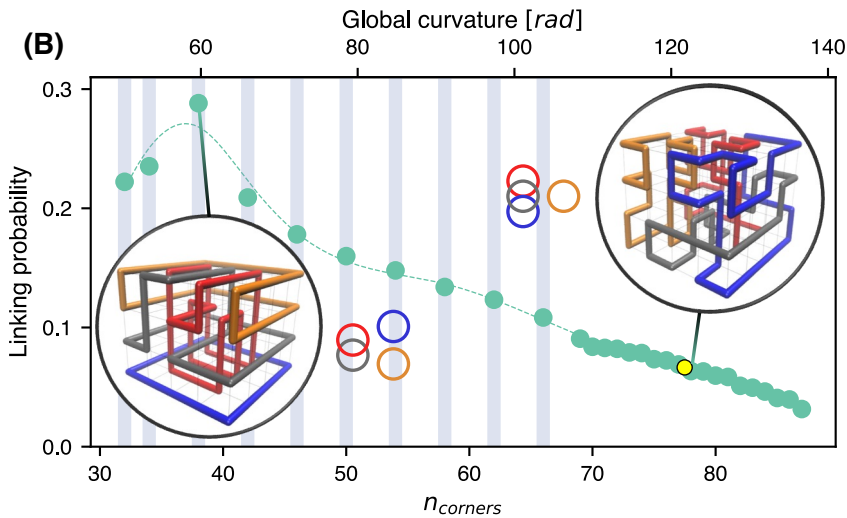

Supplement: Supplementary file 2 — Data file S1 [file sciadv.adi0204_data_file_s1.zip › Data_related_to_Main_text_figures/Figure_5/Figure_5.pdf]

## Slide 1
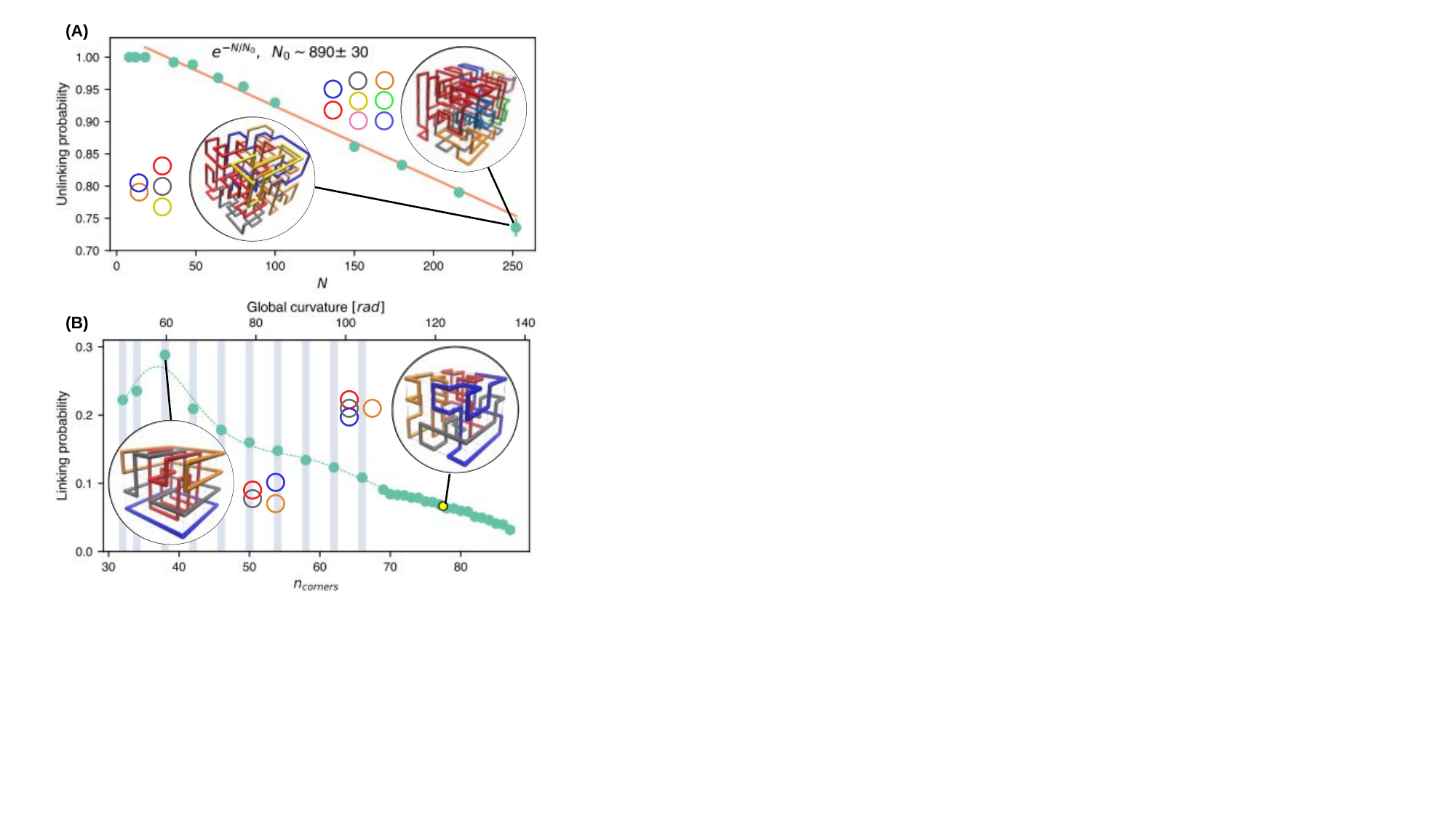

(A)
(B)

Supplement: Supplementary file 2 — Data file S1 [file sciadv.adi0204_data_file_s1.zip › Data_related_to_Main_text_figures/Figure_5/Figure_5.pptx]

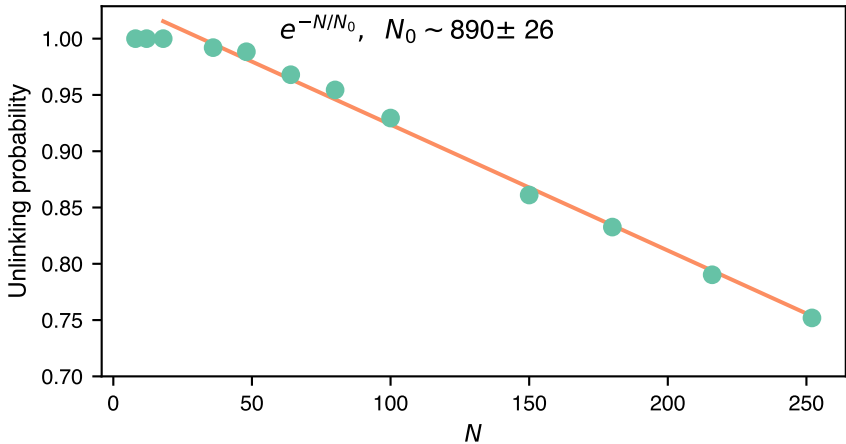

Supplement: Supplementary file 2 — Data file S1 [file sciadv.adi0204_data_file_s1.zip › Data_related_to_Main_text_figures/Figure_5/Unlinking_probability_at_least_2_rings.pdf]

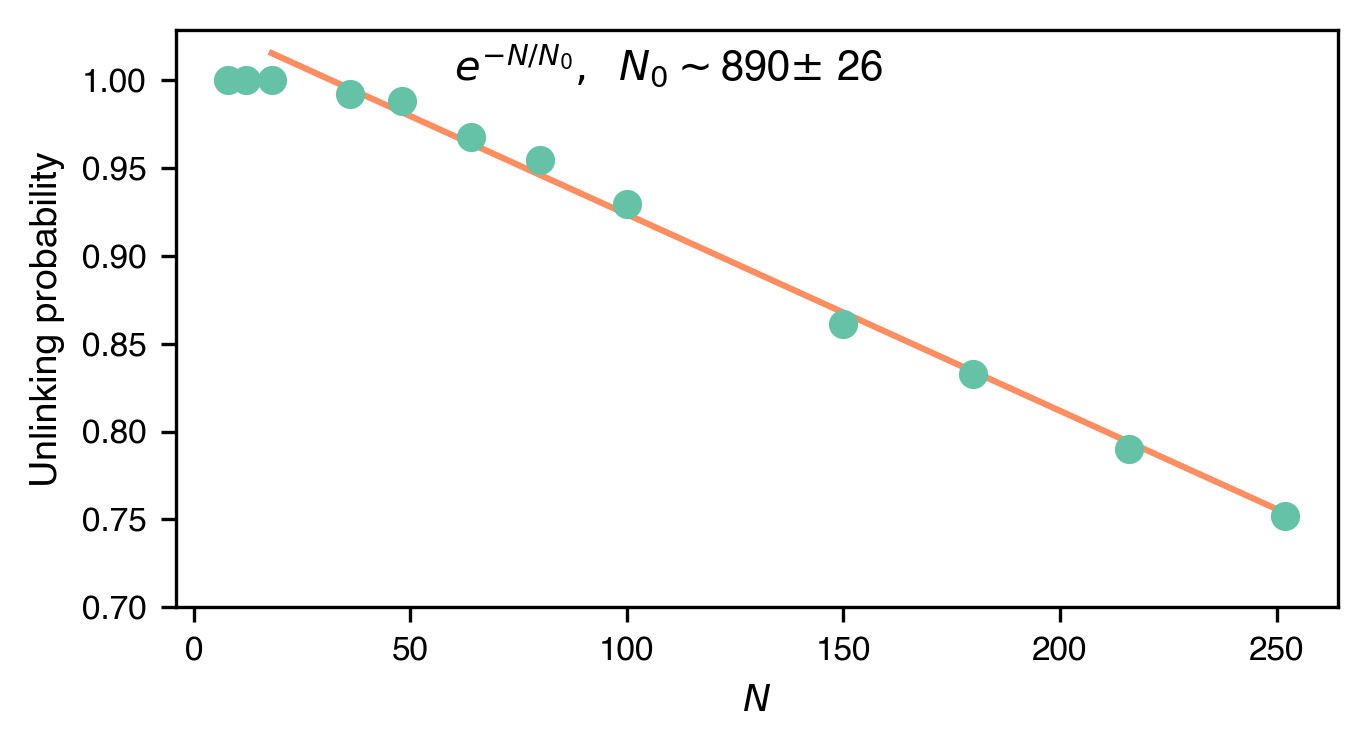

Supplement: Supplementary file 2 — Data file S1 [file sciadv.adi0204_data_file_s1.zip › Data_related_to_Main_text_figures/Figure_5/Unlinking_probability_at_least_2_rings.png]

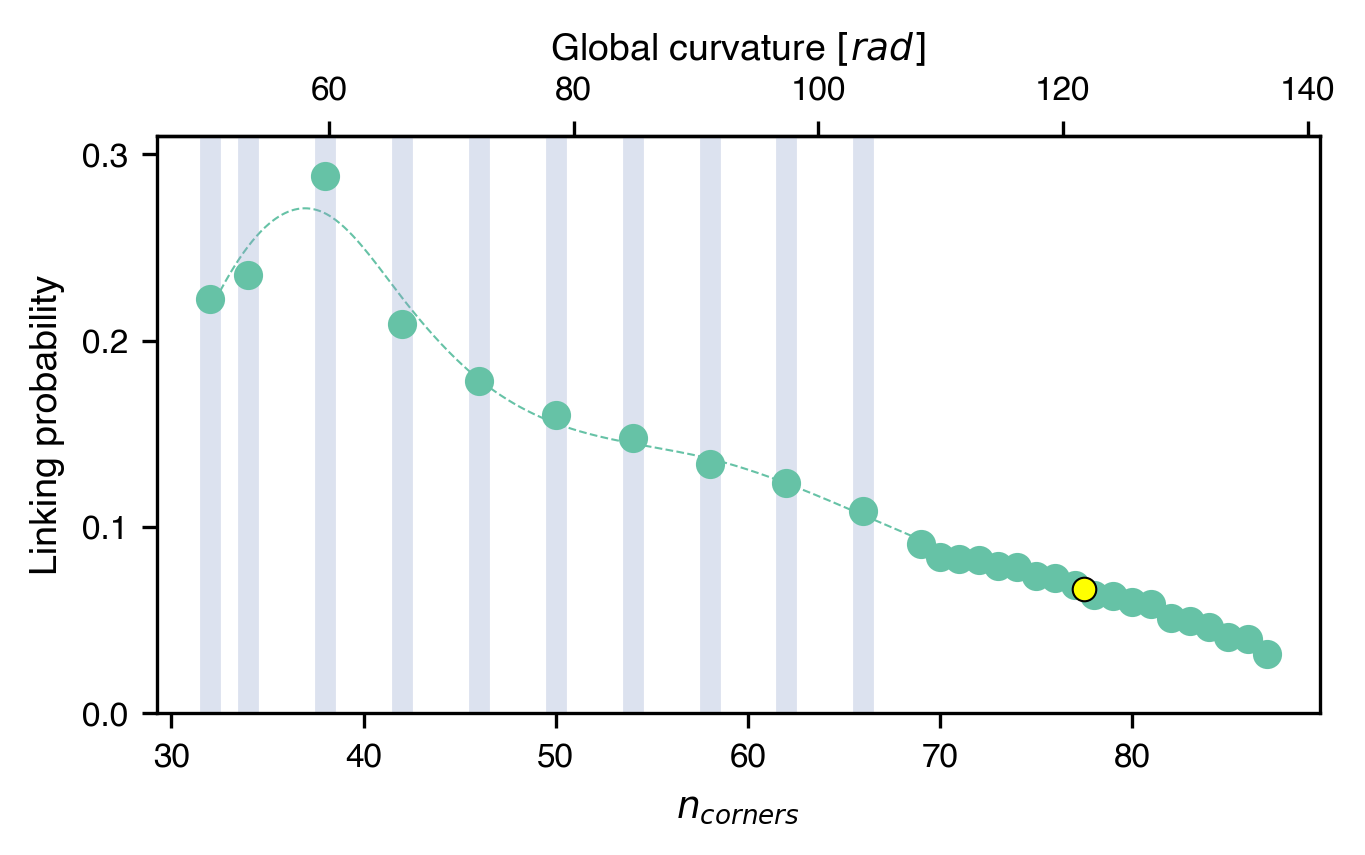

Supplement: Supplementary file 2 — Data file S1 [file sciadv.adi0204_data_file_s1.zip › Data_related_to_Main_text_figures/Figure_5/Linking_probability_vs_nc_at_least_2_rings.png]

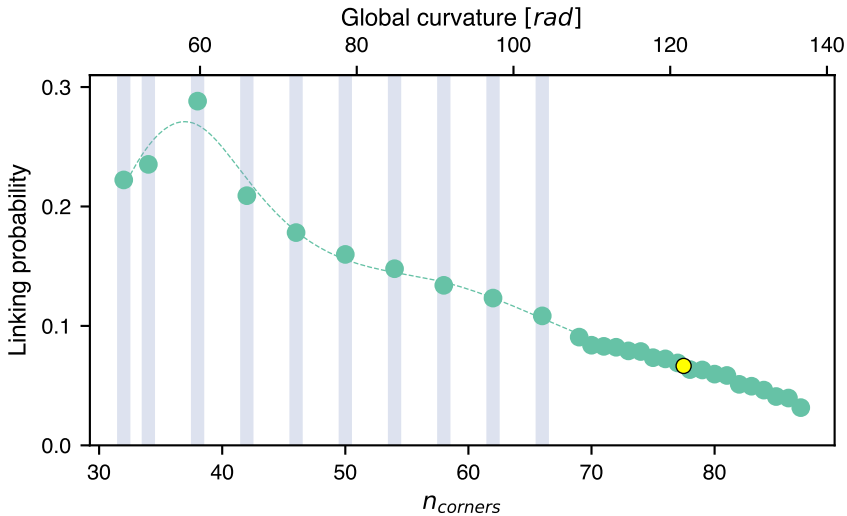

Supplement: Supplementary file 2 — Data file S1 [file sciadv.adi0204_data_file_s1.zip › Data_related_to_Main_text_figures/Figure_5/Linking_probability_vs_nc_at_least_2_rings.pdf]

## Slide 1
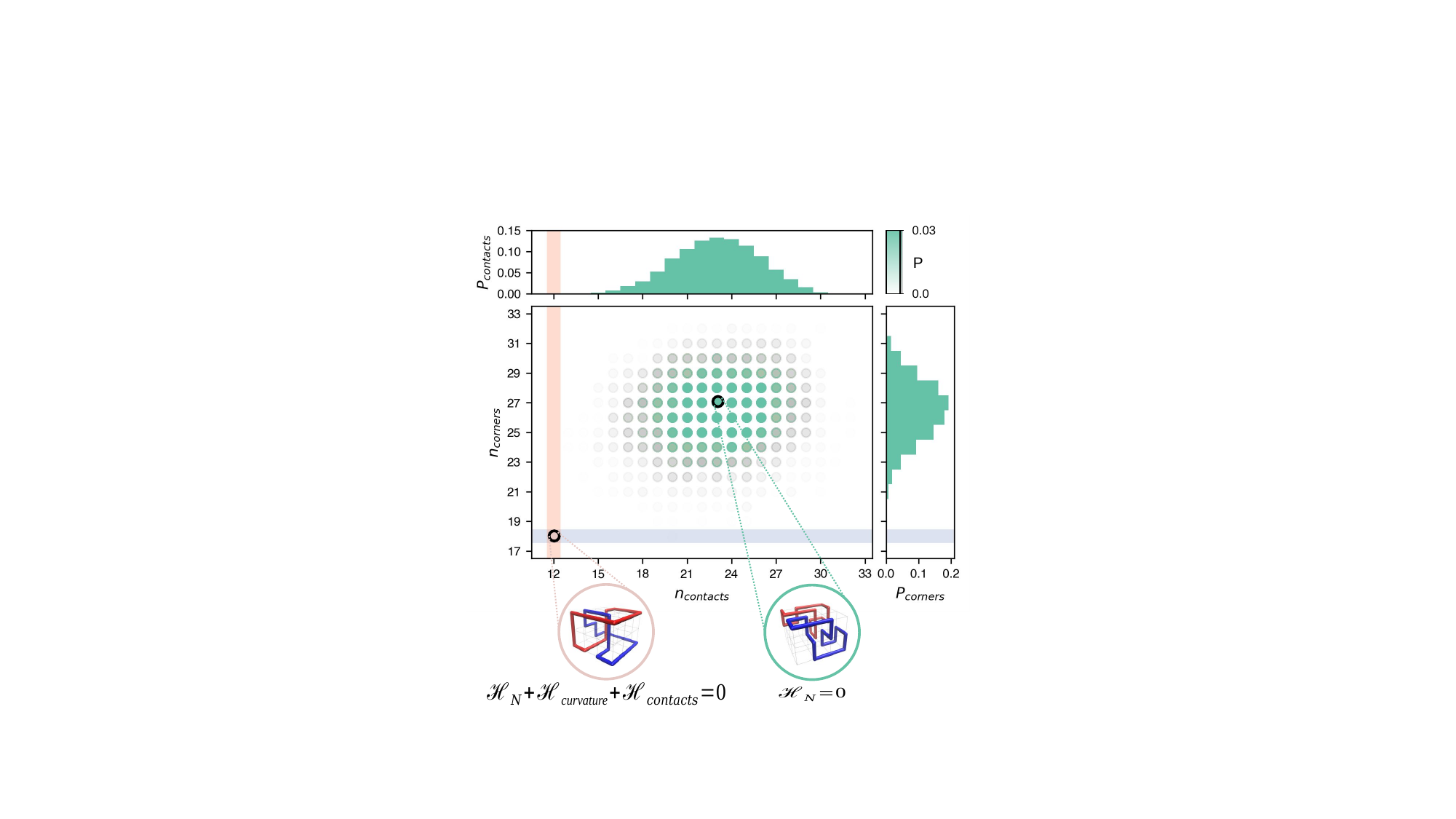

0.03
P
0.0

Supplement: Supplementary file 2 — Data file S1 [file sciadv.adi0204_data_file_s1.zip › Data_related_to_Main_text_figures/Figure_2/Figure_2.pptx]

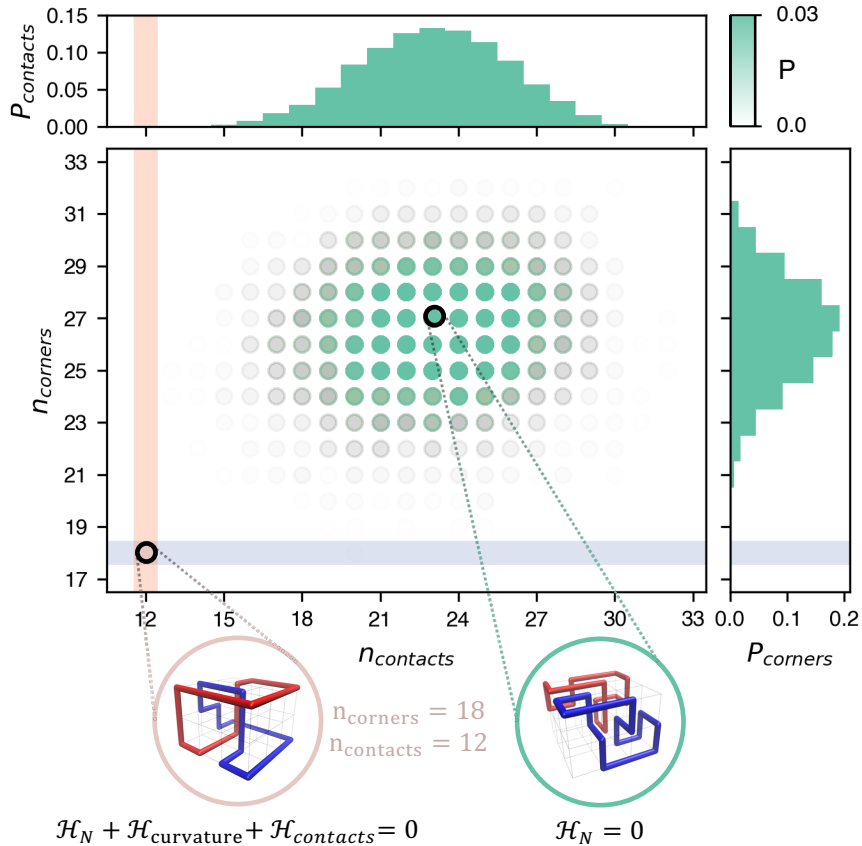

Supplement: Supplementary file 2 — Data file S1 [file sciadv.adi0204_data_file_s1.zip › Data_related_to_Main_text_figures/Figure_2/Figure_2.pdf]

## Slide 1
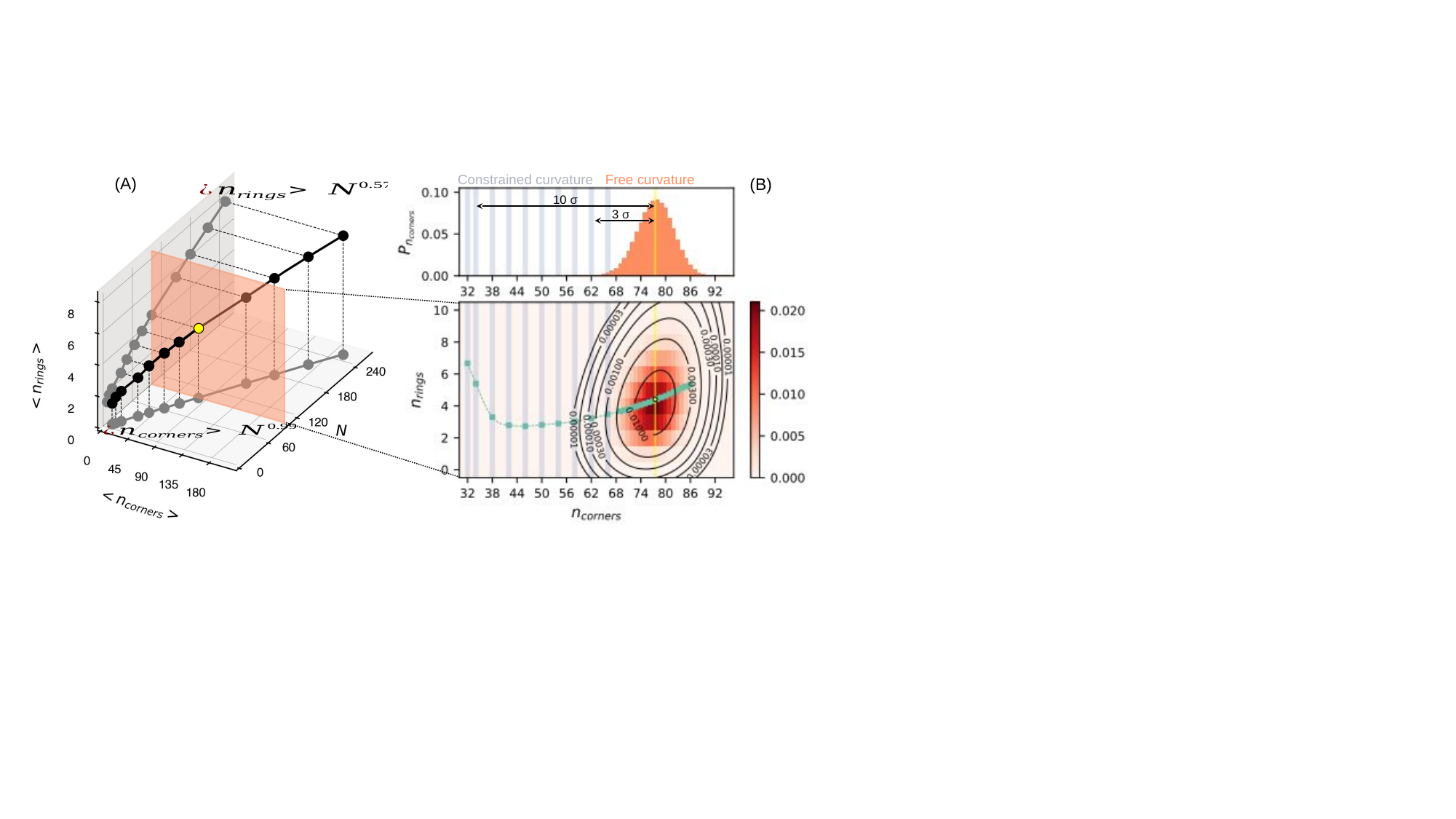

Constrained curvature
Free curvature
(A)
(B)
10 σ
3 σ

Supplement: Supplementary file 2 — Data file S1 [file sciadv.adi0204_data_file_s1.zip › Data_related_to_Main_text_figures/Figure_3/Figure_3.pptx]

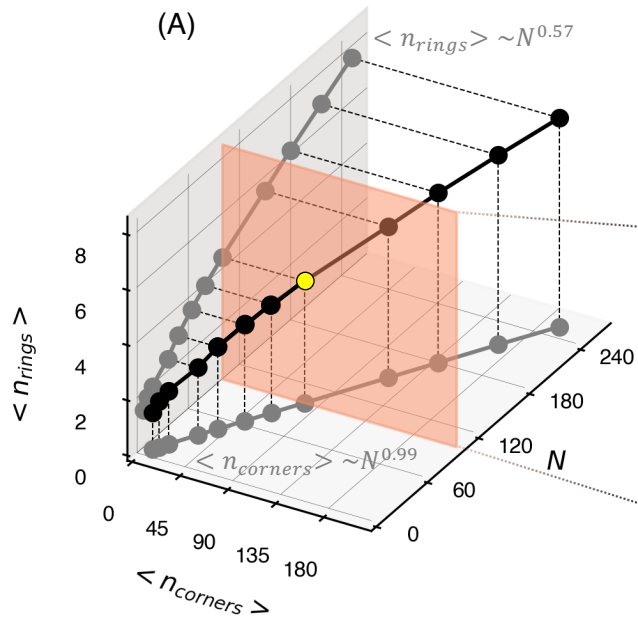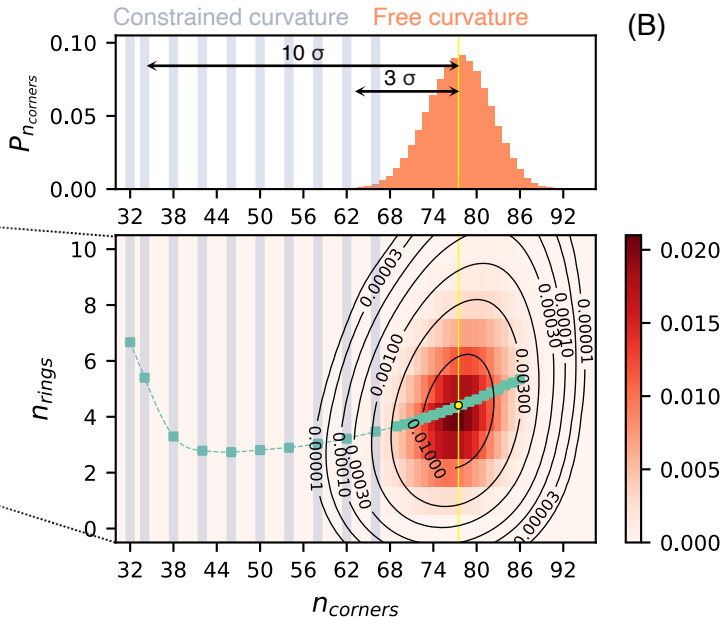

Supplement: Supplementary file 2 — Data file S1 [file sciadv.adi0204_data_file_s1.zip › Data_related_to_Main_text_figures/Figure_3/Figure_3.pdf]

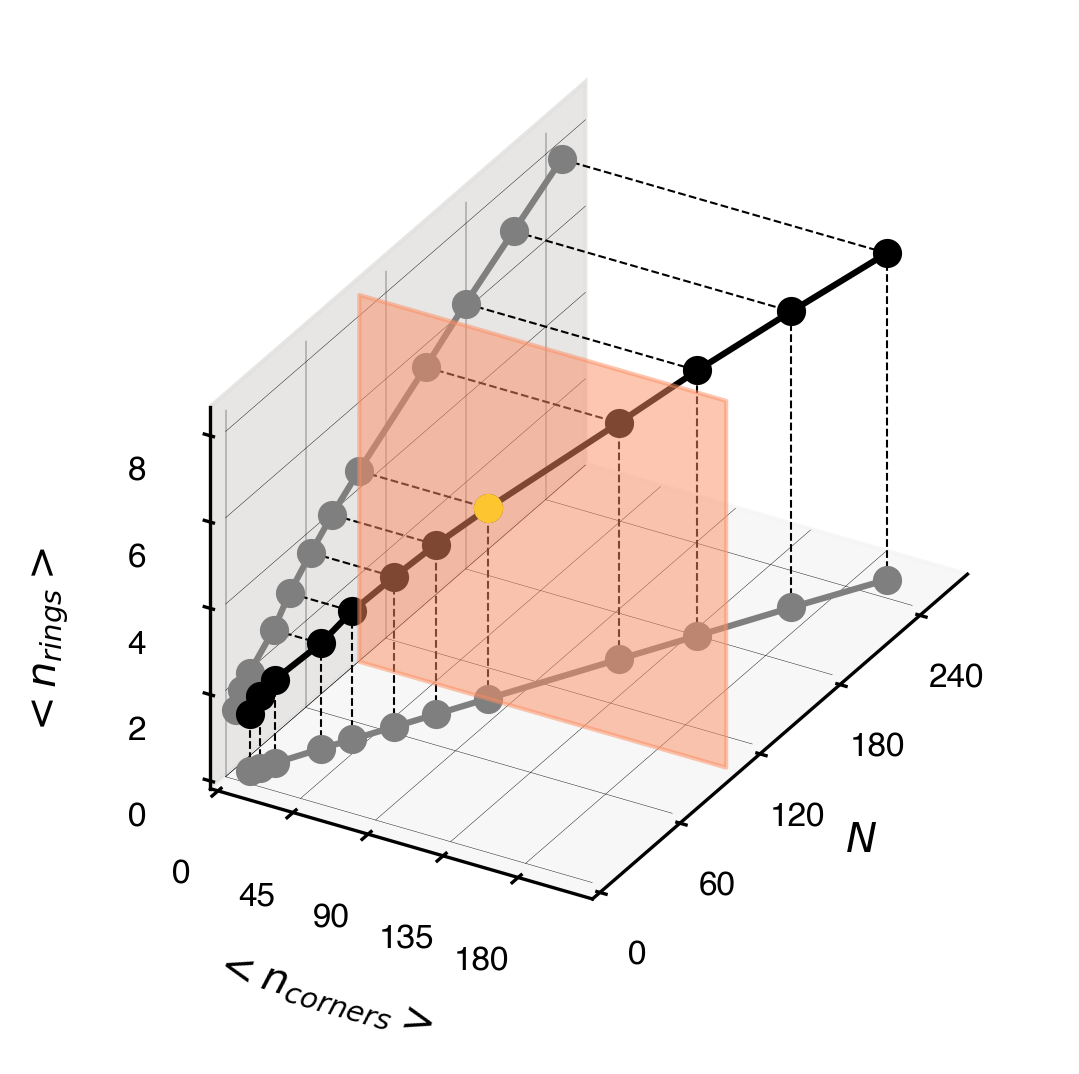

Supplement: Supplementary file 2 — Data file S1 [file sciadv.adi0204_data_file_s1.zip › Data_related_to_Main_text_figures/Figure_3/Nrings_vs_nc_3D.png]

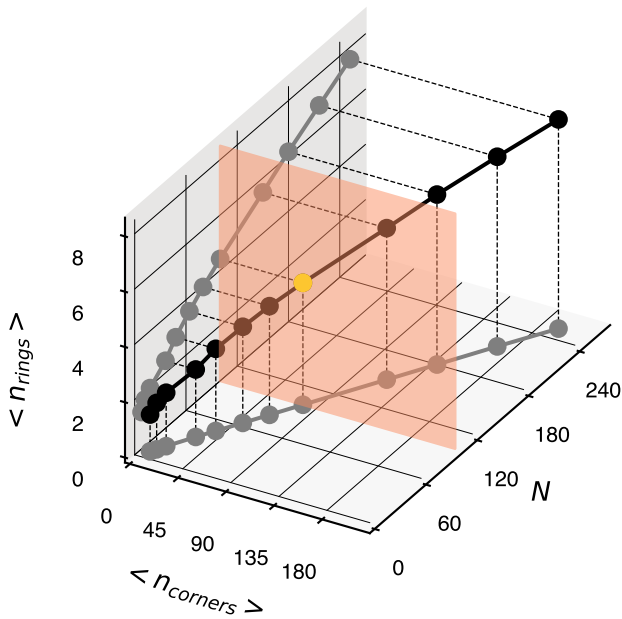

Supplement: Supplementary file 2 — Data file S1 [file sciadv.adi0204_data_file_s1.zip › Data_related_to_Main_text_figures/Figure_3/Nrings_vs_nc_3D.pdf]

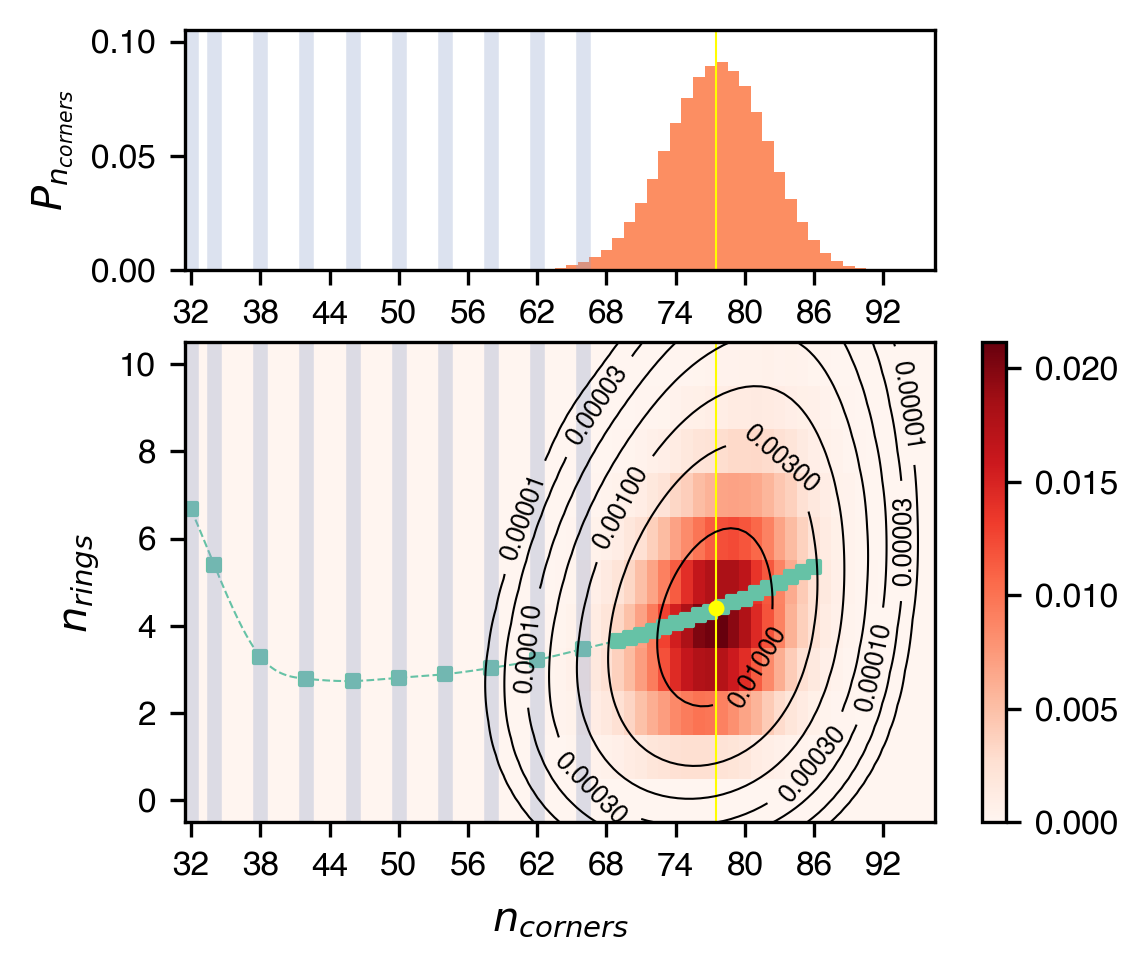

Supplement: Supplementary file 2 — Data file S1 [file sciadv.adi0204_data_file_s1.zip › Data_related_to_Main_text_figures/Figure_3/5x5x4_2D_histogram.png]

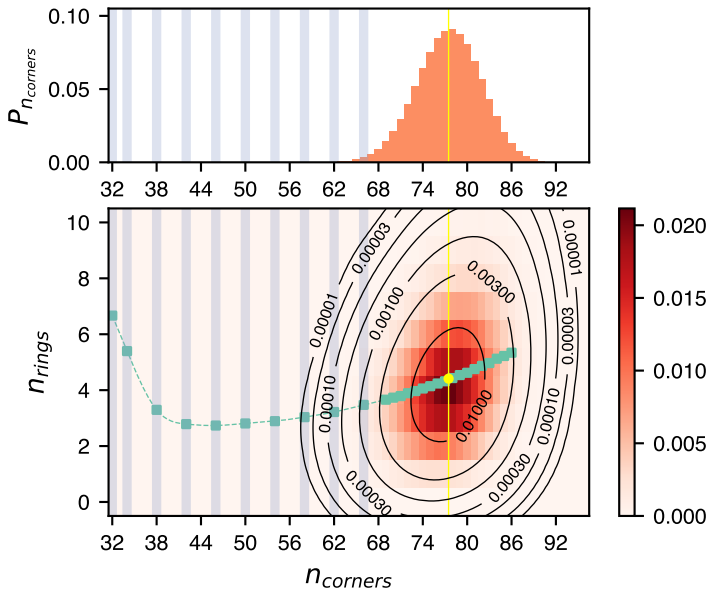

Supplement: Supplementary file 2 — Data file S1 [file sciadv.adi0204_data_file_s1.zip › Data_related_to_Main_text_figures/Figure_3/5x5x4_2D_histogram.pdf]

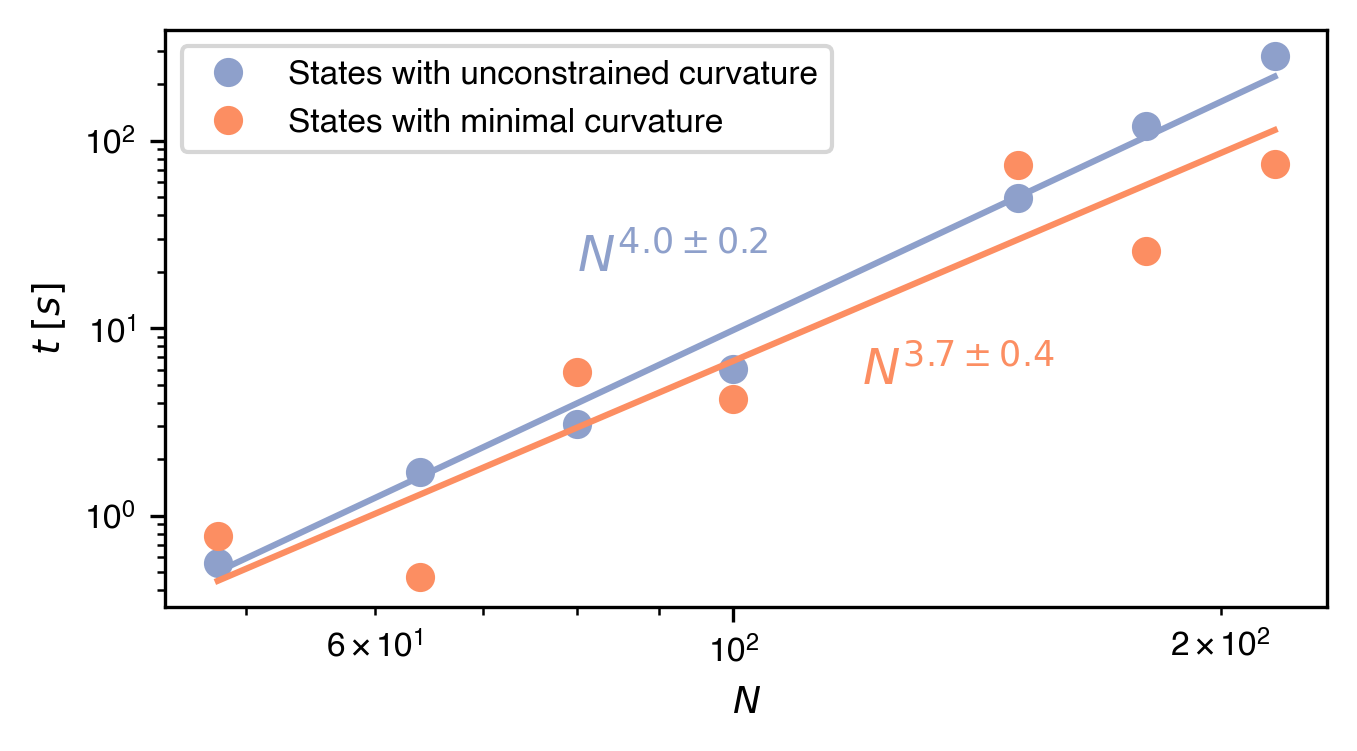

Supplement: Supplementary file 2 — Data file S1 [file sciadv.adi0204_data_file_s1.zip › Data_related_to_Main_text_figures/Figure_4/Figure_4.png]

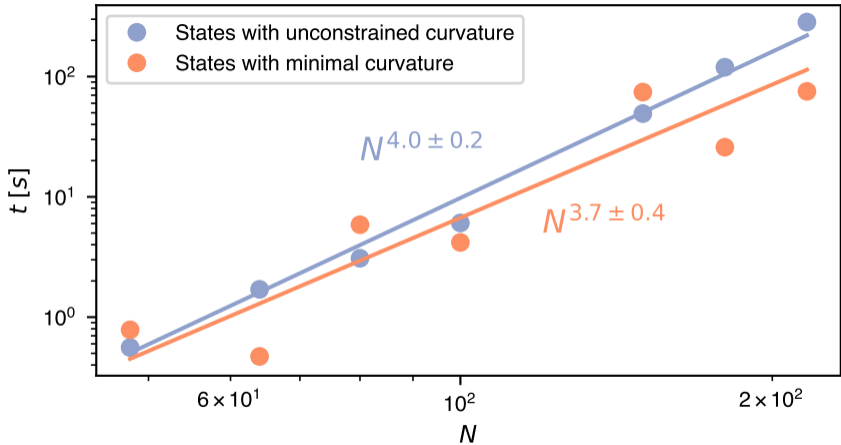

Supplement: Supplementary file 2 — Data file S1 [file sciadv.adi0204_data_file_s1.zip › Data_related_to_Main_text_figures/Figure_4/Figure_4.pdf]

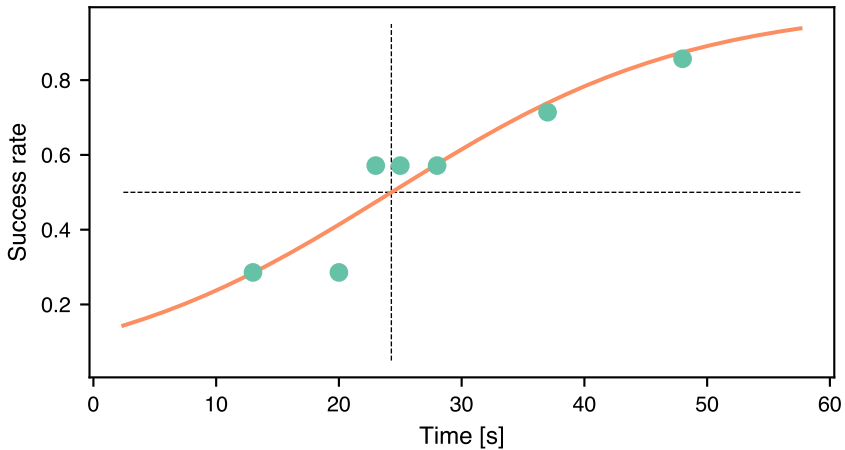

Supplement: Supplementary file 2 — Data file S1 [file sciadv.adi0204_data_file_s1.zip › Data_related_to_Supplementary_Material/Fig_S12/Hybrid_success_rate_L6x6x6_Quantum.pdf]

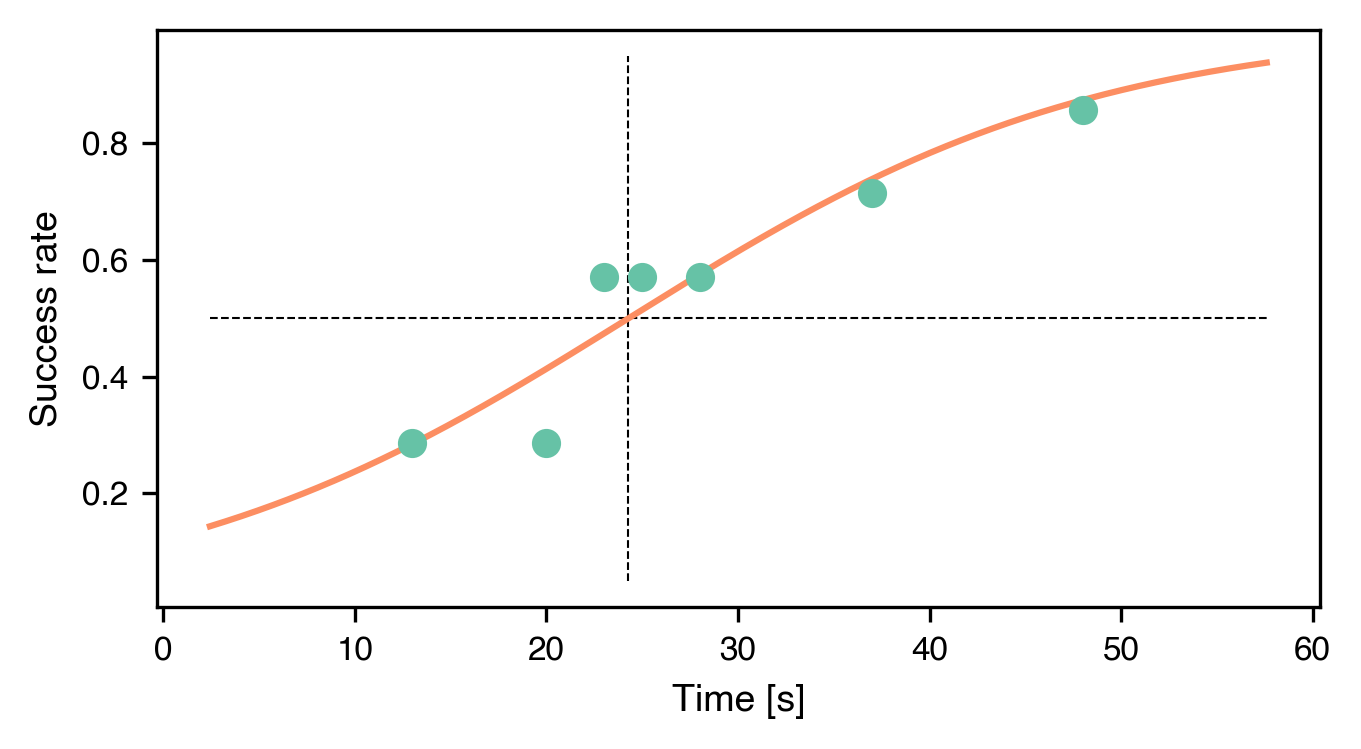

Supplement: Supplementary file 2 — Data file S1 [file sciadv.adi0204_data_file_s1.zip › Data_related_to_Supplementary_Material/Fig_S12/Hybrid_success_rate_L6x6x6_Quantum.png]

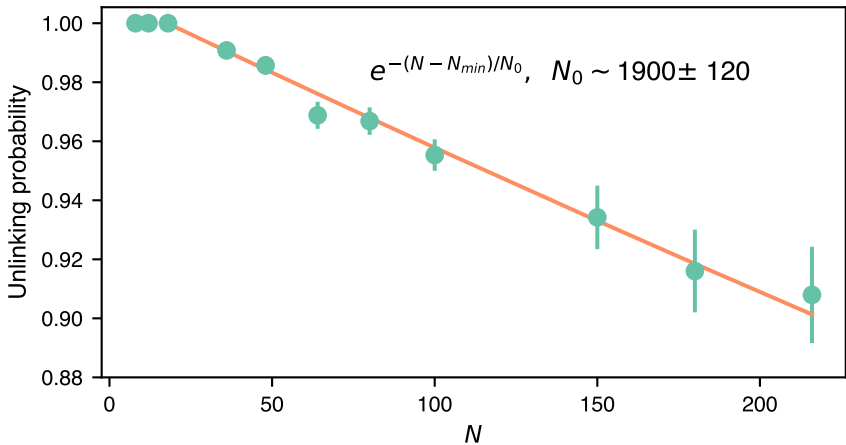

Supplement: Supplementary file 2 — Data file S1 [file sciadv.adi0204_data_file_s1.zip › Data_related_to_Supplementary_Material/Fig_S7/Unlinking_probability_2_rings.pdf]

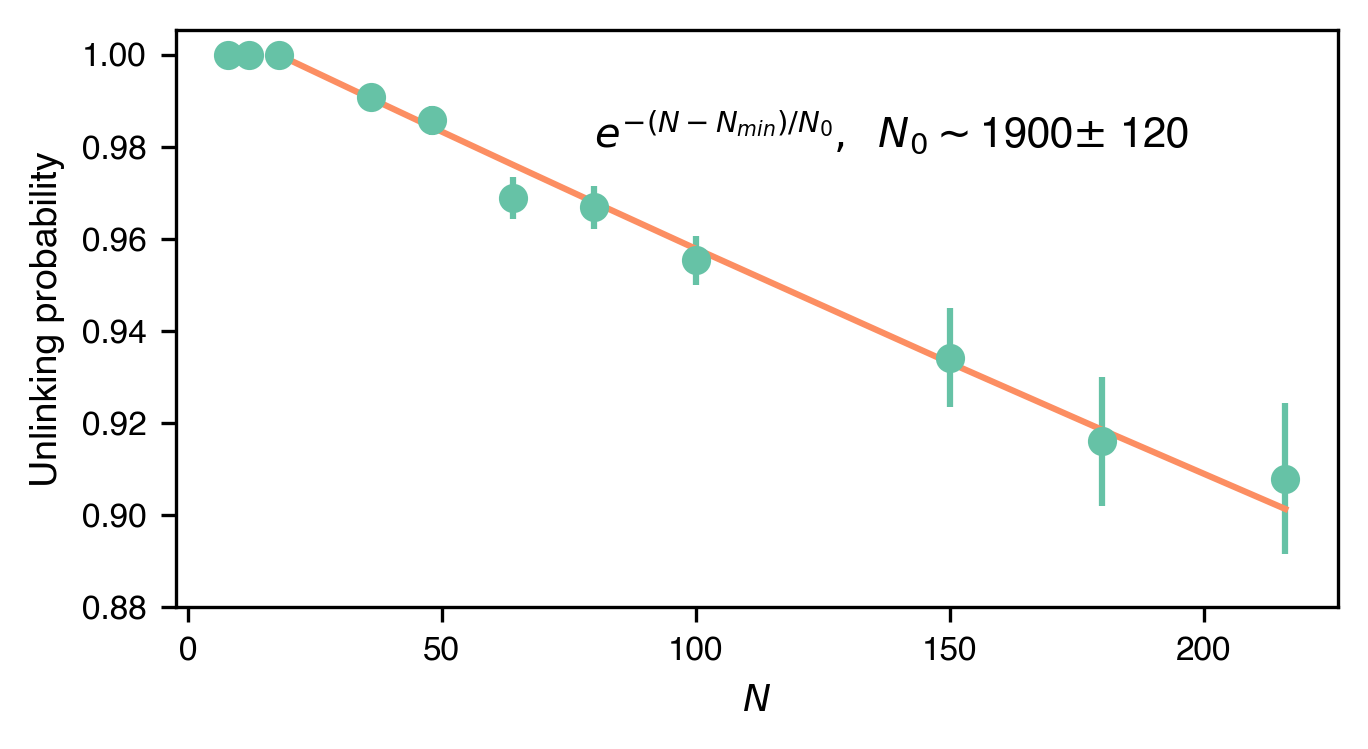

Supplement: Supplementary file 2 — Data file S1 [file sciadv.adi0204_data_file_s1.zip › Data_related_to_Supplementary_Material/Fig_S7/Unlinking_probability_2_rings.png]

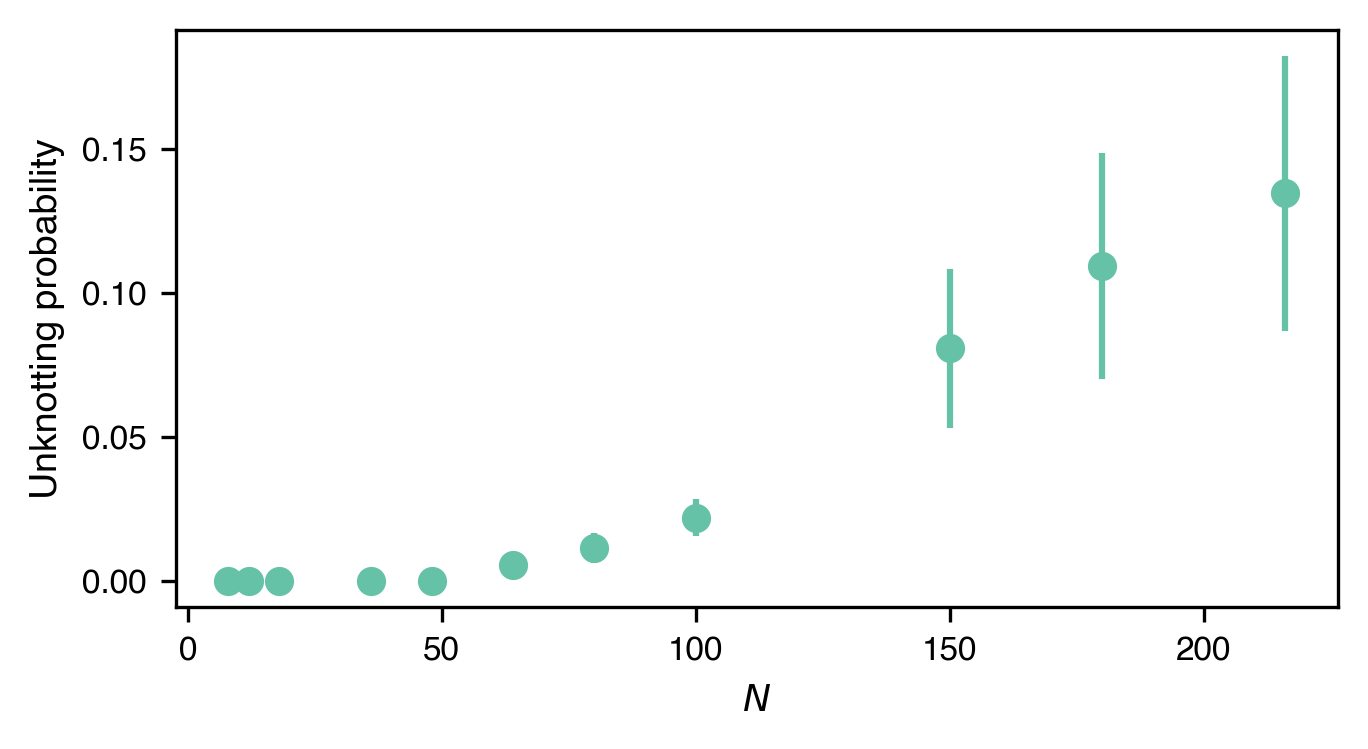

Supplement: Supplementary file 2 — Data file S1 [file sciadv.adi0204_data_file_s1.zip › Data_related_to_Supplementary_Material/Fig_S9/Knotting_probability_1_ring_vs_N.png]

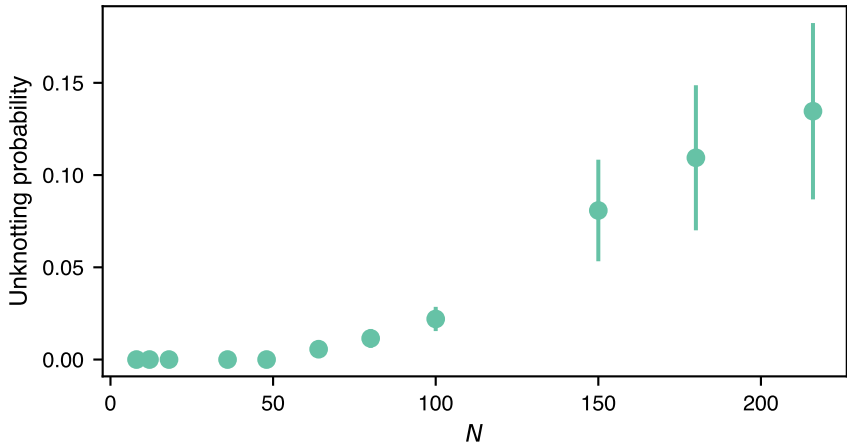

Supplement: Supplementary file 2 — Data file S1 [file sciadv.adi0204_data_file_s1.zip › Data_related_to_Supplementary_Material/Fig_S9/Knotting_probability_1_ring_vs_N.pdf]

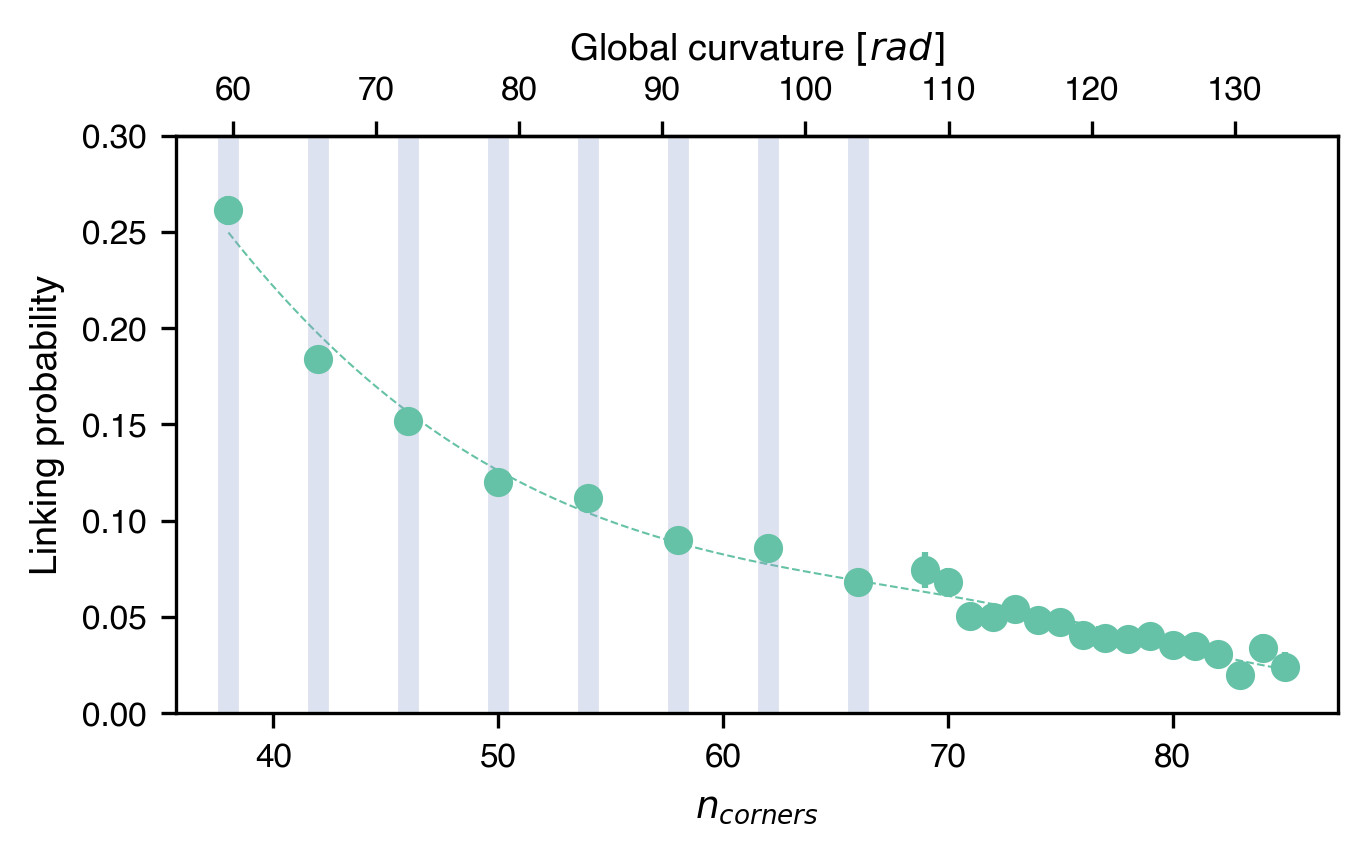

Supplement: Supplementary file 2 — Data file S1 [file sciadv.adi0204_data_file_s1.zip › Data_related_to_Supplementary_Material/Fig_S8/Linking_probability_vs_nc_2_rings.png]

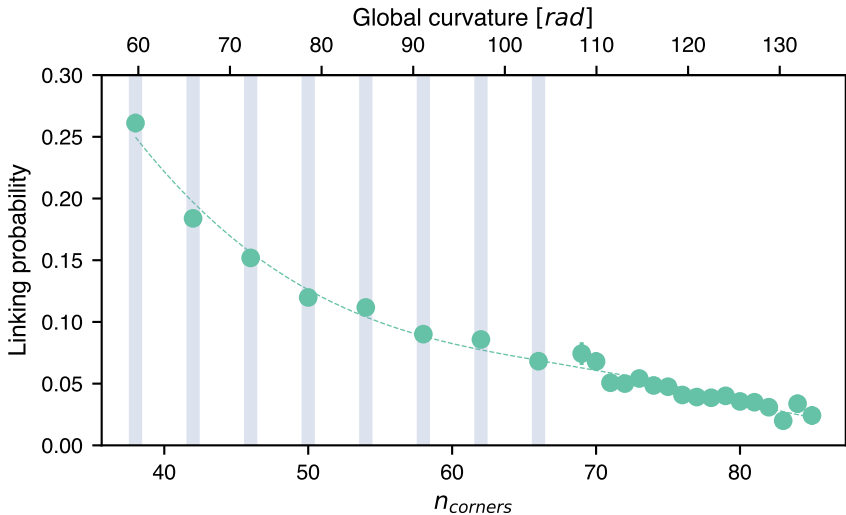

Supplement: Supplementary file 2 — Data file S1 [file sciadv.adi0204_data_file_s1.zip › Data_related_to_Supplementary_Material/Fig_S8/Linking_probability_vs_nc_2_rings.pdf]

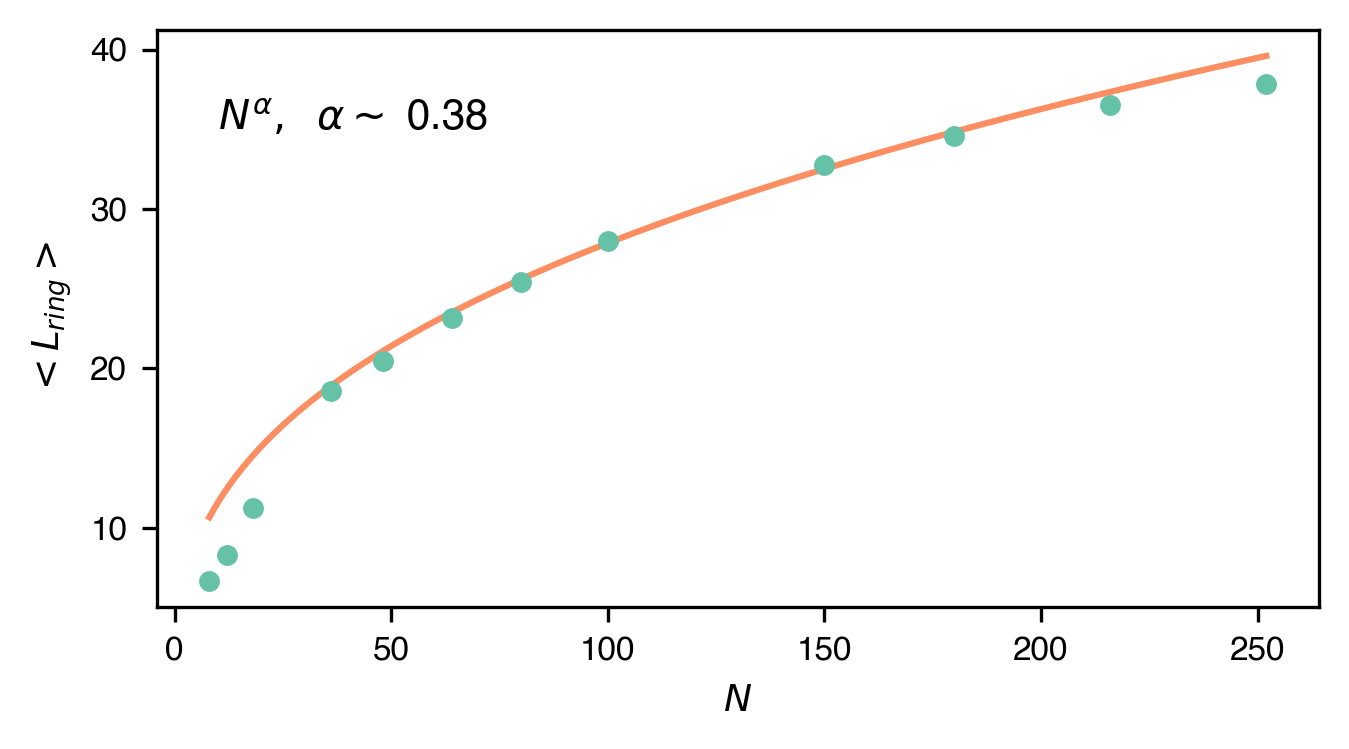

Supplement: Supplementary file 2 — Data file S1 [file sciadv.adi0204_data_file_s1.zip › Data_related_to_Supplementary_Material/Fig_S6/Length_vs_volume.png]

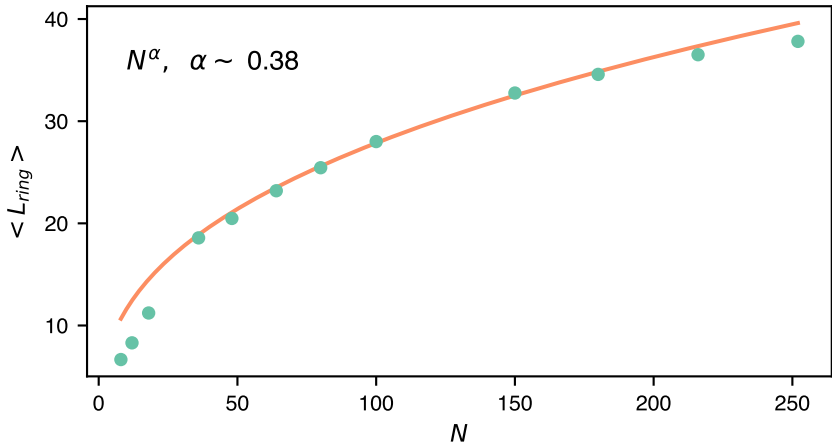

Supplement: Supplementary file 2 — Data file S1 [file sciadv.adi0204_data_file_s1.zip › Data_related_to_Supplementary_Material/Fig_S6/Length_vs_volume.pdf]

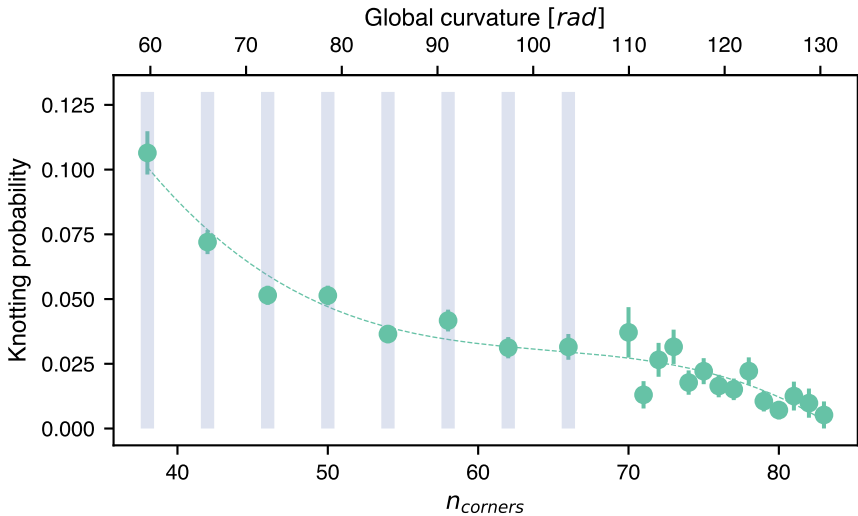

Supplement: Supplementary file 2 — Data file S1 [file sciadv.adi0204_data_file_s1.zip › Data_related_to_Supplementary_Material/Fig_S10/Knotting_probability_1_ring.pdf]

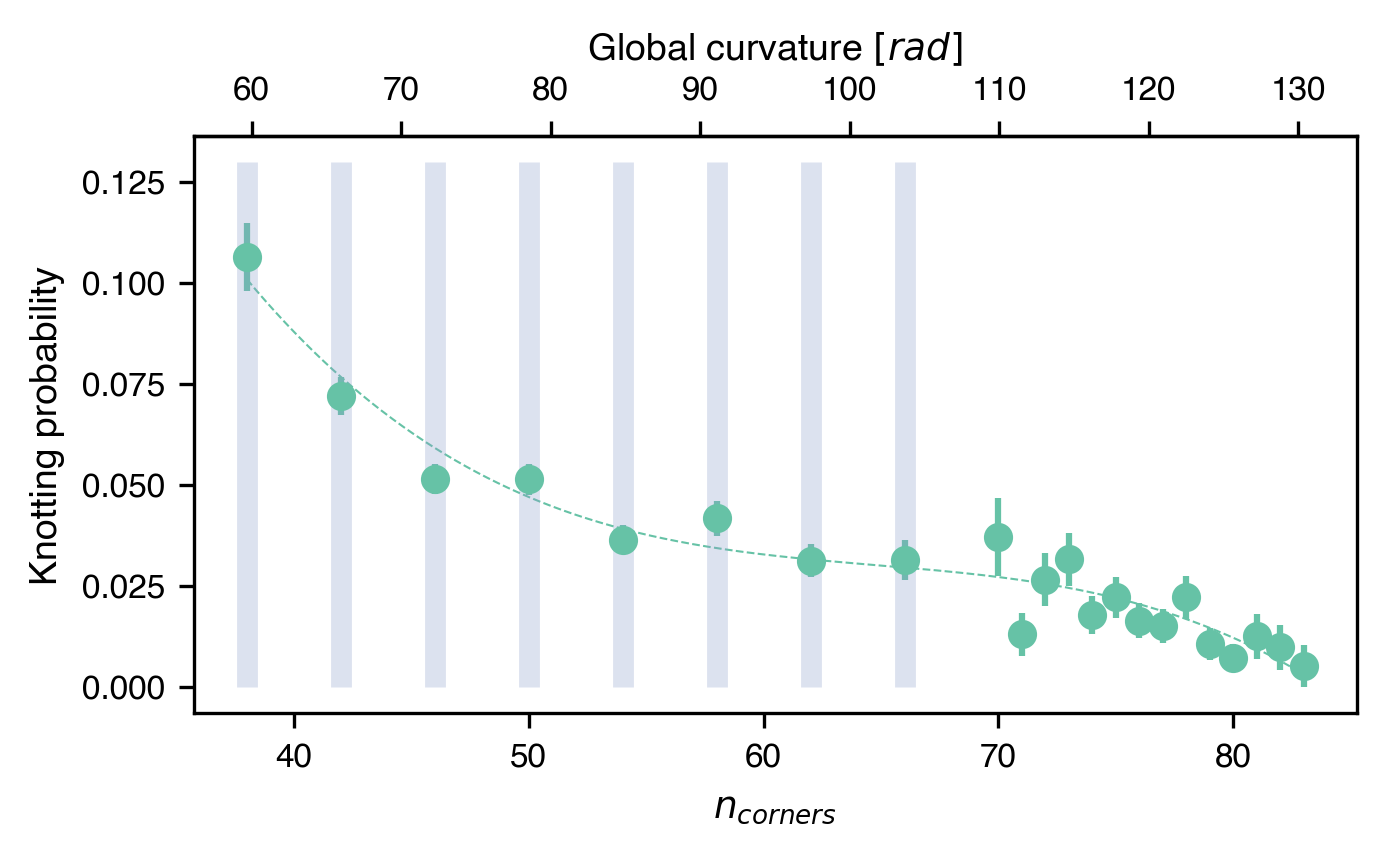

Supplement: Supplementary file 2 — Data file S1 [file sciadv.adi0204_data_file_s1.zip › Data_related_to_Supplementary_Material/Fig_S10/Knotting_probability_1_ring.png]

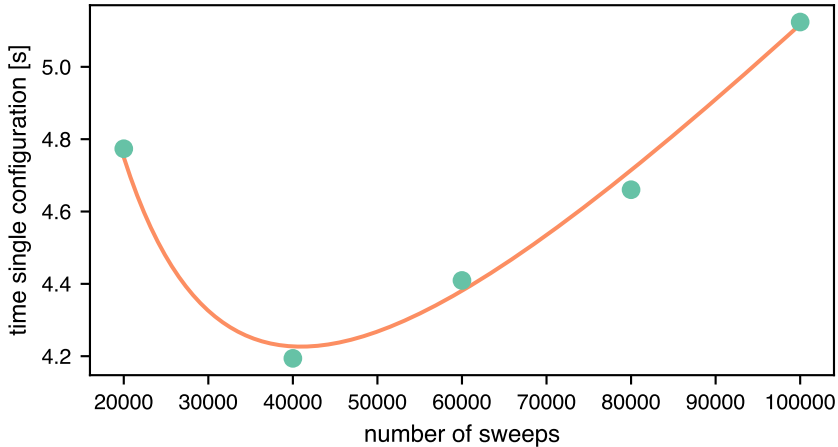

Supplement: Supplementary file 2 — Data file S1 [file sciadv.adi0204_data_file_s1.zip › Data_related_to_Supplementary_Material/Fig_S11/L5x5x4_optimal_time.pdf]

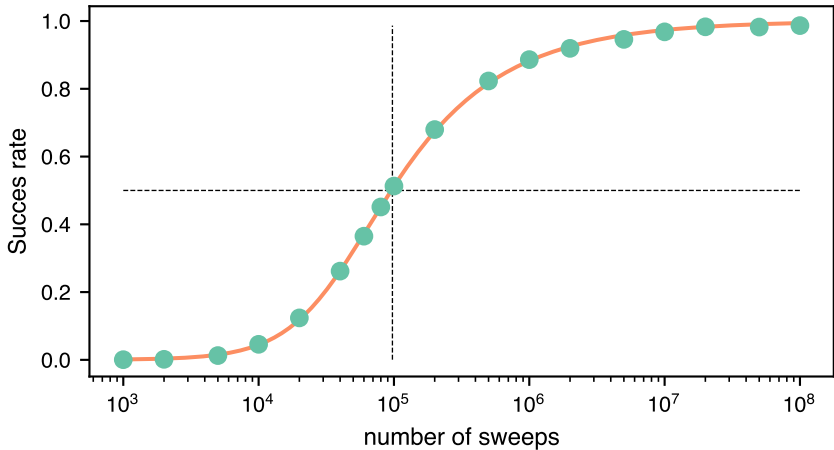

Supplement: Supplementary file 2 — Data file S1 [file sciadv.adi0204_data_file_s1.zip › Data_related_to_Supplementary_Material/Fig_S11/L5x5x4_success_rate.pdf]

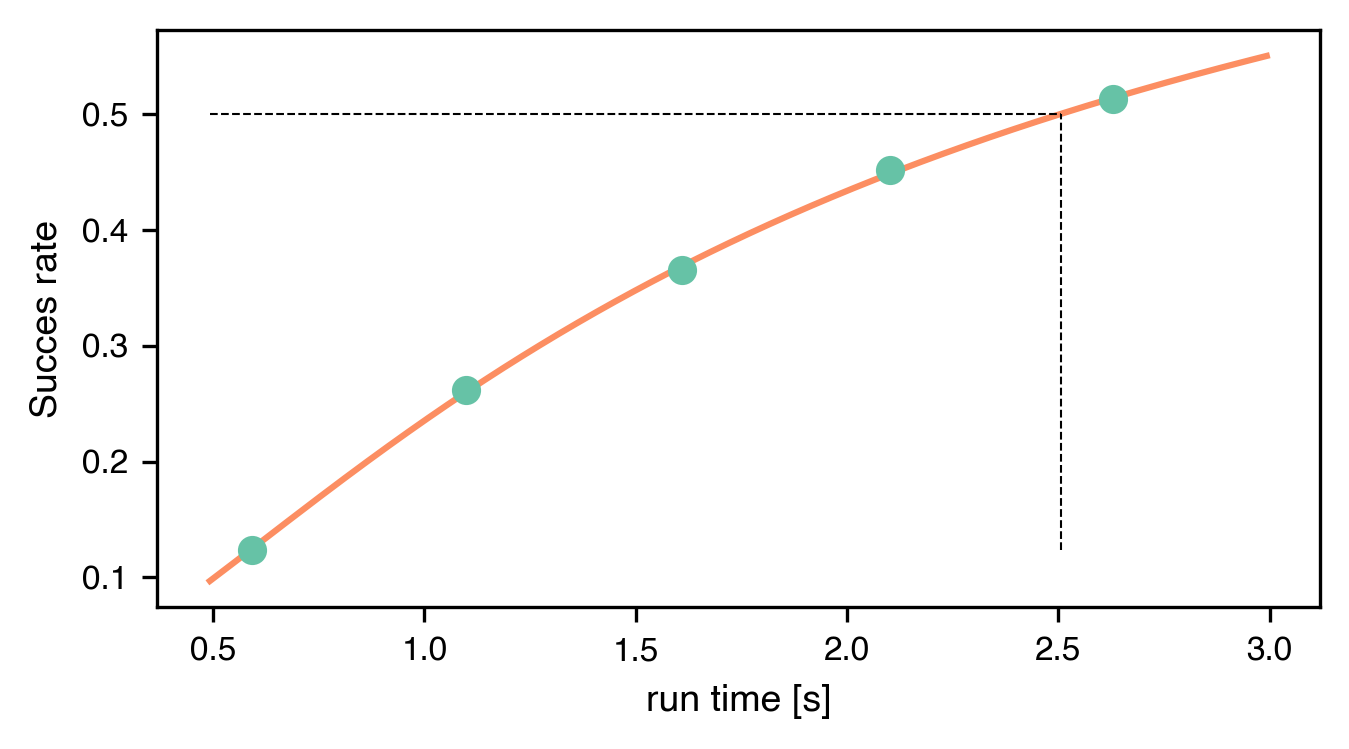

Supplement: Supplementary file 2 — Data file S1 [file sciadv.adi0204_data_file_s1.zip › Data_related_to_Supplementary_Material/Fig_S11/L5x5x4_time_zoom.png]

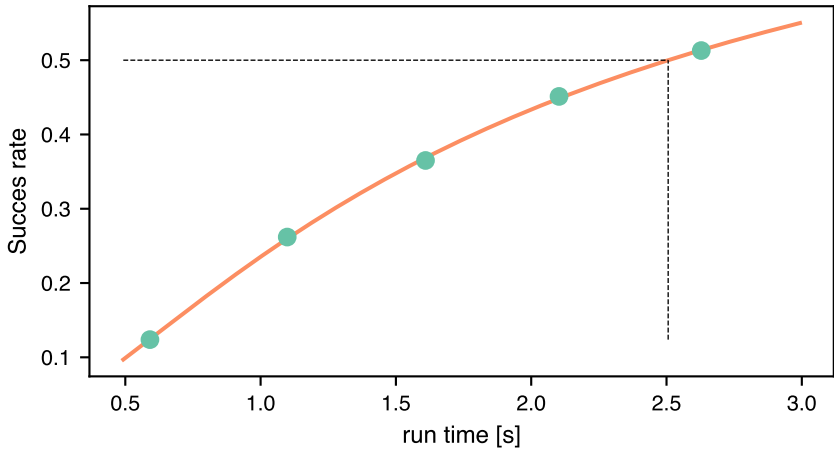

Supplement: Supplementary file 2 — Data file S1 [file sciadv.adi0204_data_file_s1.zip › Data_related_to_Supplementary_Material/Fig_S11/L5x5x4_time_zoom.pdf]

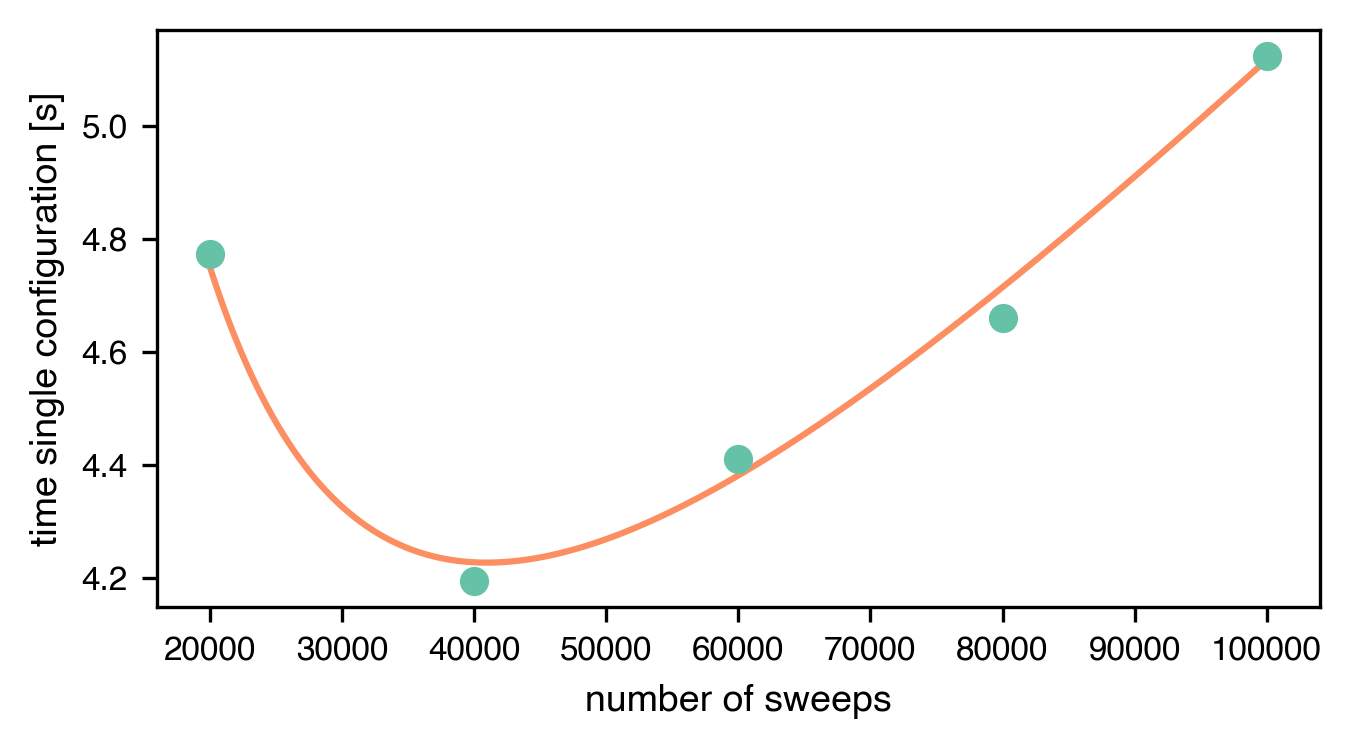

Supplement: Supplementary file 2 — Data file S1 [file sciadv.adi0204_data_file_s1.zip › Data_related_to_Supplementary_Material/Fig_S11/L5x5x4_optimal_time.png]

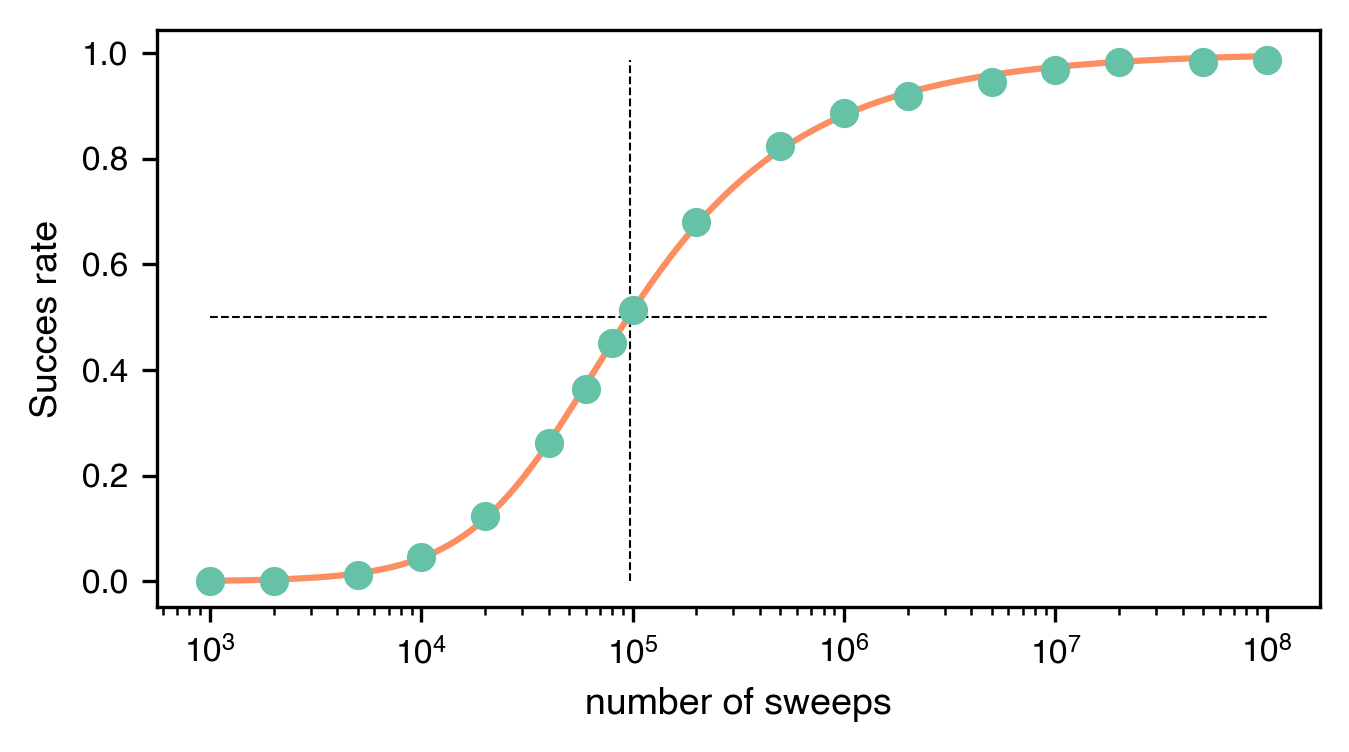

Supplement: Supplementary file 2 — Data file S1 [file sciadv.adi0204_data_file_s1.zip › Data_related_to_Supplementary_Material/Fig_S11/L5x5x4_success_rate.png]

$$A_{curv} = 0.25$$

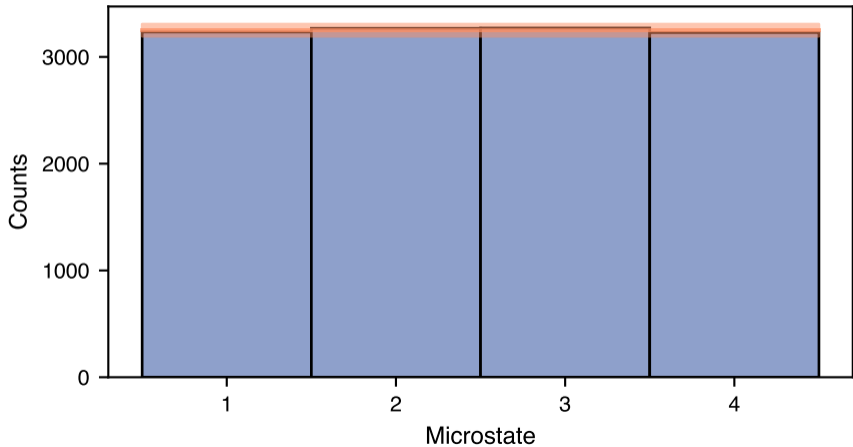

Supplement: Supplementary file 2 — Data file S1 [file sciadv.adi0204_data_file_s1.zip › Data_related_to_Supplementary_Material/Fig_S3/Histogram_ergodicity_L3x2x2_nc_8_A_0.25.pdf]

$$A_{\text{curv}} = 1$$

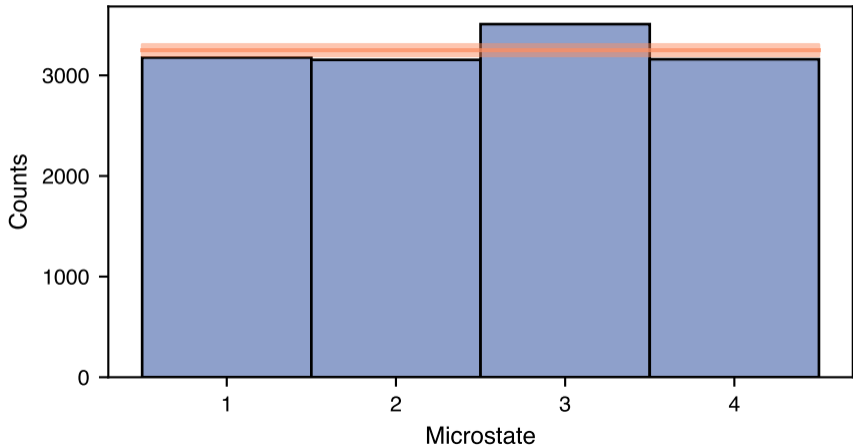

Supplement: Supplementary file 2 — Data file S1 [file sciadv.adi0204_data_file_s1.zip › Data_related_to_Supplementary_Material/Fig_S3/Histogram_ergodicity_L3x2x2_nc_8_A_1.pdf]

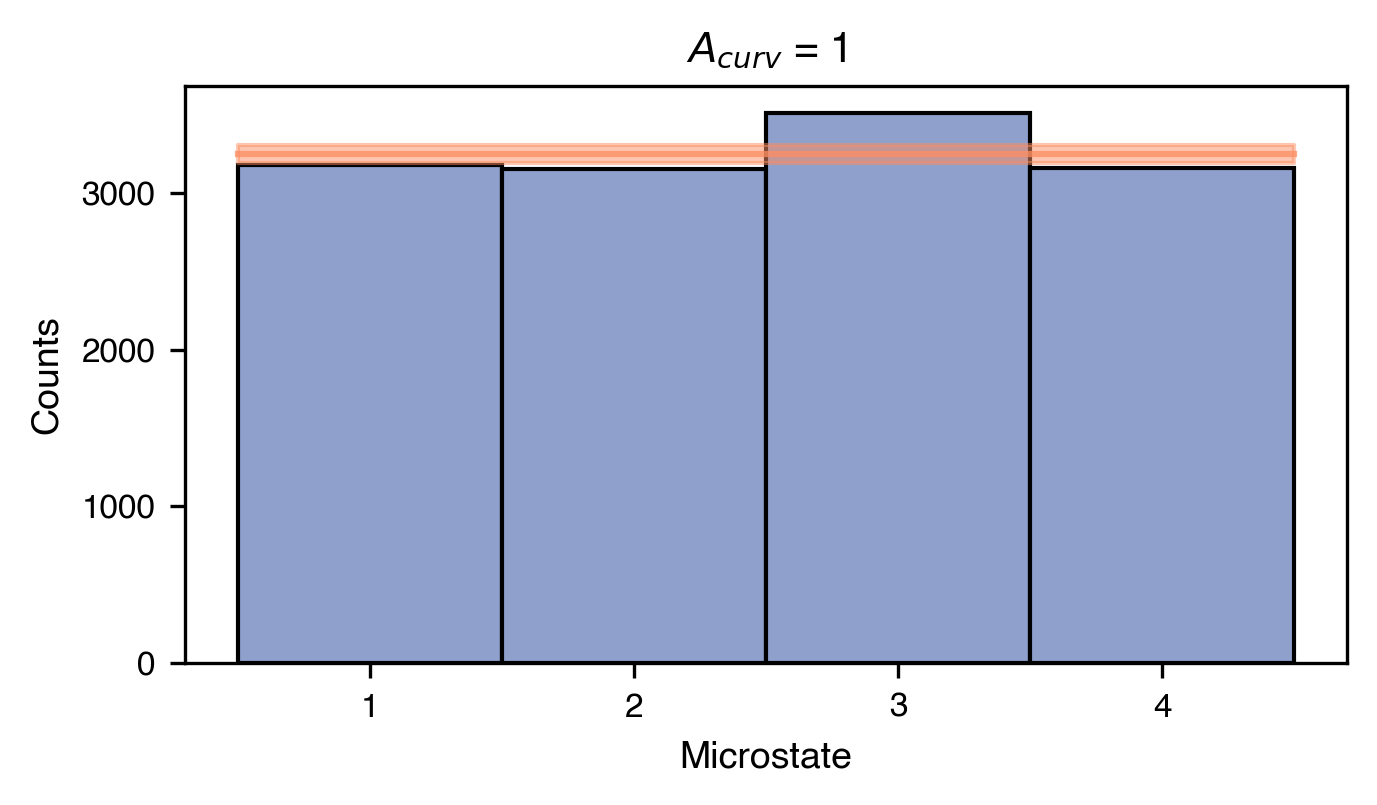

Supplement: Supplementary file 2 — Data file S1 [file sciadv.adi0204_data_file_s1.zip › Data_related_to_Supplementary_Material/Fig_S3/Histogram_ergodicity_L3x2x2_nc_8_A_1.png]

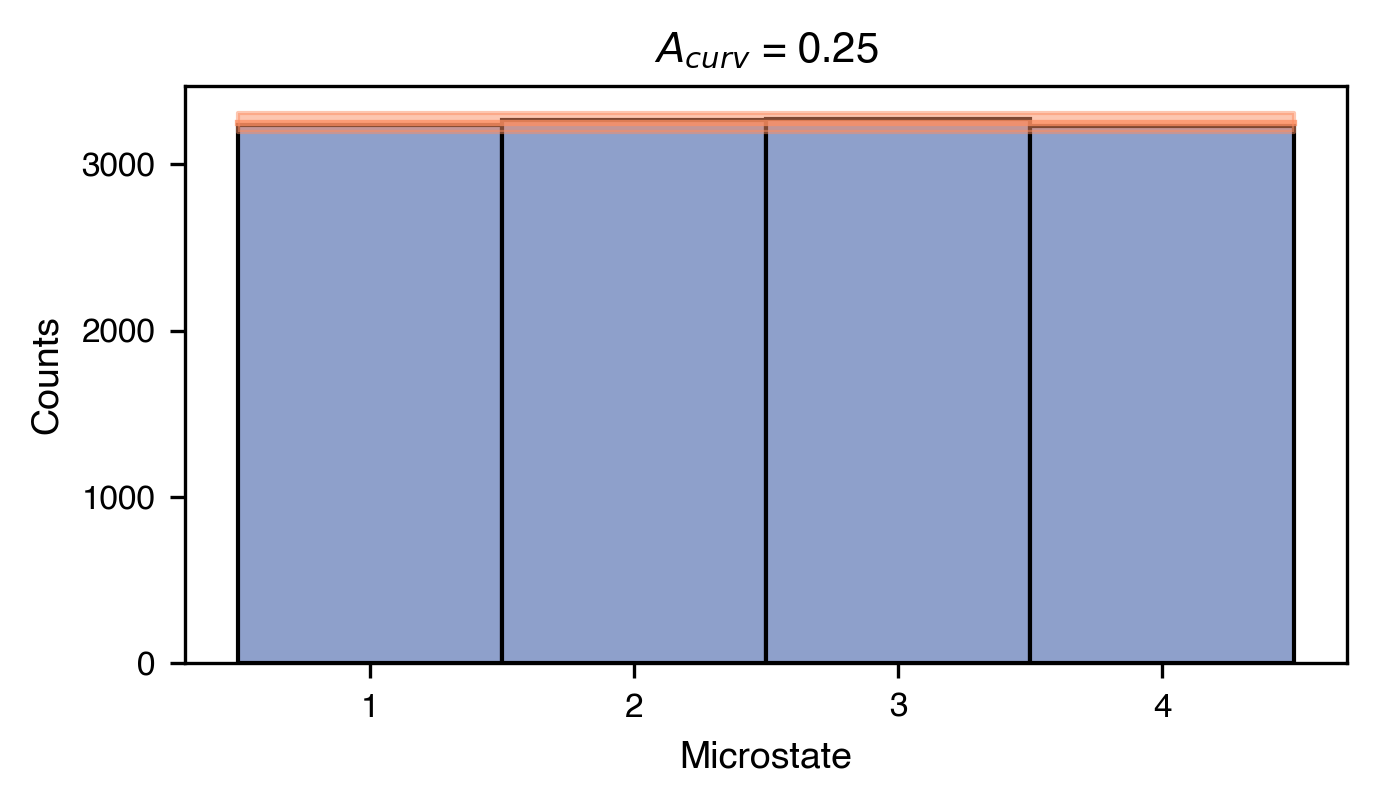

Supplement: Supplementary file 2 — Data file S1 [file sciadv.adi0204_data_file_s1.zip › Data_related_to_Supplementary_Material/Fig_S3/Histogram_ergodicity_L3x2x2_nc_8_A_0.25.png]

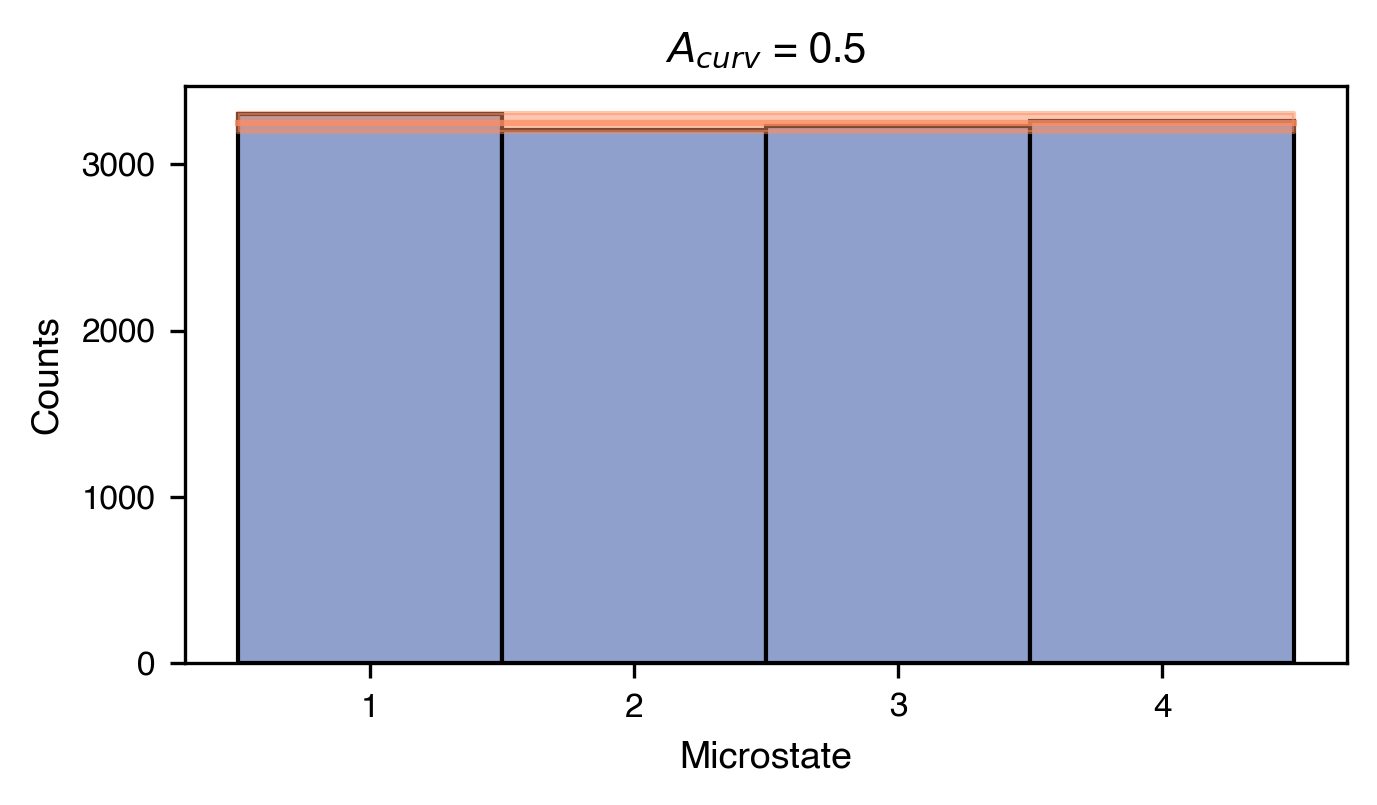

Supplement: Supplementary file 2 — Data file S1 [file sciadv.adi0204_data_file_s1.zip › Data_related_to_Supplementary_Material/Fig_S3/Histogram_ergodicity_L3x2x2_nc_8_A_0.5.png]

$$A_{curv} = 0.5$$

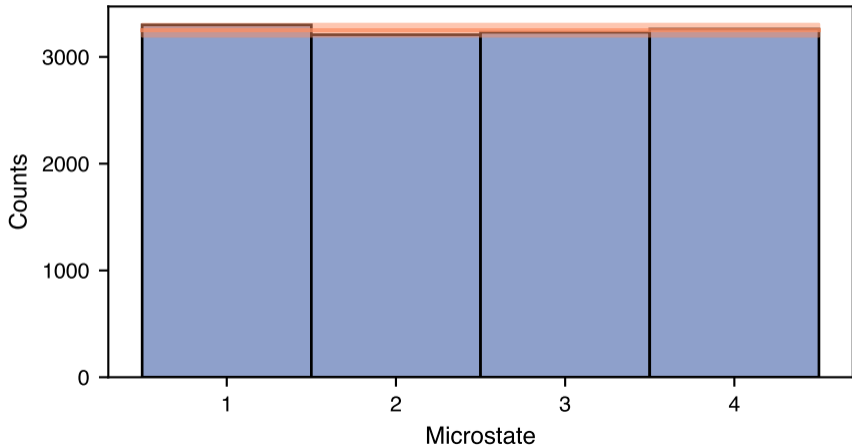

Supplement: Supplementary file 2 — Data file S1 [file sciadv.adi0204_data_file_s1.zip › Data_related_to_Supplementary_Material/Fig_S3/Histogram_ergodicity_L3x2x2_nc_8_A_0.5.pdf]

$$A_{curv} = 0.75$$

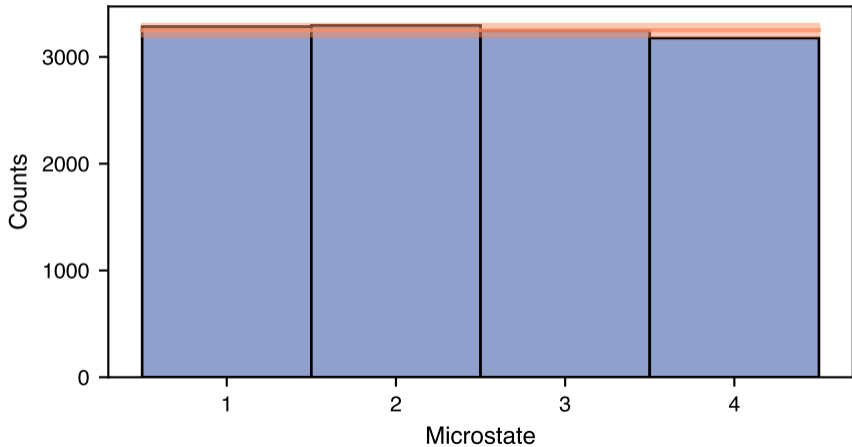

Supplement: Supplementary file 2 — Data file S1 [file sciadv.adi0204_data_file_s1.zip › Data_related_to_Supplementary_Material/Fig_S3/Histogram_ergodicity_L3x2x2_nc_8_A_0.75.pdf]

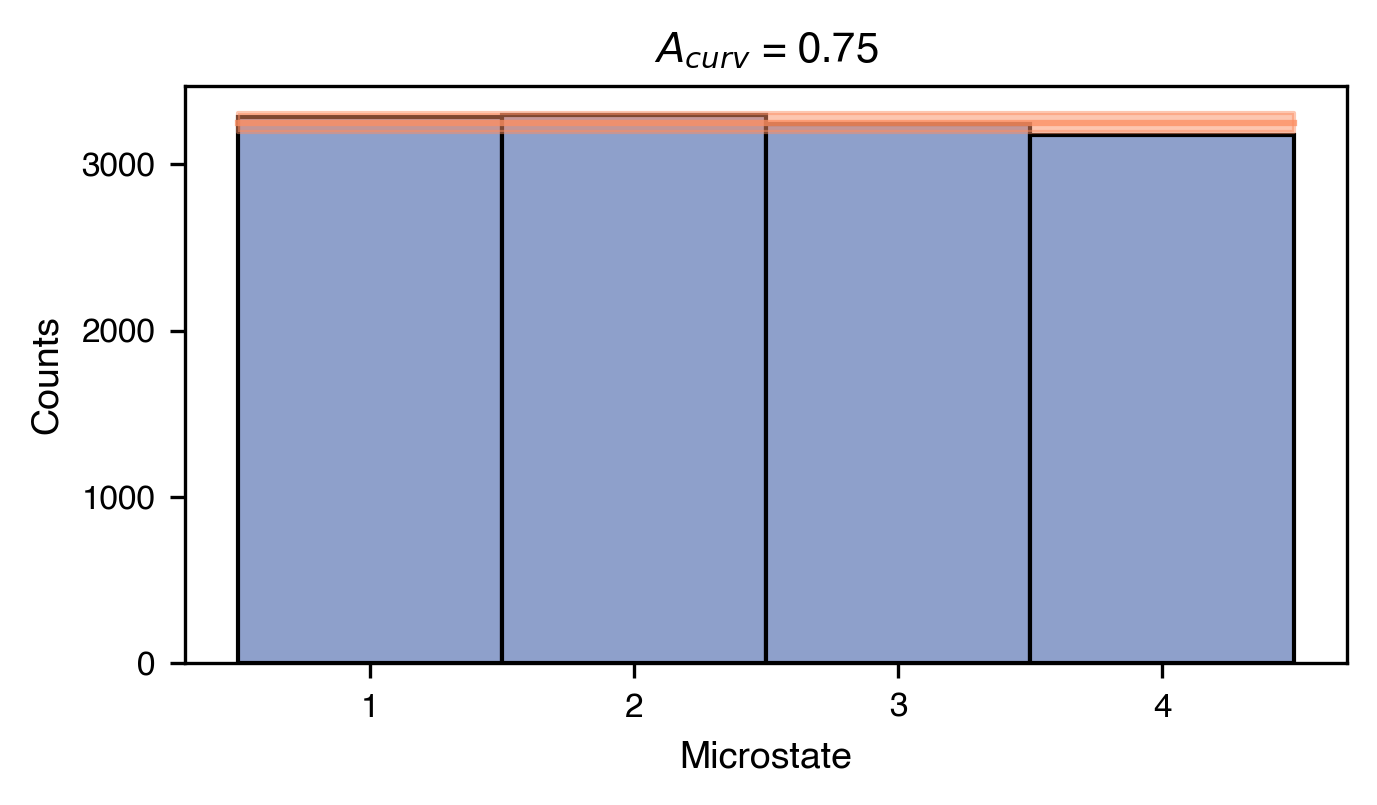

Supplement: Supplementary file 2 — Data file S1 [file sciadv.adi0204_data_file_s1.zip › Data_related_to_Supplementary_Material/Fig_S3/Histogram_ergodicity_L3x2x2_nc_8_A_0.75.png]

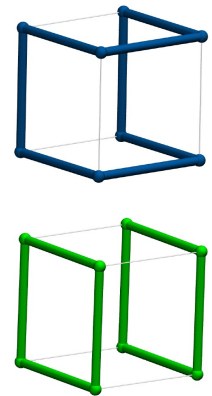

(A)

2x2x2 Lattice

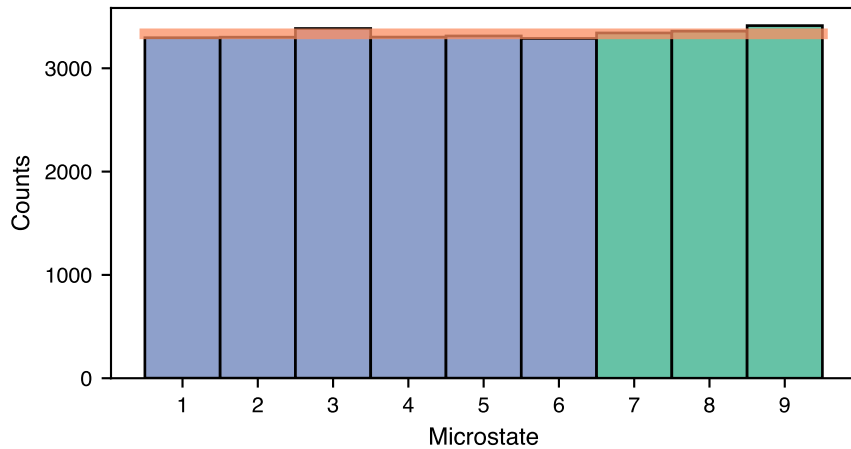

(B)

3x2x2 Lattice

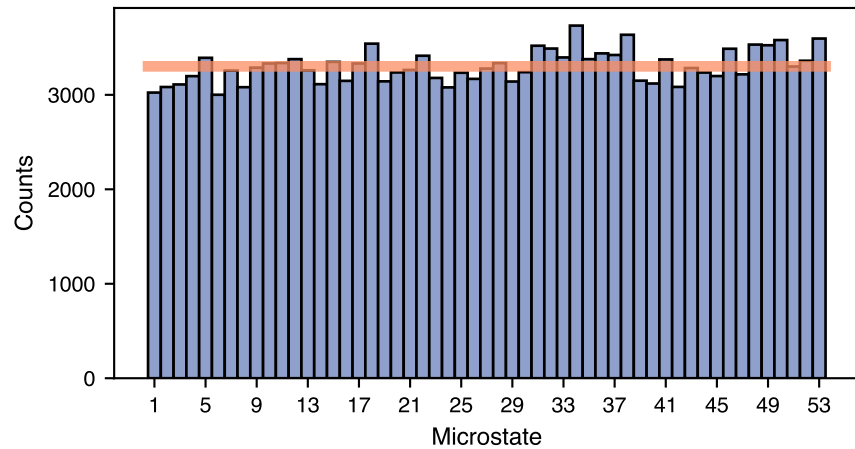

Supplement: Supplementary file 2 — Data file S1 [file sciadv.adi0204_data_file_s1.zip › Data_related_to_Supplementary_Material/Fig_S2/Ergodicity2x2x2_3x2x2.pdf]

## Slide 1
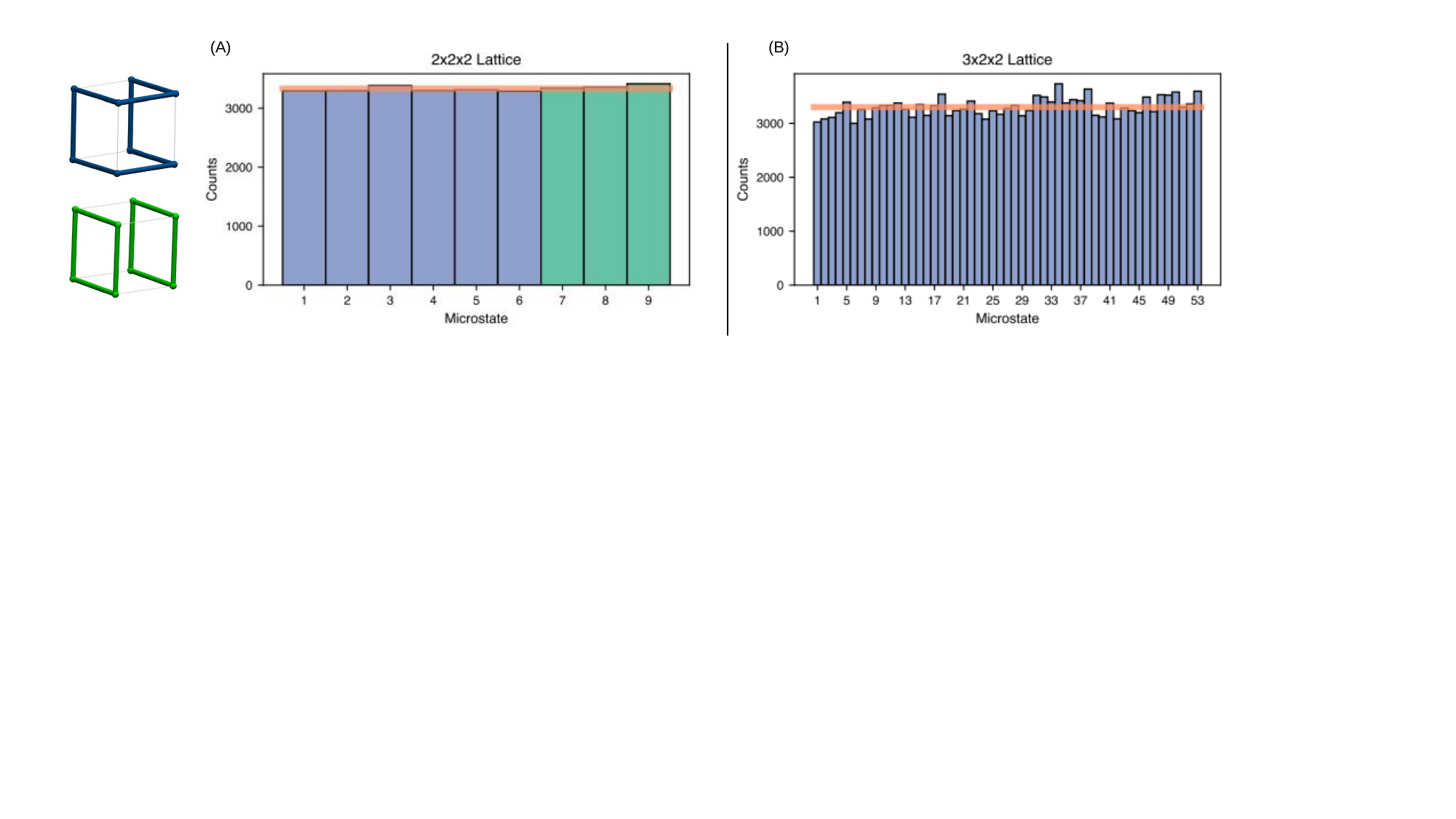

(A)
(B)

Supplement: Supplementary file 2 — Data file S1 [file sciadv.adi0204_data_file_s1.zip › Data_related_to_Supplementary_Material/Fig_S2/Ergodicity2x2x2_3x2x2.pptx]

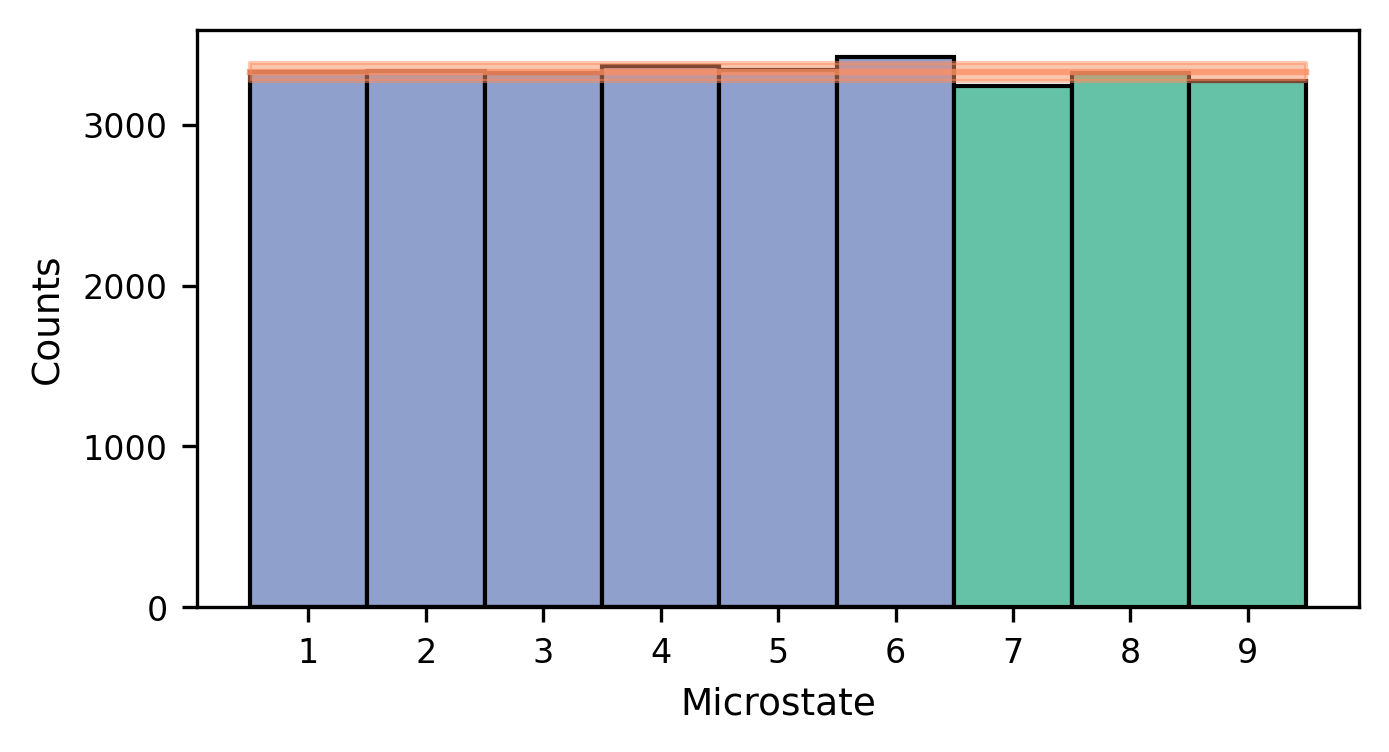

Supplement: Supplementary file 2 — Data file S1 [file sciadv.adi0204_data_file_s1.zip › Data_related_to_Supplementary_Material/Fig_S5/Histogram_uniformity_quantum.png]

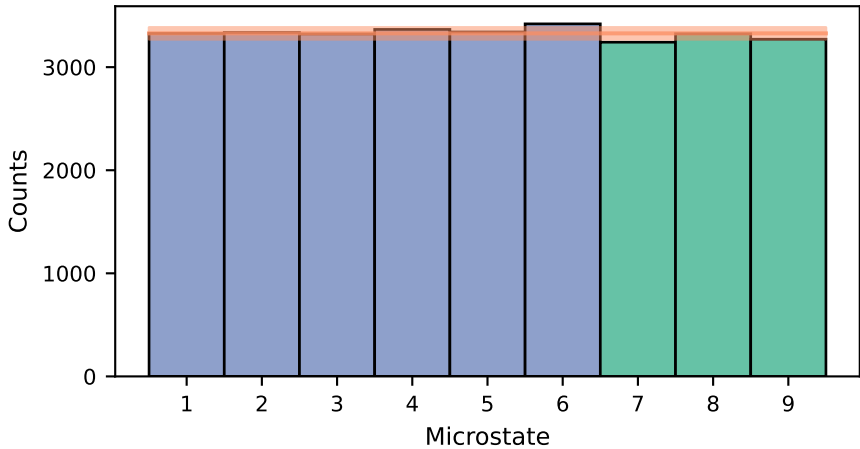

Supplement: Supplementary file 2 — Data file S1 [file sciadv.adi0204_data_file_s1.zip › Data_related_to_Supplementary_Material/Fig_S5/Histogram_uniformity_quantum.pdf]

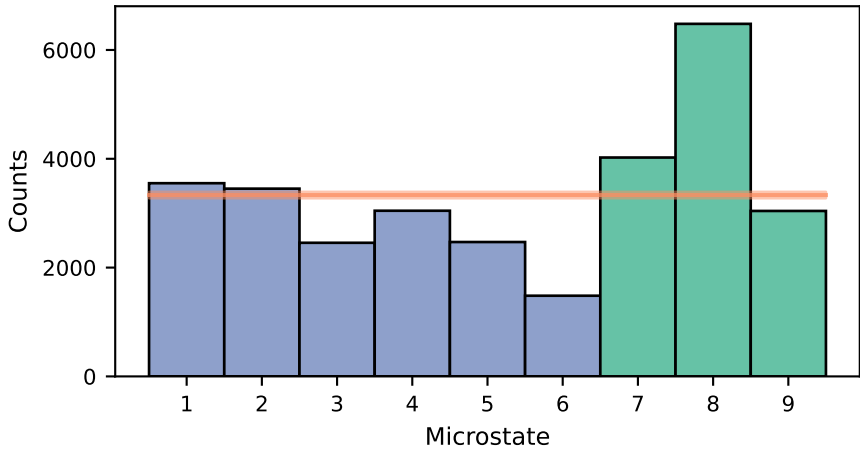

Supplement: Supplementary file 2 — Data file S1 [file sciadv.adi0204_data_file_s1.zip › Data_related_to_Supplementary_Material/Fig_S4/CPLEX/Histogram_ergodicity_L2x2x2_CPLEX.pdf]

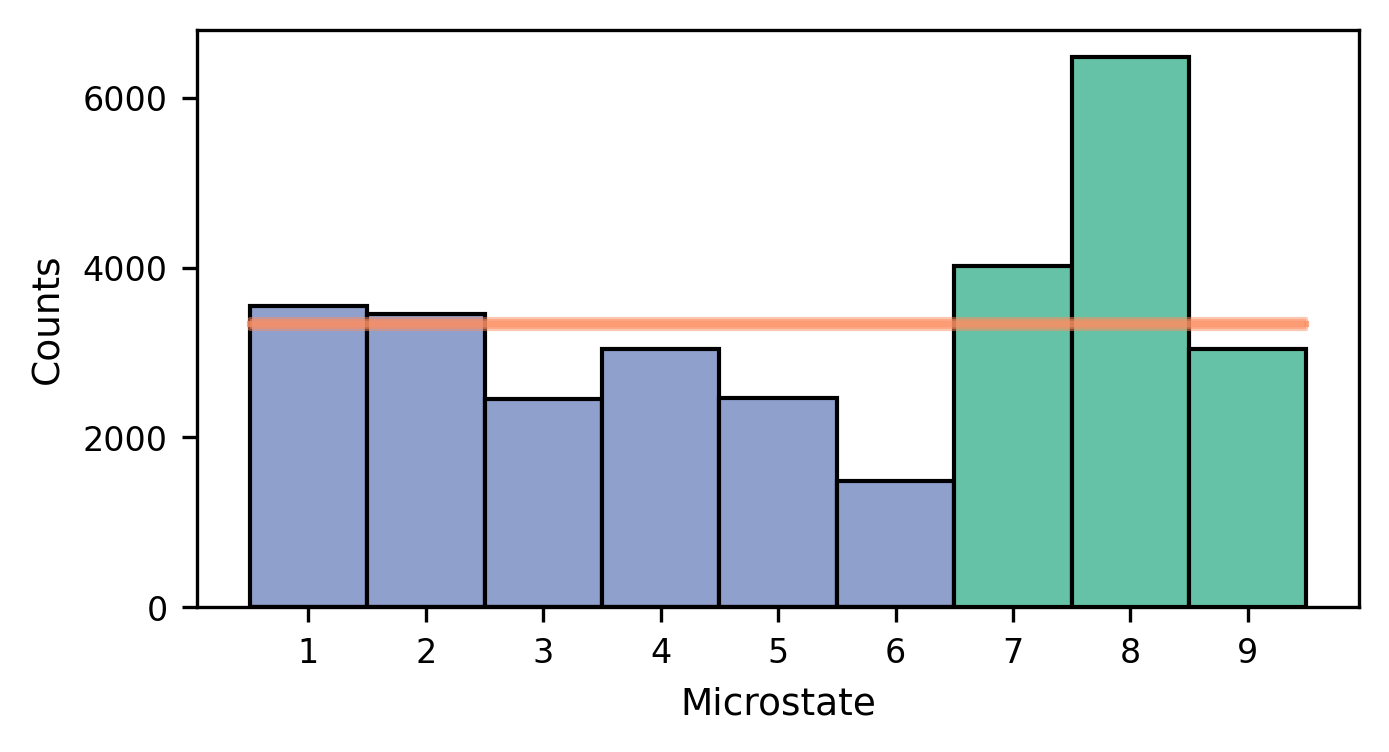

Supplement: Supplementary file 2 — Data file S1 [file sciadv.adi0204_data_file_s1.zip › Data_related_to_Supplementary_Material/Fig_S4/CPLEX/Histogram_ergodicity_L2x2x2_CPLEX.png]

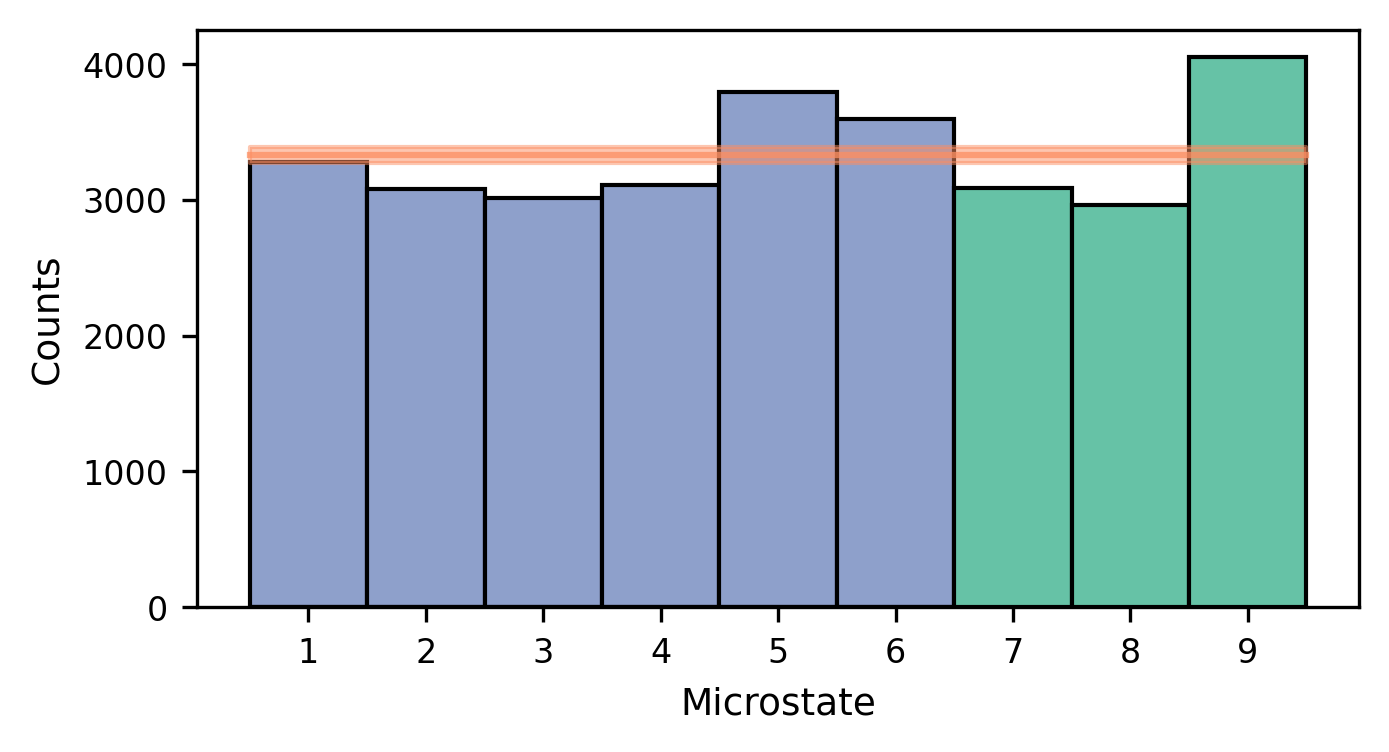

Supplement: Supplementary file 2 — Data file S1 [file sciadv.adi0204_data_file_s1.zip › Data_related_to_Supplementary_Material/Fig_S4/Gurobi/Histogram_ergodicity_L2x2x2_Gurobi.png]

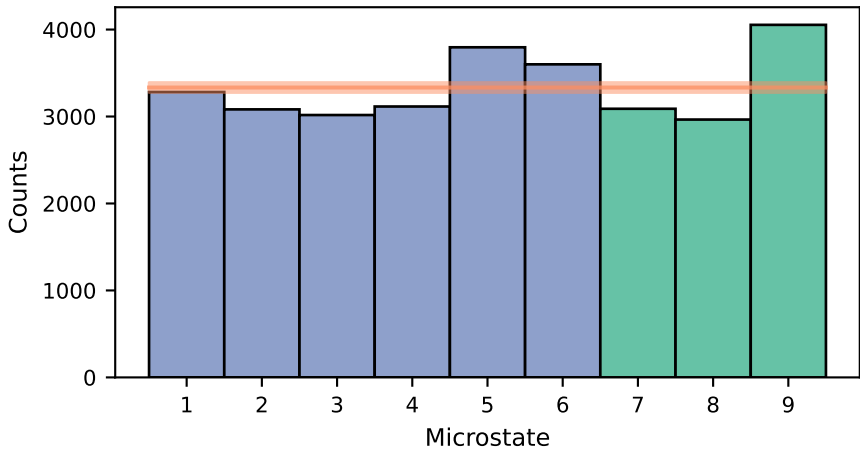

Supplement: Supplementary file 2 — Data file S1 [file sciadv.adi0204_data_file_s1.zip › Data_related_to_Supplementary_Material/Fig_S4/Gurobi/Histogram_ergodicity_L2x2x2_Gurobi.pdf]
